# The Impact of Government Lockdowns on the Mental Health of the General Population: A Systematic Review and Meta-analysis

**DOI:** 10.7759/cureus.83249

**Published:** 2025-04-30

**Authors:** Yuji Okazaki, Yasushi Tsujimoto, Kohei Yamada, Natsumi Saka, Takashi Ariie, Shunsuke Taito, Masahiro Banno, Yuki Kataoka, Norio Watanabe

**Affiliations:** 1 Department of Emergency Medicine, Hiroshima City Hiroshima Citizens Hospital, Hiroshima, JPN; 2 Department of Systematic Reviews, Scientific Research Works Peer Support Group (SRWS-PSG), Osaka, JPN; 3 Department of Internal and Family Medicine, Oku Medical Clinic, Osaka, JPN; 4 Department of Health Promotion and Human Behavior, School of Public Health, Kyoto University Graduate School of Medicine, Kyoto, JPN; 5 Department of Immunology and Microbiology, National Defense Medical College Hospital, Tokorozawa, JPN; 6 Department of Orthopaedic Surgery, Teikyo University School of Medicine, Tokyo, JPN; 7 Department of Physical Therapy, School of Health Sciences, International University of Health and Welfare, Fukuoka, JPN; 8 Division of Rehabilitation, Hiroshima University Hospital, Hiroshima, JPN; 9 Department of Psychiatry, Seichiryo Hospital, Nagoya, JPN; 10 Department of Healthcare Epidemiology, School of Public Health, Kyoto University Graduate School of Medicine, Kyoto, JPN; 11 Department of Internal Medicine, Kyoto Min-iren Asukai Hospital, Kyoto, JPN; 12 Department of Psychiatry, Soseikai General Hospital, Kyoto, JPN

**Keywords:** covid-19 outbreak, emerging infectious diseases, general population, lockdown, public mental health

## Abstract

Since December 2019, the COVID-19 pandemic has spread globally, prompting governments in many countries to implement lockdowns to control the transmission of the virus. Outbreaks of emerging infectious diseases, such as COVID-19, and the associated government lockdowns may have significant negative impacts on mental health. A comprehensive review of the available evidence on this topic can provide useful information for policymakers. This review aimed to assess the effects of government lockdowns on the mental health of the general population during emerging infectious disease outbreaks.

On April 11, 2022, we conducted a systematic search of CENTRAL, MEDLINE, PsycINFO Ovid, and two clinical trial registries, supplemented by reference checking and citation searching. We included non-randomized studies of interventions (NRSIs) involving adults and adolescents, regardless of comorbidities, that examined the effects of government-imposed lockdowns compared to no lockdown during outbreaks of emerging infectious diseases, including SARS, MERS, COVID-19, H1N1, equine influenza, avian influenza, and Ebola virus disease. Critical outcomes assessed were depressive symptom severity and suicide, while important outcomes included anxiety symptom severity, post-traumatic stress disorder (PTSD) symptom severity, insomnia symptom severity, and substance use. We used the ROBINS-I tool to assess the risk of bias and conducted a meta-analysis using a random-effects model. The certainty of evidence was evaluated using the GRADE approach.

We included 42 NRSIs, all conducted during the COVID-19 pandemic. Of the 27 studies reporting depressive symptoms, we pooled effect sizes from eight studies. The findings suggest that government lockdowns may have little to no effect on depressive symptom severity within the 12-month follow-up; however, the evidence was very uncertain (standardized mean difference (SMD) 0.00, 95% CI -0.08 to 0.09; I^2^ = 70%; 11,278 participants). Two studies reported on suicide outcomes, but both had an overall critical risk of bias due to confounding; therefore, we did not synthesize results and judged the evidence as very low certainty. For anxiety symptom severity, we pooled data from five of 20 studies and found that government lockdowns may have little to no effect within the 12-month follow-up (SMD 0.08, 95% CI -0.10 to 0.26; I^2^ = 91%; 11,006 participants). Regarding PTSD symptom severity, pooled data from one of two studies suggested that government lockdowns may increase the symptom severity within the 12-month follow-up (MD 0.18, 95% CI 0.08-0.28; 1,754 participants). We pooled data from two of eight studies on insomnia symptom severity and found that government lockdowns may increase the symptom severity within the 12-month follow-up (MD 1.28, 95% CI 0.62-1.94; I^2^ = 91%; 5,142 participants). In terms of alcohol use, data pooled from five of nine studies on alcohol use showed that government lockdowns may have little to no effect on alcohol consumption within the 12-month follow-up (SMD 0.03, 95% CI -0.05 to 0.11; I^2^ = 66%; 8,261 participants). Overall, the evidence regarding all important outcomes was of very low certainty.

At present, the impact of government lockdowns during emerging infectious disease outbreaks on mental health in the general population remains very uncertain. Future research should prioritize well-designed studies to better assess the mental health effects of lockdown measures during novel outbreaks.

## Introduction and background

The coronavirus disease (COVID-19) pandemic (i.e., an epidemic that spreads across several countries or continents and aﬀects a large number of people) began in December 2019 and led to the implementation of government lockdowns in many countries to curb the spread of the infection. Beyond its physical health consequences, the pandemic has also highlighted how outbreaks of emerging infectious diseases can significantly impact mental health. For instance, the severe acute respiratory syndrome (SARS) epidemic (i.e., an unexpected increase in the number of disease cases within a specific geographical area) was associated with symptoms of post-traumatic stress disorder (PTSD) and depression in the general population [1]. Similarly, the COVID-19 pandemic has resulted in reduced psychological well-being and increased symptoms of anxiety and depression [2]. These mental health outcomes may result from multiple factors, including fear of infection, grief due to illness or loss of relatives and acquaintances, and restrictions such as lockdowns or quarantine (i.e., the separation and restriction of movement of individuals who may have been exposed to a contagious disease) to prevent disease spread.

Non-pharmacological public health measures have been primary strategies in controlling not only the COVID-19 outbreak but also other emerging infectious diseases such as Ebola, Middle East respiratory syndrome (MERS), and influenza A (H1N1) [3-5]. Lockdowns (defined as large-scale government directives requiring individuals to stay at home and to refrain from, or limit, activities outside the home) have been widely adopted to reduce COVID-19 transmission [6]. Various governments have used different terms to describe the lockdown, including stay-at-home orders and movement control orders [7-9]. The extent and enforcement of lockdown measures varied across countries; some were mandatory, while others were voluntary. For example, in April 2022, the Japanese government declared a state of emergency due to the COVID-19 endemic (i.e., an outbreak consistently present but limited to a particular region), but this order was not mandatory [10]. In contrast, other countries enforced penalties for violations of lockdown regulations [11]. Given that lockdowns can disrupt employment and the ability to work, several governments provided direct economic and social assistance (such as public wage subsidies, price controls, distribution of goods, or a combination) to mitigate financial burdens [12]. Overall, lockdowns have played an effective role in controlling the spread of COVID-19 [13,14].

Forced and strict isolation measures, such as lockdowns or quarantines, can negatively impact mental health [15,16]. Lockdowns often result in reduced educational opportunities and the suspension of economic or cultural activities. With the closure of schools or the transition to online learning, some students may abandon higher education due to increased anxiety about academic performance [17]. In addition, the downturn in economic activities associated with lockdowns can lead to unemployment and heightened financial concerns [18]. At the same time, the cessation of various cultural activities may impact the well-being and quality of life of many individuals, including the elderly [19,20].

A systematic review has demonstrated that quarantine can adversely affect mental health outcomes [21]. Although both quarantine and lockdown involve isolation, their mental health impacts may differ due to variations in the populations affected, the scale of implementation, and the duration of isolation. The impact of lockdown on individuals and society as a whole is likely to be significant. Several studies have reported on the mental health impacts of government lockdowns during the COVID-19 pandemic [16,22,23]. However, comprehensive evidence regarding the long-term mental health consequences of lockdowns in the general population remains limited. Therefore, we conducted this review to evaluate the effects of government lockdowns during emerging infectious disease outbreaks on the mental health of the general population. This evidence may be instrumental for policymakers in preparing for future public health emergencies.

## Review

Methods

This review was conducted in accordance with the methodological standards outlined in the Cochrane Handbook for Systematic Reviews of Interventions and the Preferred Reporting Items for Systematic Reviews and Meta-Analyses (PRISMA) guidelines (Supplementary material 1) [24,25]. This review protocol was registered in the Cochrane Library [26].

Difference Between Protocol and Review

The following terminologies in this review were revised: “primary outcome” was changed to “critical outcome” and “secondary outcome” to “important outcome.” Consequently, in the “Certainty of the Evidence Assessment” section, what was previously referred to as “important outcomes” in the protocol is now termed “critical outcomes” in this review.

When multiple instruments measured the same outcome, we had planned to select the instruments of outcomes of interest that came first in our list in our protocol. However, studies that measured outcomes using multiple instruments were not identified.

In the before-and-after comparison design, we measured all the outcomes of interest in the control group (i.e., no government lockdown) during the period immediately preceding the intervention.

We excluded studies that compared periods of government lockdowns with post-lockdown periods, regardless of whether the lockdowns were lifted.

We conducted a literature search in the following electronic databases: Cochrane Central Register of Controlled Trials (CENTRAL), MEDLINE Ovid, PsycINFO Ovid, the US National Institutes of Health Ongoing Trials Register (ClinicalTrials.gov), and the WHO International Clinical Trials Registry Platform (ICTRP). The search strategy outlined in the protocol yielded a large number of search results. After consultation with Cochrane information specialists, we excluded Embase Ovid from the electronic database search due to practical considerations in conducting the review.

Although we had initially planned to assess the risk of bias in randomized controlled trials (RCTs) using the RoB 2 tool, this was not applicable, as there were no RCTs included. Furthermore, while we intended to conduct a separate meta-analysis and present the risk of bias for emerging infectious diseases, our search only yielded reports of lockdowns during the COVID-19 outbreak; therefore, we limited our review to this topic.

We could not carry out the planned subgroup analyses and sensitivity analyses due to limited data and the small number of included studies.

We assessed the publication bias based on unpublished studies in clinical trial registries and published protocols that were identified by a full-text screening because we could not assess publication bias using funnel plots.

Study Settings and Designs

We included RCTs and non-randomized studies of interventions (NRSIs) to investigate the impact of government lockdowns on mental health during outbreaks of emerging infectious diseases, including SARS, MERS, COVID-19, H1N1, equine influenza, avian influenza, and Ebola virus disease. Studies with design features outlined in the Appendices [27] were eligible for inclusion. We did not apply restrictions based on language, country of origin, year of publication, or publication status. Although our protocol initially planned for the inclusion of RCTs and studies conducted during outbreaks of emerging infectious diseases other than COVID-19, ultimately, only NRSIs conducted during the COVID-19 pandemic were identified.

Eligibility Criteria of Study Participants and Interventions

We included adults (aged 20 years and older) and adolescents (aged 10 years and older), irrespective of comorbidities, and infants and children were excluded. For studies that evaluated a general population and reported the age of study participants, we contacted the original study authors to obtain information on participants by age groups and outcomes of interests. When this was not possible, we categorized the study to have enrolled adults if ≥80% of the study participants were aged 20 years and older.

We included government lockdowns during emerging infectious disease outbreaks and excluded studies on terrorism, war, and natural or humanitarian disasters. We defined government lockdowns as national or regional isolation, as directed by the government, regardless of the individuals' exposure or likelihood of exposure to an emerging infectious disease. We accepted the definition of government lockdowns used by the study investigators. The comparator was no government lockdown. We excluded studies that compared periods of government lockdowns with post-lockdown periods, regardless of whether the lockdowns were lifted. We excluded studies on voluntary or mandatory quarantine because of infection by, or exposure to, an emerging infectious disease.

Outcome Measures

We assessed depressive symptom severity and suicide as critical outcomes. We defined suicide as death caused by a fatal self-injurious act, with some evidence of intent to die. We also assessed anxiety symptom severity, PTSD symptom severity, insomnia symptom severity, and substance use as important outcomes. We measured all of the above outcomes, except for suicide, at the longest follow-up within 12 months. Studies have suggested that a short follow-up duration (less than 12 months) after the intervention may not be enough to change the prevalence of suicide [28]. Thus, we measured the outcome of suicide at the longest follow-up within 24 months. In the before-and-after comparison design, we measured all of the above outcomes in the control group during the period immediately preceding the intervention. Regarding depressive symptom severity, we accepted the following assessment instruments for the measurement of depressive symptoms: (1) Patient Health Questionnaire-9 [29], (2) Beck's Depression Inventory [30], or other validated scales. Regarding anxiety symptom severity, we accepted the following assessment instruments for measuring anxiety symptoms: (1) Generalized Anxiety Disorder-7 [31], (2) Brief Fear of Negative Evaluation Scale [32], or other validated scales. Regarding PTSD symptom severity, we accepted the following assessment instruments for measuring PTSD symptoms: (1) Impact of Event Scale-Revised [33], (2) Impact of Event Scale [34], or other validated scales. Regarding insomnia symptom severity, we accepted the following clinical diagnostic instruments for measuring insomnia symptoms: (1) Pittsburgh Sleep Quality Index [35], (2) Insomnia Severity Index [36], or other validated scales. Regarding substance use, we measured substance use by the difference in the amount of substance use before and after the intervention. We focused on the following substances: (1) alcohol, (2) tobacco, (3) vape, and (4) cannabis. When multiple instruments measured the same outcome in the included studies, we selected the one that came first in our list.

Information Sources and Search Strategy

The following electronic databases were searched on April 11, 2022: (1) CENTRAL; (2) Medline Ovid; (3) PsycINFO Ovid; (4) US National Institutes of Health Ongoing Trials Register (ClinicalTrials.gov); and (5) WHO ICTRP. We describe the full search strategies in Supplementary material 2. We checked the reference lists of all included studies and relevant systematic reviews to identify additional studies missed during the original electronic searches. We also run a cited reference search in citation indexes of the Web of Science and Google Scholar. We contacted the authors of identified studies and asked them to identify other relevant published and unpublished studies. We conducted a literature search to identify all published and unpublished RCTs and NRSIs, including quasi-RCTs, non-randomized cross-over studies, prospective cohort studies, retrospective cohort studies, controlled before and after studies, interrupted time series (with comparison group), and cross-sectional studies in all languages. We translated non-English articles and thoroughly assessed them for potential inclusion as necessary.

Selection Process, Data Collection Process, and Data Items

Two pairs of four review authors (YO, KY, NS, TA) independently screened the titles and abstracts of search results, and they reviewed the full texts independently and identified studies for inclusion. Disagreements were resolved by discussion or by consulting other review authors (YT, ST, MB, YK). We recorded the reasons for the exclusion of ineligible full-text studies. We excluded all duplicate publications. When we found multiple reports/publications of the same study, we checked them to ensure that each study, rather than each report/publication, was the unit of interest in the review. Two pairs of seven review authors (YO, NS, TA, YT, ST, MB, YK) extracted study characteristics and outcome data from the included studies. They entered this information into a data collection form that we had specifically piloted for this review. Two pairs of eight review authors (YO, KY, NS, TA, YT, ST, MB, YK) transferred data to the Review Manager [37] and recorded the following data for the included studies. We also extracted the results regarding the following variables. For continuous outcomes (depressive symptom severity, anxiety symptom severity, PTSD symptom severity, insomnia symptom severity, and substance use), we extracted the mean value and standard deviation (SD) of the outcome of interest before and after the intervention. For dichotomous outcomes (suicide), we extracted the number of participants in each intervention arm who had the measured outcome of interest and the number of participants assessed at the endpoint. While we had planned to extract both crude odds ratios (ORs) and adjusted ORs at the endpoint, we did not identify any studies that reported these effect sizes.

Study Risk of Bias Assessment

Two pairs of seven review authors (YO, NS, TA, YT, ST, MB, YK) independently assessed the risk of bias for each outcome of interest in this review. Disagreements were resolved by discussion or by consulting other review authors (YT, ST, MB, YK). We were interested in assessing the risk of bias for the effect of assignment to the intervention. To assess the risk of bias in NRSIs, we used the "Risk Of Bias In Non-randomized Studies - of Interventions" (ROBINS-I) tool [38]. We judged each potential source of bias as having "low," "moderate," "serious," or "critical" bias or "no information." We summarized the risk of bias judgments across different studies for each of the domains listed and provided an overall risk of bias judgment in the "risk of bias" table.

Effect Measures and Synthesis Methods

We conducted a statistical analysis using the Review Manager [37]. We conducted a meta-analysis using a random-effects model if clinically similar studies were available to ensure meaningful conclusions. We excluded studies at an overall critical risk of bias from the meta-analysis based on the recommendation of ROBINS-I [38]. For continuous data, when studies used the same continuous outcome measure for comparison, we pooled data by using an adjusted mean difference (MD). We used the standardized mean difference (SMD) to pool data from studies that measure the same outcomes of interest using different methods. We presented 95% confidence intervals (CIs) and point estimates. For suicide, we conducted a narrative synthesis due to methodological heterogeneity because we had planned to conduct a meta-analysis only when pooling to make sense and based on the recommendation of ROBINS-I [38]. We displayed the results of studies that reported that no events occurred for an outcome in the forest plot; however, it did not contribute to the effect size.

Unit of Analysis Issues

The unit of analysis was individual participants included in the studies. For included studies that should consider individuals as cluster factors, we assessed whether the clustering effect had been dealt with effectively in the analysis of the included studies. When the original authors used a statistical model such as a mixed-effects model or generalized estimating equations to take the clustering effects into account, we chose it as the estimate for the synthesis. We critically appraised the cluster-level confounding through the confounding domain of the ROBINS-I tool [38]. For studies with multiple comparisons, we included all intervention groups that were assessed to be relevant to this review as per our pre-defined eligibility criteria.

Dealing With Missing Data

We contacted authors to verify key study characteristics and obtained missing numerical outcome data. However, we were unable to obtain the data from all the authors that we contacted. We used the median as the mean and calculated the SD from the standard error, interquartile range, or p-values, according to the Cochrane Handbook for Systematic Reviews of Interventions [39], and when we were unable to calculate the SD in this way, we imputed the SD as the median SD in the remaining studies included in the outcome. We assessed the impact of included studies with missing data in a sensitivity analysis. We excluded studies from which we could not obtain and impute sufficient data to synthesize, based on the criterion of "no appropriate data."

Reporting Bias Assessment

We aimed to assess the possibility of publication bias through funnel plot analysis; however, this was not feasible, as fewer than 10 studies satisfied this review's inclusion criteria. Therefore, we assessed reporting bias as publication bias based on unpublished studies in clinical trial registries and published protocols that were identified by a full-text screening.

Investigation of Heterogeneity and Subgroup Analysis

We conducted a univariate meta-regression analysis to explore the relationship between the critical outcome (i.e., depressive symptom severity) and the potential sources of heterogeneity. Potential sources of clinical heterogeneity examined included age groups (i.e., adolescents (aged 10 years and older) versus adults (aged 20 years and older)) and country income levels (i.e., high-income countries versus low- or middle-income countries). For methodological heterogeneity, the presence or absence of adjustment for confounding factors (i.e., occupation, economic status, and relationships) was considered. We conducted the analysis using the metafor package, Version 4.4-0, in R software, Version 4.3.2 (R Foundation for Statistical Computing, Vienna, Austria, https://www.R-project.org/). We tabulated the results from the meta-regression analysis. We evaluated the assumptions of the meta-regression using residual plots, Cook’s distance, and visual inspection of moderator linearity. Due to the limited number of included studies and the lack of sufficient information on government lockdowns, we were unable to perform the meta-regression analysis for suicide outcomes or for depressive symptom severity based on the following potential sources of heterogeneity: the duration of government lockdowns, enforcement of lockdowns with penalties for non-compliance, provision of public subsidies for wages during lockdowns, methods of allocation to study groups, and overall risk of bias. We also assessed statistical heterogeneity by calculating the I^2^.

Sensitivity Analysis

We conducted a sensitivity analysis for the critical outcomes by excluding studies with imputed data. Due to the limited number of studies included and the lack of information, we could not conduct sensitivity analysis for suicide and for depressive symptom severity under the condition that studies classified as having an overall serious risk of bias were excluded. In addition, we could not conduct a responder analysis for both outcomes.

Certainty of the Evidence Assessment

We summarized the body of evidence for critical outcomes using the Grading of Recommendations Assessment, Development, and Evaluation (GRADE) system [40]. Using the GRADEpro software, we created a "summary of findings" table for summarizing outcome-specific information. To inform the GRADE levels of evidence, we used the overall risk of bias evaluated by ROBINS-I because we identified only NRSIs in our review.

Results

**Figure 1:**
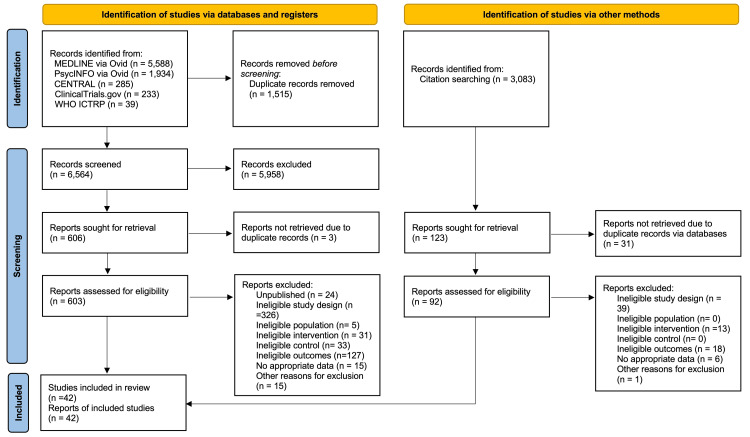
PRISMA flow diagram

A total of 8,079 records were identified from the electronic database search, and 3,083 records were identified from the citation search. We retrieved full texts of 603 reports from the electronic database and 92 reports from citation searches for full assessment. Of these 695 full-text articles, we included 42 studies in our review (Figure 1). We excluded 653 reports (Supplementary material 3) and identified 24 ongoing studies (Supplementary material 4).

Characteristics of Included Studies

Of the 42 studies that met our review criteria, all the included studies were NRSIs (Table 1; Supplementary material 5) [41-82].

**Table 1:** Characteristics of the included studies *We classified levels of country income according to the World Bank’s 2020 classification.

Study (year)	Country^*^	Methods (design, key design feature)	Participants	Interventions	Outcomes
Acharya et al. (2022) [41]	Nepal (low or middle income)	Retrospective (time differences)	General population	Lockdown	Suicide
Albrecht et al. (2022) [42]	Switzerland (high income)	Cross-sectional surveys (time differences)	12,238 high school students	Lockdown	Substance use (tobacco, alcohol)
Arad et al. (2021) [43]	Israel (high income)	Longitudinal (time differences)	99 undergraduate freshmen	COVID-19 lockdown	Anxiety symptoms
Barbosa et al. (2021) [44]	United States (high income)	Cross-sectional (time differences)	556 adults	Stay-at-home orders	Alcohol use
Bartlett et al. (2021) [45]	Australia (high income)	Longitudinal (time differences)	1,671 adults (50+ years)	Lockdown restrictions	Depressive symptoms, anxiety symptoms, alcohol use
Bennett et al. (2022) [46]	United Kingdom (high income)	Longitudinal (time differences)	6,330 university students	National lockdown	Depressive symptoms, anxiety symptoms
Berthelot et al. (2020) [47]	Canada (high income)	Longitudinal (time differences)	2,078 pregnant women	Public health emergency	Post-traumatic stress disorder symptoms
Boekhorst et al. (2021) [48]	Netherlands (high income)	Longitudinal (time differences)	669 pregnant women	Nationwide lockdown	Depressive symptoms
Bouter et al. (2023) [49]	Netherlands (high income)	Longitudinal (time differences)	445 adolescents	Lockdown	Depressive symptoms, anxiety symptoms
Burdzovic Andreas and Brunborg (2022) [50]	Norway (high income)	Longitudinal (time differences)	2,536 adolescents	Nationwide lockdown	Alcohol use
Cellini et al. (2021) [51]	Italy and Belgium (high income)	Cross-sectional (time differences)	1,622 (Italy) and 650 (Belgium) adults	Lockdown	Insomnia symptoms
Cellini et al. (2021) [52]	Italy (high income)	Cross-sectional (time differences)	299 mothers	National lockdown	Insomnia symptoms
Cody et al. (2021) [53]	Switzerland (high income)	Cross-sectional (time differences)	165 individuals with depression	COVID-19 lockdown	Insomnia symptoms
Cohen et al. (2021) [54]	Netherlands (high income)	Cohort (time differences)	535 patients with hand/wrist conditions	Intelligent lockdown	Depressive symptoms, anxiety symptoms
Cousijn et al. (2021) [55]	Netherlands (high income)	Cross-sectional	120 cannabis users	Dutch lockdown	Cannabis use
Dunn et al. (2021) [56]	United States (high income)	Longitudinal (time differences)	48 adults with cochlear implant	State of public health disaster emergency	Depressive symptoms, anxiety symptoms
Gonzalez-Martinez et al. (2021) [57]	Spain (high income)	Longitudinal (time differences)	158 patients with epilepsy	Lockdown	Anxiety symptoms, Insomnia symptoms
Hausman et al. (2022) [58]	United States (high income)	Longitudinal (time differences)	189 older adults	Stay-at-home orders	Depressive symptoms, anxiety symptoms, insomnia symptoms
Kekäläinen et al. (2021) [59]	Finland (high income)	Longitudinal (time differences)	358 women (47-55 years)	Lockdown	Depressive symptoms, alcohol use
Koenders et al. (2021) [60]	Netherlands (high income)	Ecological (time differences)	36 patients with bipolar disorder	Lockdown	Depressive symptoms
Koenig et al. (2023) [61]	Germany (high income)	Longitudinal (time differences)	324 children and adolescents (≥12 years)	Lockdown	Depressive symptoms
Leatherdale et al. (2023) [62]	Canada (high income)	Longitudinal (time differences)	7,653 adolescents	Lockdown	Vaping
Lee et al. (2020) [63]	United States (high income)	Longitudinal (time differences)	546 young adults	Mitigation policies	Depressive symptoms, anxiety symptoms
Leightley et al. (2021) [64]	United Kingdom, Spain, Netherlands (high income)	Longitudinal (time differences)	252 individuals with MDD	Lockdown	Depressive symptoms
Liu et al. (2022) [65]	United States (high income)	Longitudinal (time differences)	175 adolescents	Stay-at-home orders	Depressive symptoms
Macfarlane et al. (2021) [66]	United Kingdom (high income)	Re-surveyed cohorts (time differences)	1,054 individuals with musculoskeletal disease	National lockdown	Depressive symptoms, anxiety symptoms, insomnia symptoms
Mauz et al. (2023) [67]	Germany (high income)	Longitudinal (time differences)	26,152 adults (18+)	Lockdown	Depressive symptoms
Meda et al. (2021) [68]	Italy (high income)	Longitudinal (time differences)	358 university students	COVID-19 lockdown	Depressive symptoms, anxiety symptoms
Minhas et al. (2021) [69]	Canada (high income)	Longitudinal (time differences)	473 emerging adults	Lockdown	Depressive symptoms, anxiety symptoms, alcohol use
Moya et al. (2021) [70]	Colombia (low or middle income)	Longitudinal (time differences)	1,376 primary caregivers	National lockdown	Depressive symptoms, anxiety symptoms
Murphy et al. (2023) [71]	United States (high income)	Longitudinal (time differences)	204 individuals from three generations	Lockdown and social distancing	Depressive symptoms, anxiety symptoms
Overbeck et al. (2021) [72]	Denmark (high income)	Cross-sectional (time differences)	1,758 pregnant women	COVID-19 lockdown	Depressive symptoms, anxiety symptoms
Pelham et al. (2022) [73]	United States (high income)	Longitudinal (time differences)	494 adolescents (12-21 years)	Stay-at-home orders	Alcohol use, tobacco use
Rimfeld et al. (2022) [74]	England and Wales (high income)	Longitudinal (time differences)	4,773 individuals	Lockdown	Depressive symptoms, anxiety symptoms
Romdhani et al. (2022) [75]	49 countries	Cross-sectional (time differences)	3,911 athletes	Lockdown	Insomnia symptoms
Sacre et al. (2021) [76]	Australia (high income)	Cross-sectional (time differences)	450 adults with type 2 diabetes	Lockdown	Depressive symptoms, anxiety symptoms
Shoshani et al. (2021) [77]	Israel (high income)	Cross-sectional (time differences)	1,537 students (5th-11th grade)	Lockdown	Depressive symptoms, anxiety symptoms
Tanaka et al. (2021) [78]	Japan (high income)	Longitudinal (time differences)	General population	State of emergency	Suicide
van der Velden et al. (2022) [79]	Netherlands (high income)	Population-based (time differences)	740 adults	Lockdown	Depressive symptoms, Post-traumatic stress symptoms
van den Besselaar et al. (2021) [80]	Netherlands	Longitudinal (time differences)	1,128 older adults	Social distancing measures	Depressive symptoms, anxiety symptoms
Yang et al. (2021) [81]	China (low or middle income)	Longitudinal (time differences)	195 college students	Lockdown	Depressive symptoms
Zijlmans et al. (2023) [82]	Netherlands (high income)	Longitudinal (time differences)	2,401 children (8-18 years)	Lockdown	Depressive symptoms, anxiety symptoms, insomnia symptoms

All the included studies assessed the impact of the COVID-19 pandemic on mental health outcomes. Key study design features (i.e., how groups of individuals or clusters were formed) in all included studies were time differences. Among the 42 studies, we found that the study participants were from the general population in 27 studies [41-46,49-52,54,55,58,59,62,63,65,67-70,73,77,78,80-82]. Interventions were explicitly defined as "lockdown" in 33 studies [41-43,45,46,48-55,57,59-62,64,66-72,74-77,79,81,82], and as "stay-at-home order" in four studies [44,58,65,73]. We identified 21 studies that reported government lockdown periods exceeding one month [43,45-51,53,56,59,60,62,64-66,68,72,76,80,82]. A total of 27 studies reported on depressive symptom severity [45,46,48,49,54,56,58-61,63-72,74,76,77,79-82], 2 on suicide [41,78], 20 on anxiety symptom severity [43,45,46,49,50,56-58,63,66,68-72,74,76,77,79,82], 2 on PTSD symptom severity [47,79], 8 on insomnia symptom severity [51-53,57,58,66,75,82], and 9 on substance use [42,44,45,50,55,59,62,69,73].

Excluded Studies

We excluded a total of 653 full-text studies. For the electronic database search, we excluded 576 studies due to the following reasons: unpublished (N = 24), ineligible study design (N = 326), ineligible population (N = 5), ineligible intervention (N = 31), ineligible control (N = 33), ineligible outcomes (N = 127), no appropriate data (N = 15), and other reasons (e.g., narrative review) (N = 15). For the citation research, we excluded 77 studies due to ineligible design (N = 39), ineligible intervention (N = 13), ineligible outcomes (N = 18), no appropriate data (N = 6), and other reasons (N = 1). We describe the reasons for exclusions and the characteristics of 629 excluded studies in Supplementary material 3.

Risk of Bias Assessment for Critical Outcomes

**Figure 2:**
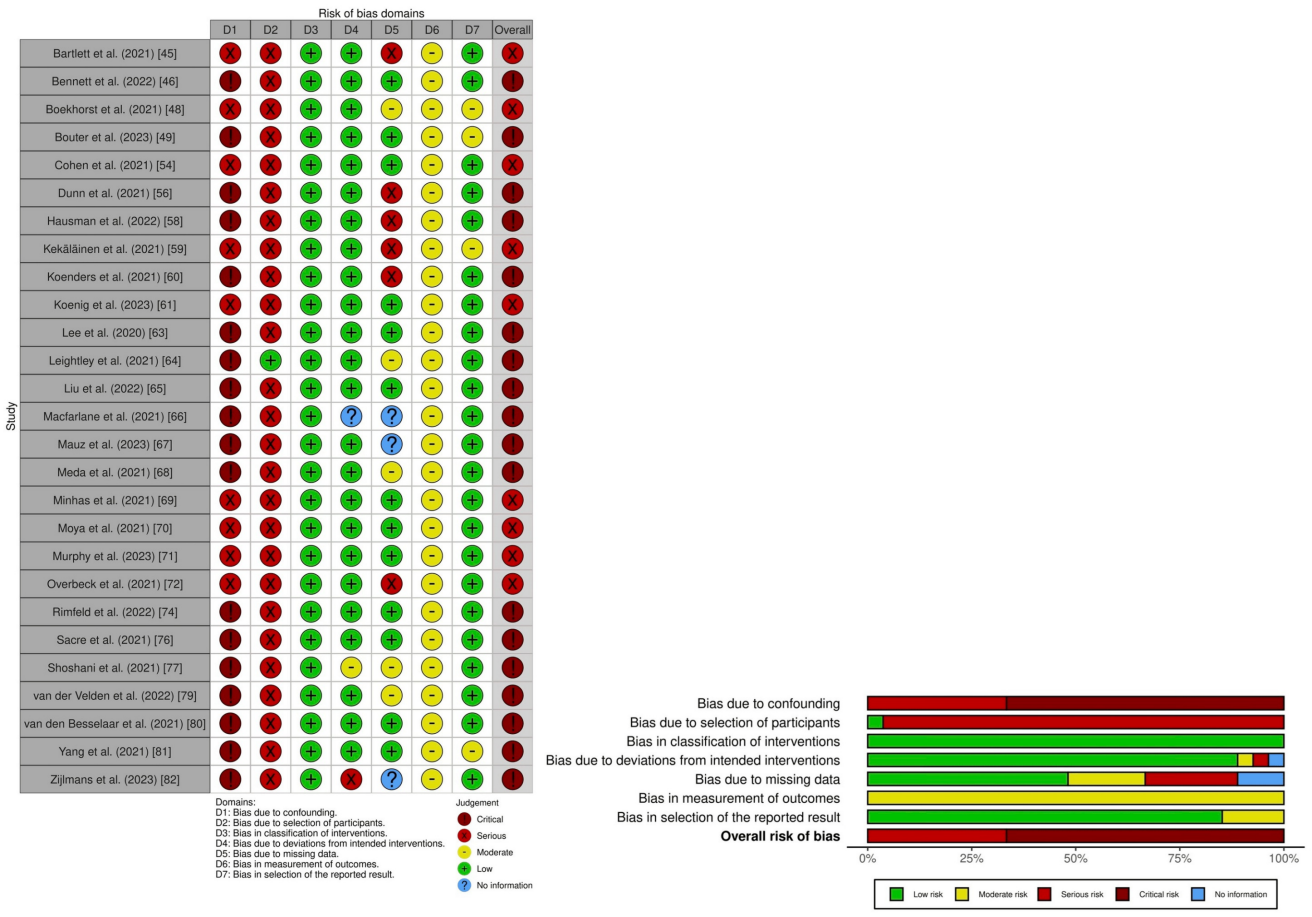
Risk of bias assessment using the ROBINS-I for depressive symptom severity

We summarize the risk of bias for depressive symptom severity in Figure 2, and a detailed risk of bias assessment is provided in Supplementary material 6.

**Figure 3:**
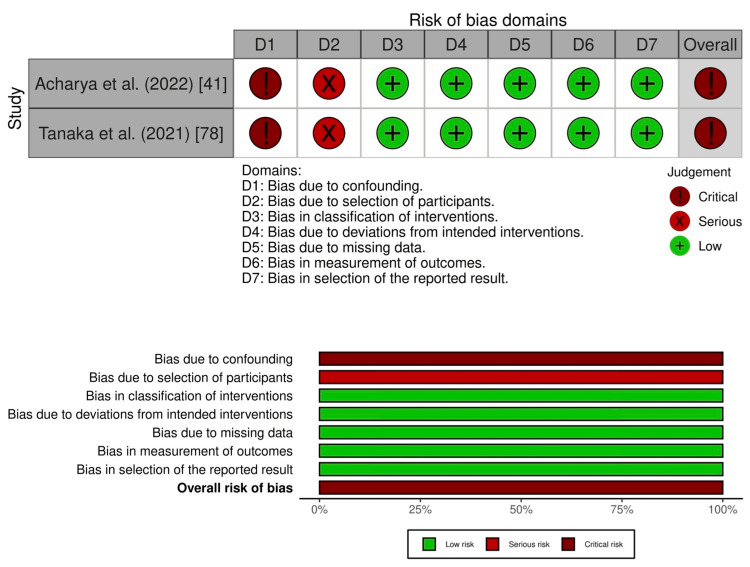
Risk of bias assessment using the ROBINS-I for suicide

Nine studies had an overall serious risk of bias [45,48,55,59,61,69-72], and 18 studies had an overall critical risk of bias [46,49,56,58,60,63-68,74,76,77,79-82]. For suicide, two studies had an overall critical risk of bias (Figure 3; Supplementary material 7) [41,78].

Risk of Bias Assessment for Important Outcomes

For anxiety symptom severity, six studies had an overall serious risk of bias [45,54,69-72], while 14 studies had an overall critical risk of bias (Supplementary materials 8 and 9) [43,46,49,56-58,63,66,68,74,76,77,79,82]. For PTSD symptom severity, one study had an overall serious risk of bias [47], while other studies had an overall critical risk of bias (Supplementary materials 10 and 11) [79]. For insomnia symptom severity, two studies had an overall serious risk of bias [51,52], and six studies had an overall critical risk of bias (Supplementary materials 12 and 13) [53,57,58,66,75,82]. For substance use, five studies had an overall serious risk of bias [44,45,50,59,69], and four studies had an overall critical risk of bias (Supplementary materials 14 and 15) [42,55,62,73].

Synthesis of Critical Outcomes

**Figure 4:**
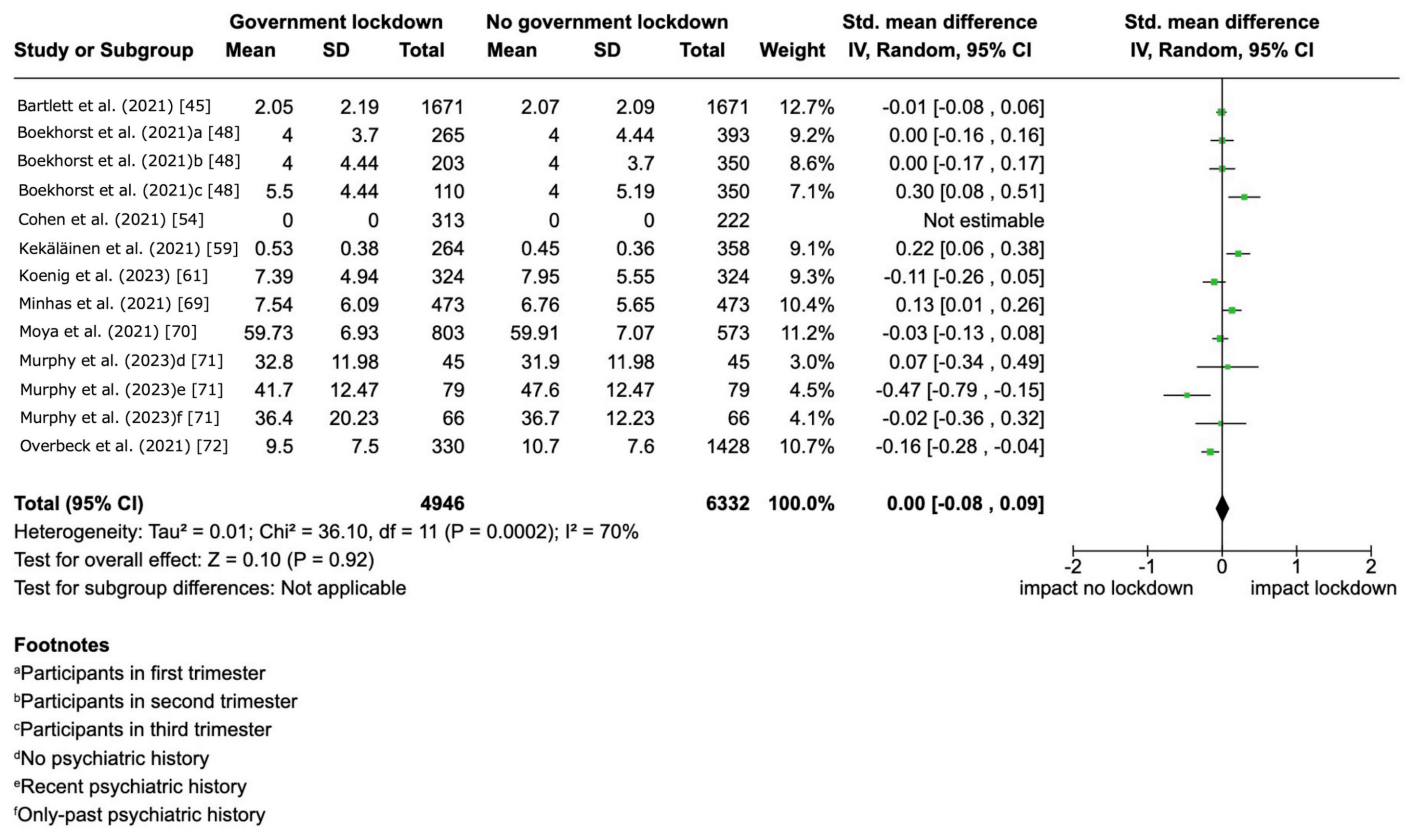
Government lockdown versus no government lockdown during the COVID-19 pandemic: depressive symptom severity We pooled the standardized mean difference (SMD) in eight studies. The study by Cohen et al. (2021) [54] was not included in the meta-analysis because the SMD could not be estimated due to zero scores in both the intervention and control groups.

Among the 27 included studies that reported depressive symptom severity, 18 studies were excluded from the meta-analysis due to an overall critical risk of bias as per our protocol [26]. Government lockdowns may have little to no effect on depressive symptom severity within the 12-month follow-up, but the evidence was very uncertain (SMD 0.00, 95% CI -0.08 to 0.09, I^2^ = 70%; 8 studies, 10,743 participants) (Figure 4; Table 2).

**Table 2:** Summary of the findings table: impact of government lockdown versus no government lockdown on mental health during the COVID-19 outbreak Patient or population: the general population during the COVID-19 pandemic. Setting: COVID-19 outbreak. Intervention: government lockdown. Comparison: no government lockdown. *The risk in the intervention group (and its 95% confidence interval) is based on the assumed risk in the comparison group and the relative effect of the intervention (and its 95% CI). CI: confidence interval, SMD: standardized mean difference. ^a^We downgraded the evidence by one level due to clinical and statistical heterogeneities. The follow-up periods in the included studies varied: in five studies, the latest follow-up was within two months post-government lockdown; in three studies, it was within six months; and in one study, it was between 6 and 12 months. The effects of the government lockdown were inconsistent, as indicated by an I^2^ statistics of 70%. ^b^We downgraded the evidence by one level because 5.5% (15 out of 272) of the studies reporting this outcome registered in clinical trial registries were unpublished. ^c^We downgraded the evidence by two levels because the overall risk of bias in the two included studies was critical. ^d^We downgraded the evidence by one level due to clinical heterogeneity. In one study, the follow-up period was 15 months post-government lockdown, while in another study, it ranged from 3 to 6 months after the lockdown. ^e^We downgraded the evidence by one level because the effect size of the intervention could not be synthesized, as the data were not amenable to pooling.

Outcomes	Anticipated absolute effects^*^ (95% CI)		Relative effect (95% CI)	No of participants (studies)	Certainty of the evidence (GRADE)	Comments
	Risks with no government lockdown	Risks with government lockdown				
Depressive symptom severity	-	SMD 0 SD (0.08 lower to 0.09 higher)	-	10,743 (8 non-randomized studies)	⊕⊝⊝⊝ Very low^a,b^	The evidence about the effect of government lockdown on depressive symptoms is very uncertain.
Suicide	Acharya et al. (2022) [41] reported that risk with no government lockdown was 2.06 per 100,000; risk with government lockdown was 2.43 per 100,000; incidence rate ratio (IRR) was 1.33 (95% CI 1.2-1.48) (follow-up: 15 months). Tanaka et al. (2021) [78] reported that risk with no government lockdown was 1.28 per 100,000; risk with government lockdown was 1.46 per 100,000; IRR was 1.16 (95% CI 1.11-1.21) (follow-up: 3-6 months).		-	Two non-randomized studies	⊕⊝⊝⊝ Very low^c,d,e^	The evidence about the effect of government lockdown on suicide rates is very uncertain. We conducted a narrative synthesis because the overall risk of bias in the two included studies, assessed by ROBINS-I, was critical.

Table 3 shows the result from a univariate meta-regression analysis assessing the heterogeneity based on age groups, country income levels, and adjustment for confounding factors (i.e., occupation, economic status, and relationship status).

**Table 3:** Investigation of potential sources of heterogeneity for depressive symptom severity using a univariate meta-regression analysis CI: confidence interval, SMD: standardized mean difference. *The study by Cohen et al. (2021) [54] was not included in the meta-regression analysis because the SMD was not estimated. ^**^Reference: high-income countries. ‡Reference: adolescents.

Potential sources	Included study (n = 8)*	Estimated SMD (95% CI)
Clinical heterogeneity		
Age group^‡^	Adolescent (n = 1)	0.12 (-0.20 to 0.45)
Country income level^**^	High-income countries (n = 7)	-0.04 (-0.36 to 0.29)
Methodological heterogeneity (the presence or absence of adjustment for confounding factors)		
Occupation	Adjusted (n = 4)	0.10 (-0.10 to 0.29)
Economic status	Adjusted (n = 3)	0.00 (-0.22 to 0.21)
Relationship status	Adjusted (n = 5)	0.00 (-0.21 to 0.22)

**Figure 5:**
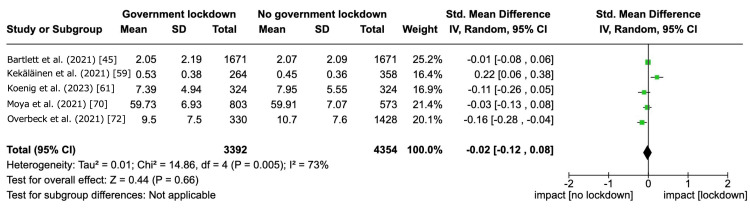
Sensitivity analysis. Government lockdown versus no government lockdown during the COVID-19 pandemic: depressive symptom severity

For the clinical heterogeneity, the estimated SMD was as follows: 0.12 (95% CI -0.20 to 0.45) for age groups and -0.04 (95% CI -0.36 to 0.29) for country income levels. For the methodological heterogeneity, the estimated SMD was as follows: 0.10 (95% CI -0.10 to 0.29) for occupation, 0.00 (95% CI -0.22 to 0.21) for economic status, and 0.00 (95% -0.21 to 0.22) for relationship status. For the sensitivity analysis by excluding studies with imputed data, we pooled SMD of five studies [45,59,61,70,72]. Government lockdowns may have little to no effect on depressive symptom severity within the 12-month follow-up (SMD -0.02, 95% CI -0.12 to -0.08, I^2^ = 73%; 5 studies, 7,746 participants) (Figure 5). These findings were consistent with the result of the main analysis.

Regarding suicide, we presented a narrative synthesis of two studies as a summary of findings in Table 2. Acharya et al. showed that government lockdowns may have a negative impact on suicide at 15-month follow-up (incidence rate ratio (IRR) 1.33, 95% CI 1.2-1.48) [41]. Tanaka et al. showed that government lockdowns may also have a negative impact at 3- to 6-month follow-up (IRR 1.16, 95% CI 1.11-1.21) [78]. However, these two studies had an overall critical risk of bias, and we judged the evidence on this outcome as very low certainty.

Synthesis of Important Outcomes

Among the 20 included studies that reported anxiety symptom severity, we pooled SMD in only five studies, except for exclusion, due to an overall critical risk of bias [45,69-72]. The study by Cohen et al. was not included in the meta-analysis because the SMD could not be estimated due to zero scores in both the intervention and control groups [54]. Government lockdowns may have little to no effect on anxiety symptom severity within the 12-month follow-up (SMD 0.08, 95% CI -0.10 to 0.26; I^2^ = 91%; 5 studies, 10,471 participants) (Supplementary material 16A), but the evidence was very uncertain.

Two studies reported PTSD symptom severity [47,79]. Berthelot et al. showed that government lockdowns may increase the outcome within the 12-month follow-up (MD 0.18, 95% CI 0.08-0.28; 1 study, 1,754 participants) (Supplementary material 16B), but the evidence was very uncertain [47].

Among the eight included studies that reported insomnia symptom severity, we pooled MD in two studies [51,52]. Government lockdowns may increase the outcome within the 12-month follow-up (MD 1.28, 95% CI 0.62-1.94; I^2^ = 91%; 2 studies, 5,142 participants) (Supplementary material 16C), but the evidence was very uncertain.

Among the nine included studies that reported on the amount of substance use, we pooled SMD in five studies that reported on alcohol [44,45,50,59,69]. Government lockdowns may have little to no effect on the outcome within the 12-month follow-up (SMD 0.03, 95% CI -0.05 to 0.11; I^2^ = 66%; 5 studies, 8,261 participants) (Supplementary material 16D), but the evidence was very uncertain. We considered two studies that reported on tobacco [42,73]: Leatherdale et al. who reported on vape use [62] and Cousijn et al. who reported on cannabis as having an overall critical risk of bias [55]. Therefore, these outcomes were not pooled in our review due to the limited number of studies.

Reporting Biases

We identified 24 ongoing studies (Supplementary material 4). In addition, we found two published protocols that were identified by a full-text screening [83,84]. However, one protocol was not published as an original article [84].

Discussion

This review assessed the impact of government lockdowns on mental health in the general population during outbreaks of emerging infectious diseases. Only studies during the COVID-19 outbreak met the inclusion criteria in our review. Overall, we found no evidence of the impact of government lockdown on mental health during the outbreak of emerging infectious diseases. Government lockdowns during the COVID-19 outbreak may have little to no effect on the depressive symptom severity in the general population; however, this evidence is very uncertain. In addition, the evidence about the impact of government lockdowns on suicide was very uncertain. The evidence about the impact on important outcomes was also very uncertain.

Interpreting the results of this review requires considering the follow-up period following the initiation of a government lockdown. Multiple lockdowns were implemented in various countries during the COVID-19 pandemic, and we did not examine the cumulative impact (i.e., the intensity of lockdowns) in this review. Individuals who experienced multiple lockdowns may have been more affected in terms of mental health; however, it is also possible that the acute stress response to lockdowns and the pandemic may have diminished over time, leading to a reduction in mental health impacts. In this review, among the pooled studies that reported depressive symptom severity, four studies [45,48,70,72] had follow-up periods of up to two months, and four studies [54,59,61,69] had follow-up periods ranging from two to six months, suggesting that the pooled effect size may predominantly reflect outcomes within six months from the start of lockdowns.

Two existing systematic reviews addressed a similar scope during the COVID-19 outbreak [85,86]. One review reported that the impact on mental health was not negligible [85], while another reported an initial increase in the average symptoms of depression and anxiety after the lockdown [86]. Potential reasons for the discrepancies may be attributed to differences in the included studies and the varying timing of outcome measurements after the lockdown. While this review only incorporated studies that adjusted for confounders affecting outcomes to mitigate the risk of bias inherent in unadjusted studies, the other two reviews were not restricted to such studies. Moreover, this review encompassed studies of the general population regardless of comorbidities. Concerning the timing of outcome measurements, one review encompassed only studies conducted between 1 and 60 days post-lockdown initiation [85]. Another review, which had the same observation period as ours, reported that the average symptoms of anxiety and depression worsened up to two months post-lockdown. However, the impact of the lockdown remained uncertain for periods extending beyond three months after the lockdown [86]. It is possible that the impact of government lockdowns on mental health may not elicit a uniform response among individuals after more than two months have passed since the initiation. This variability could depend on the magnitude of an individual's stress response and their specific circumstances.

Regarding emerging infectious diseases other than COVID-19, there was no evidence of the impact of government lockdowns. Non-pharmacological public health policy interventions are often implemented to control the spread of emerging infectious diseases, and lockdown is one such measure. However, despite extensive searches using various terms related to lockdown, there were no studies that investigated the mental health impacts of lockdown during outbreaks of SARS, MERS, H1N1, equine influenza, avian influenza (e.g., H7N9), or Ebola virus disease, leaving the effects of such interventions on mental health unclear. Therefore, we were not able to compare the effect of government lockdowns between emerging infectious diseases.

We could not find any studies that examined the impact of government lockdowns that mitigated the impact of the COVID-19 pandemic. In other words, we were unable to examine the impact of lockdown alone on mental health; during outbreaks of emerging infectious diseases such as COVID-19, where lockdown occurs at the time of spread, the effect may be due to both the lockdown and the pandemic [85]. An ideal study design would compare an area under government lockdown during an emerging infectious disease outbreak with another area without government lockdown but in a comparable epidemic state. While practically challenging, such a comparison would require not only confounding adjustments but also refined study design to truly examine the impact of government lockdowns.

The duration of the government lockdown was unclear in 20 studies, and the pre-specified co-interventions were not described in 41 studies. While this intensity and duration should be considered when examining the effects of lockdowns, many of the studies included in this review did not provide sufficient information. During an outbreak of emerging infectious diseases, it may be difficult to measure the effect of a uniform intervention due to the global spread of the disease and the different modalities of each lockdown in different countries.

The timing of measurement of outcomes in no government lockdown was varied. We defined the control group as the period immediately prior to the lockdown, while some included studies used several years prior to the lockdown as the control group. It is possible that local and global situations at this time (e.g., economic or political situations) may have affected the mental health of individuals, making it an inappropriate time for the control group. In that sense, comparisons between groups that underwent lockdowns and those that did not may be preferable as a design than comparative studies before and after lockdown (e.g., longitudinal studies).

The included studies in this review had an overall serious or critical risk of bias. Although 42 studies met our review criteria, only a limited number were adjusted for three pre-specified confounders. Using the ROBINS-I tool, we judged these studies to have an overall critical risk of bias when all pre-specified confounders were not adjusted. Consequently, we could not synthesize the effect sizes of these studies in a meta-analysis.

In our investigation of the heterogeneity of critical outcomes, we found inconsistent results across different assessments of heterogeneity. Although the meta-analysis shows substantial statistical heterogeneity regarding I^2^ among the included studies on depressive symptom severity, we could not identify the source of clinical and methodological heterogeneities by our meta-regression.

Several limitations should be considered when interpreting the findings of this review. First, we excluded the Embase database as part of our systematic literature search. Consequently, there is a risk that some relevant studies may have been missed. Nevertheless, the volume of research that required screening was substantial, and including Embase would have been impractical for the scope of this review. Second, we were unable to conduct all planned analyses, particularly subgroup and sensitivity analyses, due to insufficient data. In addition, it is important to note that for the subgroup analyses of depressive symptom severity, we had to exclude two studies due to a lack of sufficient data. Third, many studies were excluded due to "ineligible study design" in the before-and-after studies that did not adjust for confounding factors. Many studies reported the impact of government lockdowns on mental health during the COVID-19 outbreak, but we included and pooled data from studies that met a strict criterion in this review. Finally, we could not quantitatively evaluate reporting bias as per the protocol and instead had to consider the potential for publication bias qualitatively.

## Conclusions

Available evidence suggests that the impacts of government lockdowns on mental health in the general populations are very uncertain. While the social benefits of implementing government lockdowns to prevent the spread of emerging infectious diseases are evidently substantial, the potential adverse effects on mental health may not be taken into account. Future studies should employ rigorous research design, including comparisons between areas with and without government lockdowns during outbreaks, to provide stronger evidence. These studies should also consider the intensity of lockdowns, associated compensation measures, and potential confounders such as socioeconomic status, occupation, and relationships. Systematic reviews that include studies employing rigorous research designs with a low risk of bias will yield more trustworthy conclusions.

## Figures and Tables

**Figure 6:**
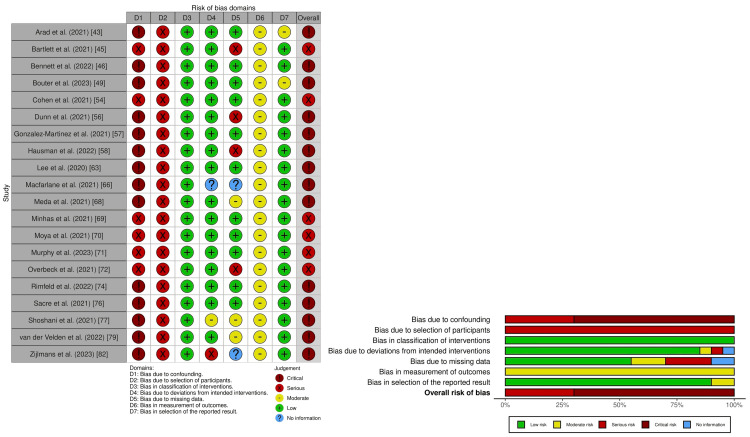
Risk of bias assessment using the ROBINS-I for anxiety symptom severity

**Figure 7:**
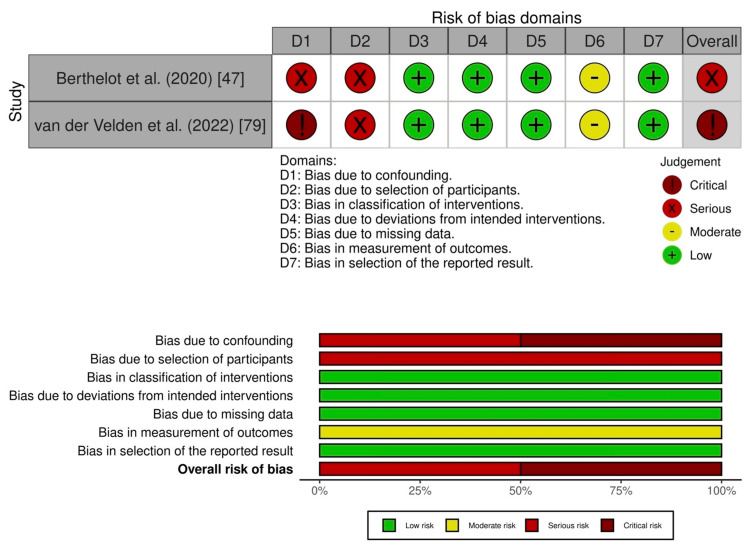
Risk of bias assessment using the ROBINS-I for post-traumatic stress disorder symptom severity

**Figure 8:**
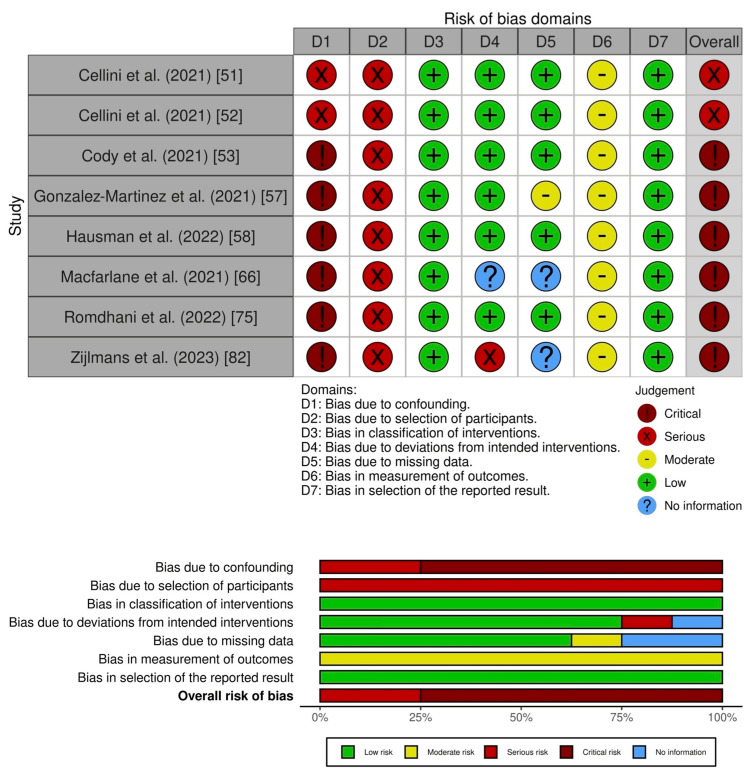
Risk of bias assessment using the ROBINS-I for insomnia symptom severity

**Figure 9:**
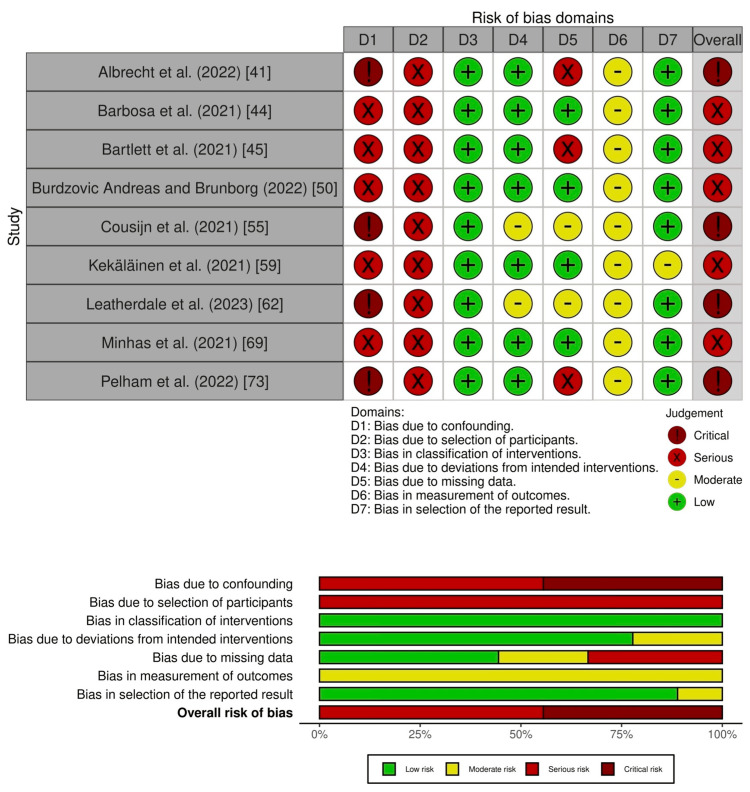
Risk of bias assessment using the ROBINS-I for substance use

**Figure 10:**
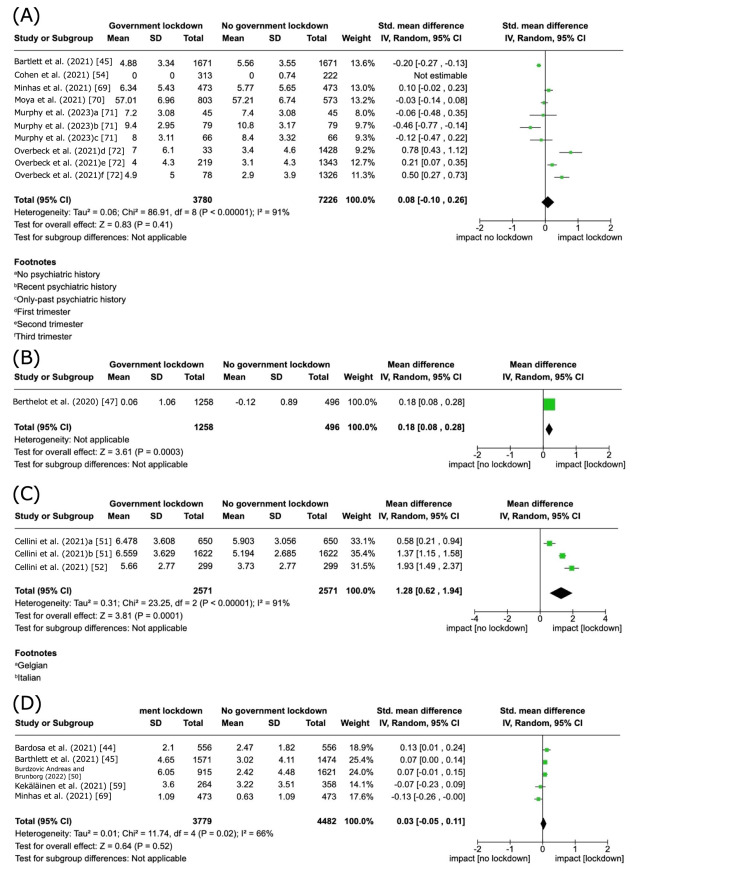
Government lockdown versus no government lockdown during the COVID-19 pandemic: important outcomes (A) Anxiety symptom severity. (B) Post-traumatic stress disorder symptom severity. (C) Insomnia symptom severity. (D) Substance use.

## Appendices

Methods

Study Settings and Designs

We used the following study design features to include studies in this review:

(1) The intervention/comparator was allocated to clusters or individuals, or clustered in the way in which it was provided (by practitioner or organization unit).

(2) Outcome data was available multiple times before and after the intervention (not necessarily for all the same individuals, as suicide was an outcome of interest).

(3) The intervention effect estimated the difference between groups (of individuals or clusters receiving either intervention or comparator).

(4) Methods for controlling confounding were used (for any confounding, for time-invariant unobserved confounding, or for confounding by observed covariates).

(5) Groups of individuals or clusters were formed by randomization, by quasi-randomization, or by other methods when randomization or quasi-randomization was not feasible (e.g., groups are divided by explicit rule for allocation based on a threshold for a variable measured on a continuous or ordinal scale or boundary, time differences, location differences, or the choice of policymakers).

(6) Assignment of individuals or clusters to intervention or control was determined after the study was designed. Choices leading to an individual or a cluster becoming a member of a group was made after the study was designed, or outcomes were assessed after the study was designed.

(7) Potential confounders were measured before the intervention, or outcomes variables were measured before the intervention.

Information Sources and Search Strategy

The Cochrane Common Mental Disorders' Information Specialist (SD) designed a preliminary search for Ovid Medline. This search strategy was then adapted by another Information Specialist (HF) for use on the following bibliographic databases using relevant subject headings (controlled vocabularies) and search syntax, appropriate to each resource. The results of the databases were deduplicated in EndNote 20.

Supplementary material 1

**Table 4:** Preferred Reporting Items for Systematic Review and Meta-Analysis Checklist 2020

Section and topic	Item #	Checklist item	Location where the item is reported
Title			
Title	1	Identify the report as a systematic review	#1
Abstract			
Abstract	2	See the PRISMA 2020 for Abstracts checklist	#1
Introduction			
Rationale	3	Describe the rationale for the review in the context of existing knowledge	#2
Objectives	4	Provide an explicit statement of the objective(s) or question(s) the review addresses	#2
Methods			
Eligibility criteria	5	Specify the inclusion and exclusion criteria for the review and how studies were grouped for the syntheses	#3,4
Information sources	6	Specify all databases, registers, websites, organisations, reference lists, and other sources searched or consulted to identify studies. Specify the date when each source was last searched or consulted	#4
Search strategy	7	Present the full search strategies for all databases, registers, and websites, including any filters and limits used	
Selection process	8	Specify the methods used to decide whether a study met the inclusion criteria of the review, including how many reviewers screened each record and each report retrieved, whether they worked independently, and if applicable, details of automation tools used in the process	#4
Data collection process	9	Specify the methods used to collect data from reports, including how many reviewers collected data from each report, whether they worked independently, any processes for obtaining or confirming data from study investigators, and if applicable, details of automation tools used in the process	#4
Data items	10a	List and define all outcomes for which data were sought. Specify whether all results that were compatible with each outcome domain in each study were sought (e.g., for all measures, time points, analyses), and if not, the methods used to decide which results to collect	#5
	10b	List and define all other variables for which data were sought (e.g., participant and intervention characteristics, funding sources). Describe any assumptions made about any missing or unclear information	#5
Study risk of bias assessment	11	Specify the methods used to assess risk of bias in the included studies, including details of the tool(s) used, how many reviewers assessed each study, and whether they worked independently, and if applicable, details of automation tools used in the process	#5
Effect measures	12	Specify for each outcome the effect measure(s) (e.g., risk ratio, mean difference) used in the synthesis or presentation of results	#5
Synthesis methods	13a	Describe the processes used to decide which studies were eligible for each synthesis (e.g., tabulating the study intervention characteristics and comparing against the planned groups for each synthesis (item #5))	#5
	13b	Describe any methods required to prepare the data for presentation or synthesis, such as handling of missing summary statistics, or data conversions	#5
	13c	Describe any methods used to tabulate or visually display results of individual studies and syntheses	#5
	13d	Describe any methods used to synthesize results and provide a rationale for the choice(s). If meta-analysis was performed, describe the model(s), method(s) to identify the presence and extent of statistical heterogeneity, and software package(s) used	#5
	13e	Describe any methods used to explore possible causes of heterogeneity among study results (e.g., subgroup analysis, meta-regression)	#5
	13f	Describe any sensitivity analyses conducted to assess robustness of the synthesized results	#6
Reporting bias assessment	14	Describe any methods used to assess risk of bias due to missing results in a synthesis (arising from reporting biases)	#5
Certainty assessment	15	Describe any methods used to assess certainty (or confidence) in the body of evidence for an outcome	#6
Results			
Study selection	16a	Describe the results of the search and selection process, from the number of records identified in the search to the number of studies included in the review, ideally using a flow diagram	#6,8
	16b	Cite studies that might appear to meet the inclusion criteria, but which were excluded, and explain why they were excluded	#9
Study characteristics	17	Cite each included study and present its characteristics	Table 1
Risk of bias in studies	18	Present assessments of risk of bias for each included study	#9,10
Results of individual studies	19	For all outcomes, present, for each study: (a) summary statistics for each group (where appropriate) and (b) an effect estimate and its precision (e.g., confidence/credible interval), ideally using structured tables or plots	Table 1
Results of syntheses	20a	For each synthesis, briefly summarise the characteristics and risk of bias among contributing studies.	Table2
	20b	Present results of all statistical syntheses conducted. If meta-analysis was done, present for each the summary estimate and its precision (e.g., confidence/credible interval) and measures of statistical heterogeneity. If comparing groups, describe the direction of the effect	#10
	20c	Present results of all investigations of possible causes of heterogeneity among study results	#12,13
	20d	Present results of all sensitivity analyses conducted to assess the robustness of the synthesized results	#13
Reporting biases	21	Present assessments of risk of bias due to missing results (arising from reporting biases) for each synthesis assessed	#14
Certainty of evidence	22	Present assessments of certainty (or confidence) in the body of evidence for each outcome assessed	Table 2
Discussion			
Discussion	23a	Provide a general interpretation of the results in the context of other evidence	#14
	23b	Discuss any limitations of the evidence included in the review	#15
	23c	Discuss any limitations of the review processes used	#15
	23d	Discuss implications of the results for practice, policy, and future research	#15
Other information			
Registration and protocol	24a	Provide registration information for the review, including register name and registration number, or state that the review was not registered	#2
	24b	Indicate where the review protocol can be accessed, or state that a protocol was not prepared	#2
	24c	Describe and explain any amendments to information provided at registration or in the protocol	#2
Support	25	Describe sources of financial or non-financial support for the review, and the role of the funders or sponsors in the review	#90
Competing interests	26	Declare any competing interests of review authors	#90
Availability of data, code, and other materials	27	Report which of the following are publicly available and where they can be found: template data collection forms; data extracted from included studies; data used for all analyses; analytic code; any other materials used in the review	Appendix Table

Supplementary material 2 

**Table 5:** Search strategy

Search strategy
MEDLINE(R) ALL via Ovid
1 COVID-19/ (152256)
2 Severe Acute Respiratory Syndrome/ (5679)
3 Ebolavirus/ (3744)
4 *Influenza, Human/ (46204)
5 *Communicable Disease Control/ (16545)
6 (COVID or COVID-19 or COVID19 or COVID2019).ti,ab. (207568)
7 (severe acute respiratory syndrome or SARS or SARS-Cov* or SARSCov* or nCoV or novel CoV or corona vir* or coronavir* or neocorona vir* or neocoronavir*).ti,ab. (136407)
8 (MERS or mers-cov or merscov* or middle east respiratory syndrome?).ti,ab. (7617)
9 ebola.ti,ab. (9727)
10 ((infect* or contagio*) adj disease?).ti,kw. and (pandemic* or epidemic*).ti,ab. (2163)
11 (flu adj1 pandemic?).ti,ab. (834)
12 or/1-11 (332204)
13 *Social Isolation/ (6730)
14 Physical Distancing/ (2017)
15 *Quarantine/ (2783)
16 Quarantine/px (858)
17 ((social* or physical*) adj (isolat* or distanc* or seclu*)).ti,ab. (22084)
18 (lockdown or lock* down*).ti,ab. (11905)
19 (self isolat* or self-isolat* or self quarantine* or self-quarantine*).ti,ab. (1152)
20 ((enforce* or impose* or mandatory or require* or national*) adj2 (quarantine* or shielding or curfew*)).ti,ab. (668)
21 (("stay at home" or stay-at-home or movement control) adj order?).ti,ab. (908)
22 ((restrict* or ban*3 or limit*) adj2 (movement? or travel* or social* or contact or freedom?)).ti,ab. (12744)
23 or/13-22 (52168)
24 12 and 23 (24246)
25 Mental Health/ (51895)
26 *Stress, Psychological/ (80823)
27 Anxiety Disorders/ (38386)
28 *Depression/ (83703)
29 Panic Disorder/ (7179)
30 Phobic Disorders/ (10993)
31 Self-injurious Behavior/ (9091)
32 (mental* adj (health* or ill* or well* or unwell)).ti,ab. (211250)
33 ((emotional* or psychological*) adj (impact* or stress* or distress* or trauma*)).ti,ab. (55370)
34 ((stress or mood or panic or sleep*) adj2 disorder?).ti,ab. (86003)
35 (depressi* or anxiety or GAD or agoraphobi* or phobia or insomni*).ti,ab. (577966)
36 (self* adj (injur* or harm* or mutilat*)).ti,ab. (13408)
37 Alcohol-Related Disorders/ (5602)
38 *Substance-Related Disorders/ (74787)
39 ((alcohol or drink* or tobacco* or smoking or cannabi* or substance* or drug*) adj2 (disorder* or addict* or abus* or dependen* or problem*)).ti,ab. (157303)
40 or/25-39 (1050507)
41 24 and 40 (5613)
42 exp animals/ not humans.sh. (4988163)
43 41 not 42 (5606)
44 remove duplicates from 43 (5588)
APA PsycInfo via Ovid
1 COVID-19/ (8357)
2 Severe Acute Respiratory Syndrome/ (300)
3 Influenza/ (1438)
4 Disease Outbreaks/ (250)
5 (COVID or COVID-19 or COVID19 or COVID2019).ti,ab. (14848)
6 (severe acute respiratory syndrome or SARS or SARS-Cov* or SARSCov* or nCoV or novel CoV or corona vir* or coronavir* or neocorona vir* or neocoronavir*).ti,ab. (4976)
7 (MERS or mers-cov or merscov* or middle east respiratory syndrome?).ti,ab. (172)
8 ebola.ti,ab. (514)
9 ((infect* or contagio*) adj disease?).ti,id. and (pandemic* or epidemic*).ti,ab. (237)
10 (flu adj1 pandemic?).ti,ab. (91)
11 or/1-10 (18276)
12 Social Isolation/ (8249)
13 Physical Distancing/ (664)
14 Quarantine/ (506)
15 ((social* or physical*) adj (isolat* or distanc* or seclu*)).ti,ab. (14158)
16 (lockdown or lock* down*).ti,ab. (1855)
17 (self isolat* or self-isolat* or self quarantine* or self-quarantine*).ti,ab. (308)
18 ((enforce* or impose* or mandatory or require* or national*) adj2 (quarantine* or shielding or curfew*)).ti,ab. (73)
19 (("stay at home" or stay-at-home or movement control) adj order?).ti,ab. (218)
20 ((restrict* or ban*3 or limit*) adj2 (movement? or travel* or social* or contact or freedom?)).ti,ab. (4723)
21 or/12-20 (25748)
22 11 and 21 (4056)
23 *Mental Health/ (57035)
24 Psychological Stress/ (9351)
25 Anxiety Disorders/ (19838)
26 *"Depression (Emotion)"/ (20175)
27 Panic Disorder/ (7850)
28 Phobias/ (5544)
29 Self-Injurious Behavior/ (5046)
30 (mental* adj (health* or ill* or well* or unwell)).ti,ab. (239273)
31 ((emotional* or psychological*) adj (impact* or stress* or distress* or trauma*)).ti,ab. (44007)
32 ((stress or mood or panic or sleep*) adj2 disorder?).ti,ab. (75949)
33 (depressi* or anxiety or GAD or agoraphobi* or phobia or insomni*).ti,ab. (447523)
34 (self* adj (injur* or harm* or mutilat*)).ti,ab. (13776)
35 Alcohol Abuse/ (18875)
36 "Substance Use Disorder"/ (9507)
37 ((alcohol or drink* or tobacco* or smoking or cannabi* or substance* or drug*) adj2 (disorder* or addict* or abus* or dependen* or problem*)).ti,ab. (123769)
38 or/23-37 (806542)
39 22 and 38 (1934)
40 remove duplicates from 39 (1934)
CENTRAL via the Cochrane Library
#1 (emerg* NEXT (infect* NEXT disease)):ti,ab,kw 16
#2 [mh ^"disease outbreaks"] 212
#3 ([mh ^"COVID-19"] or [mh ^coronavirus]) 1552
#4 [mh ^"Severe Acute Respiratory Syndrome"] 371
#5 (COVID or COVID-19 or COVID19 or COVID2019):ti,ab,kw 10076
#6 ("severe acute respiratory syndrome" or SARS or SARS NEXT Cov* or SARSCov* or nCoV or novel NEXT CoV or corona NEXT vir* or coronavir* or neocorona NEXT vir* or neocoronavir*):ti,ab,kw 7852
#7 (MERS or mers NEXT cov or merscov* or "middle east respiratory syndrome"):ti,ab,kw 166
#8 ([mh ^"Influenza, Human"] or [mh "Influenzavirus A"]) 3097
#9 (influenza or (flu NEAR/1 pandemic?)):ti,ab,kw 8085
#10 ((H1N1 or H2N2 or H3N2 or H5N1 or H5N8 or H7N3 or H7N9 or (avian or bird or equine or swine)) NEXT flu):ti,ab,kw 64
#11 ([mh ^"hemorrhagic fever, ebola"] or [mh "hemorrhagic fevers, viral"]) 513
#12 (Ebola or h*morrhagic NEXT fever*):ti,ab,kw 595
#13 {OR #1-#12} 19488
#14 (lockdown or lock* NEXT down*):ti,ab,kw 166
#15 [mh quarantine[mj]] 5
#16 ((epidemic? or pandemic* or global* or international or worldwide or world NEXT wide or national or regional or mass or population* or impose? or imposing or enforce* or force* or mandat* or voluntary or polic*) NEAR/5 (quarantin* or isolation)):ti,ab,kw 177
#17 quarantin*:ti 29
#18 (self NEXT isolat* or selfisolat* or shielding):ti,ab,kw 323
#19 "movement control order*":ti,ab,kw 3
#20 ((ban or bans or banned or restrict*) NEAR/2 (movement? or travel* or social* or contact? or interact*)):ti,ab,kw 511
#21 (border? NEAR/2 (closed or closure?)):ti,ab,kw 7
#22 (stay* NEAR/2 home?):ti,ab,kw 242
#23 ((work* or school*) NEAR/2 (home? or remote*)):ti,ab,kw 1187
#24 furlough*:ti,ab,kw 10
#25 (social* NEAR/3 (isolat* or distanc* or seclusion*)):ti,ab,kw 1643
#26 human NEXT contact?:ti,ab,kw 35
#27 (freedoms or libert* or civil NEXT right?):ti,ab,kw 659
#28 {OR #14-#27} 4764
#29 [mh ^"Mental Health"] 1867
#30 (mental* NEAR/2 (health* or ill* or well*)):ti,ab,kw 27827
#31 ([mh ^"Adaptation, Psychological"] or [mh ^"Stress, Psychological"]) 9751
#32 ((emotional* or psychological*) NEXT (adapt* or impact* or stress* or distress* or trauma*)):ti,ab,kw 7779
#33 ([mh ^"mental disorders"[mj]] or [mh ^"mood disorders"] or [mh ^depression] or [mh ^"depressive disorder"] or [mh ^"depression, postpartum"] or [mh ^"depressive disorder, major"] or [mh ^"depressive disorder, treatment-resistant"] or [mh ^"dysthymic disorder"]) 23538
#34 (depressi* or depressed or antidepress* or anti NEXT depress* or MDD or affective disorder* or affective NEXT symptom* or mood NEXT disorder?):ti,ab,kw 100975
#35 ([mh ^anxiety] or [mh ^"anxiety disorders"] or [mh ^agoraphobia] or [mh ^"anxiety, separation"] or [mh ^"neurocirculatory asthenia"] or [mh ^"neurotic disorders"] or [mh ^"obsessive-compulsive disorder"] or [mh ^"hoarding disorder"] or [mh ^"panic disorder"] or [mh ^"phobic disorders"] or [mh ^"phobia, social"]) 15332
#36 (anxiety or GAD or agoraphobi* or claustrophobi* or neurocirculatory asthenia or neurotic or neuros* or obsess* or compulsi* or OCD or hoarding or panic or fear or worry or worries or phobi*):ti,ab,kw 78667
#37 ([mh ^"adjustment disorders"] or [mh ^"psychological trauma"] or [mh ^"trauma and stressor related disorders"] or [mh ^"stress disorders, traumatic"] or [mh ^"stress disorders, post-traumatic"] or [mh ^"stress disorders, traumatic, acute"]) 3414
#38 (adjustment NEXT disorder? or (stress NEAR/2 disorder?)):ti,ab,kw 7287
#39 (PTSD or posttraumatic NEXT stress or post NEXT traumatic or "impact of event?"):ti,ab,kw 8391
#40 ([mh ^"sleep wake disorders"] or [mh ^"sleep initiation and maintenance disorders"]) 4503
#41 (insomni* or sleep* or wake*):ti,ab,kw 52544
#42 ([mh ^"self-injurious behavior"] or [mh ^"self mutilation"] or [mh ^suicide] or [mh ^"suicidal ideation"] or [mh ^"suicide, assisted"] or [mh ^"suicide, attempted"] or [mh ^"suicide, completed"]) 1674
#43 (NSSI* or ((nonsuicid* or non NEXT suicid*) adj2 (self* or injur*))):ti,ab,kw 76
#44 (suicid* or parasuicid* or auto NEXT mutilat* or automutilat* or self NEXT destruct* or selfdestruct* or self NEXT harm* or selfharm* or self NEXT immolat* or selfimmolat* or self NEXT inflict* or selfinflict* or self NEXT injur* or selfinjur* or selfmutilat* or self NEXT mutilat* or self NEXT poison* or selfpoison* or (self NEAR/2 (cut or cuts or cutting or cutter* or burn or burns or burning or bite or bites or biting or hit or hits or hitting)) or head NEXT bang* or headbang*):ti,ab,kw 7772
#45 ([mh ^"substance-related disorders"] or [mh "alcohol-related disorders"] or [mh ^"marijuana abuse"] or [mh ^"tobacco use disorder"]) 11479
#46 ([mh Smoking] or [mh ^Tobacco] or [mh ^"Tobacco Products"] or [mh ^Nicotine]) 8405
#47 (tobacco* or cigar* or cigarette* or nicotine or smoking or smoker?):ti,ab,kw 42563
#48 ([mh ^Vaping] or [mh ^"Electronic Nicotine Delivery Systems"]) 214
#49 (vape or vaper or vapers or vaping):ti,ab,kw 266
#50 (ecig* or e NEXT cig* or (electr* NEAR/2 (cig* or nicotine or device*))):ti,ab,kw 2885
#51 (nicotine NEAR/4 (electr* or ENDS or aerosol*)):ti,ab,kw 387
#52 ([mh ^"alcohol related disorders"] or [mh ^alcoholism] or [mh ^"alcohol abstinence"] or [mh ^"alcohol intoxication"]) 4278
#53 (alcohol or liquor):ti or alcoholi*:ti,ab,kw or (alcohol* NEAR/3 (abuse* or addict* or dependen* or disorder* or abstinen*)):ti,ab,kw 21739
#54 ((problem* or underage? or under NEXT age?) NEAR/2 (drink* or alcohol* NEXT use*)):ti,ab,kw 1164
#55 ([mh Cannabinoids] or [mh ^Cannabis] or [mh ^"Marijuana Smoking"] or [mh ^"Marijuana Abuse"]) 1790
#56 (cannabi* or marijuana or marihuana or hash or hashish or skunk or ganja or sinsemilia):ti,ab,kw 4820
#57 {OR #29-#56} 267743
#58 #13 and #28 and #57 289
#59 [mh ^quarantine/px] 3
#60 #58 or #59 in Trials 285
ClinicalTrials.gov
100 studies found for:
Condition OR disease: (depression OR anxiety OR phobia OR PTSD OR panic OR OCD OR stress OR neurosis)
Other terms: ((movement control order OR house bound OR stay at home order OR isolation) AND (pandemic OR epidemic OR covid OR contagion))
41 studies found for:
Condition OR disease: ((mental OR psychological) AND (health or well being or wellbeing))
Other terms: ((movement control order OR house bound OR stay at home order OR isolation) AND (pandemic OR epidemic OR covid OR contagion))
61 studies found for:
Condition OR disease: (depression OR anxiety OR phobia OR PTSD OR panic OR OCD OR stress OR neurosis)
Other terms: (lockdown OR lock down OR quarantine OR self-isolation OR curfew)
31 studies found for:
Condition OR disease: ((mental OR psychological) AND (health or well being or wellbeing))
Other terms: (lockdown OR lock down OR quarantine OR self-isolation OR curfew)
WHO ICTRP
Title: ((movement control order OR house bound OR stay at home order OR isolation) AND (pandemic OR epidemic OR covid OR contagion))
Condition: (depression OR anxiety OR phobia OR PTSD OR panic OR OCD OR stress OR neurosis)
Recruitment Status: ALL (11 records for 11 trials found)
Title: ((movement control order OR house bound OR stay at home order OR isolation) AND (pandemic OR epidemic OR covid OR contagion))
Condition: ((mental OR psychological) AND (health or well being or wellbeing))
Recruitment Status: ALL (7 records for 7 trials found)
Title: (lockdown OR lock down OR quarantine OR self-isolation OR curfew)
Condition: (depression OR anxiety OR phobia OR PTSD OR panic OR OCD OR stress OR neurosis)
Recruitment Status: ALL (12 records for 12 trials found)
Title: (lockdown OR lock down OR quarantine OR self-isolation OR curfew)
Condition: ((mental OR psychological) AND (health or well being or wellbeing))
Recruitment Status: ALL (9 records for 9 trials found)

Supplementary material 3 

**Table 6:** Characteristics of excluded studies

Title	Author	Year	Reason of exclusion	Digital object identifier
COVID-19 related anxiety in children and adolescents with severe obesity: a mixed-methods study	Abawi et al.	2020	Ineligible outcomes	10.1111/cob.12412
The early impact of the COVID-19 pandemic on acute care mental health services	Abbas et al.	2021	Ineligible outcomes	10.1176/APPI.PS.202000467
Impact of social distancing on the mental health of parents and children in Qatar	Abdelrahman et al.	2022	Ineligible study design	10.1007/s11469-021-00555-6
Assessment of anxiety and depression, and coping mechanisms during COVID-19 lockdown among pregnant women	Abdus-Salam et al.	2022	Ineligible study design	10.1016/J.HELIYON.2022.E10902
Anxiety and gastrointestinal symptoms related to COVID-19 during Italian lockdown	Abenavoli et al.	2021	Ineligible study design	10.3390/jcm10061221
Prevalence and factors associated with mental health impact of COVID-19 pandemic in Bangladesh: a survey-based cross-sectional study	Abir et al.	2021	Ineligible study design	10.5334/AOGH.3269
Psychological Impact of COVID-19 pandemic in Bangladesh: analysis of a cross-sectional survey	Abir et al.	2021	Ineligible study design	10.1089/hs.2020.0205
The relationship between common mental disorders (CMDs), food insecurity and domestic violence in pregnant women during the COVID-19 lockdown in Cape Town, South Africa	Abrahams et al.	2022	Ineligible outcomes	10.1007/s00127-021-02140-7
Life with corona: increased gender differences in aggression and depression symptoms due to the COVID-19 pandemic burden in Germany	Abreu et al.	2021	Ineligible study design	10.3389/fpsyg.2021.689396
Dental students' discomfort and anxiety during the first and the second lockdown due to COVID-19 pandemic at the School of Dental Medicine, University of Zagreb	Adam et al.	2021	Ineligible control	10.15644/ASC55/2/8
Psychological effects of the COVID-19 imposed lockdown on adults with attention deficit/hyperactivity disorder: cross-sectional survey study	Adamou et al.	2020	Ineligible study design	10.2196/24430
Impact of COVID-19 "stay home, stay healthy" orders on function among older adults participating in a community-based, behavioral intervention study	Adams et al.	2021	Ineligible study design	10.1177/0898264321991314
The mental health of Australian medical practitioners during COVID-19	Adams et al.	2021	Ineligible study design	10.1177/10398562211010807
Evaluation of anxiety, depression and sleep quality in full-time teleworkers	Afonso et al.	2020	Ineligible study design	10.1093/pubmed/fdab164
Mental well-being and association of the four factors coping structure model: a perspective of people living in lockdown during COVID-19	Agha et al.	2021	Ineligible study design	10.1016/j.jemep.2020.100605
The coronavirus stress: a reality check of India's mental health social agenda	Agoramoorthy et al.	2020	Others - letter to the editor	10.1177/0020764020925498
Psychological impact of the COVID-19 pandemic and social determinants on the Portuguese population: protocol for a web-based cross-sectional study	Aguiar et al.	2021	Others - study protocol	10.2196/28071
Impact of the societal response to COVID-19 on access to healthcare for non-COVID-19 health issues in slum communities of Bangladesh, Kenya, Nigeria and Pakistan: results of pre-COVID-19 and COVID-19 lockdown stakeholder engagements	Ahmed et al.	2020	Ineligible outcomes	10.1136/bmjgh-2020-003042
Impact of COVID-19 lockdown on mental health in Germany: longitudinal observation of different mental health trajectories and protective factors	Ahrens et al.	2021	Ineligible study design	10.1038/s41398-021-01508-2
Differential impact of COVID-related lockdown on mental health in Germany	Ahrens et al.	2021	Ineligible outcomes	10.1002/WPS.20830
Who is really at risk? The contribution of death anxiety in suicide risk and loneliness among older adults during the COVID-19 pandemic	Aisenberg-Shafran et al.	2022	Ineligible study design	10.1080/07481187.2021.1947416
Effect of COVID 19 lockdown on the lifestyle and dietary diversity of women handloom workers	Aiswarya et al.	2021	Ineligible outcomes	10.1016/J.CEGH.2021.100856
COVID-19-related anxiety in phenylketonuria patients	Akar et al.	2021	Ineligible study design	10.24953/TURKJPED.2021.05.007
COVID-19 and mental health/substance use disorders on Reddit: a longitudinal study	Alambo et al.	2021	Ineligible outcomes	10.1007/978-3-030-68790-8_2
Effect of SARS-CoV-2 (COVID-19) pandemic and lockdown on body weight, maladaptive eating habits, anxiety, and depression in a bariatric surgery waiting list cohort	Albert et al.	2021	Ineligible study design	10.1007/s11695-021-05257-5
Association between homeschooling and adolescent sleep duration and health during COVID-19 pandemic high school closures	Albrecht et al.	2022	No appropriate data	10.1001/jamanetworkopen.2021.42100
Craving variations in patients with substance use disorder and gambling during COVID-19 lockdown: the Italian experience	Alessi et al.	2022	Ineligible study design	10.12998/wjcc.v10.i3.882
Dietary intake and mental health among Saudi Adults during COVID-19 lockdown	Alfawaz et al.	2021	Ineligible study design	10.3390/IJERPH18041653
Psychological well-being during COVID-19 lockdown: insights from a Saudi State University's Academic Community	Alfawaz et al.	2021	Ineligible study design	10.1016/J.JKSUS.2020.101262
COVID-19 lockdown and poor sleep quality: not the whole story	Alfonsi et al.	2021	Ineligible study design	10.1111/jsr.13368
Mental health and its association with coping strategies and intolerance of uncertainty during the COVID-19 pandemic among the general population in Saudi Arabia: cross-sectional study	Alhadi et al.	2021	Ineligible study design	10.1186/s12888-021-03370-4
The prevalence of depression and related factors during the COVID-19 pandemic among the general population of the Jazan Region of Saudi Arabia	Alharbi et al.	2022	Ineligible study design	10.7759/cureus.21965
Depression, suicidal thoughts, and burnout among physicians during the COVID-19 pandemic: a survey-based cross-sectional study	Al-Humadi et al.	2021	Ineligible study design	10.1007/s40596-021-01490-3
Depression and obsessive-compulsive disorders amid the COVID-19 pandemic in Saudi Arabia	Alhusseini et al.	2021	Ineligible study design	10.7759/cureus.12978
Evaluation of COVID-19 disease awareness and its relation to mental health, dietary habits, and physical activity: a cross-sectional study from Pakistan	Ali et al.	2021	Ineligible study design	10.4269/ajtmh.20-145
Effects of COVID-19 pandemic and lockdown on lifestyle and mental health of students: a retrospective study from Karachi, Pakistan	Ali et al.	2022	Ineligible study design	10.1016/J.AMP.2021.02.004
Physical distancing behavior: the role of emotions, personality, motivations, and moral decision-making	Alivernini et al.	2021	Ineligible outcomes	10.1093/jpepsy/jsaa122
Perceptions towards COVID-19 and adoption of preventive measures among the public in Saudi Arabia: a cross sectional study	Alkhaldi et al.	2021	Ineligible control	10.1186/s12889-021-11223-8
The psychological impact of COVID-19 pandemic on the general population of Saudi Arabia	Alkhamees et al.	2020	Ineligible study design	10.1016/J.COMPPSYCH.2020.152192
The impact of COVID-19 pandemic and lockdown on alcohol consumption: a perspective from hair analysis	Alladio et al.	2021	Ineligible outcomes	10.3389/fpsyt.2021.632519
Self-isolation, psychotic symptoms and cognitive problems during the COVID-19 worldwide outbreak	Allé et al.	2021	Ineligible study design	10.1016/J.PSYCHRES.2021.114015
The role of the COVID-19 pandemic in altered psychological well-being , mental health and sleep: an online cross-sectional study	Allen et al.	2022	Ineligible study design	10.1080/13548506.2021.1916963
Impact of COVID-19 stay-at-home orders on weight-related behaviours among patients with obesity	Almandoz et al.	2020	Ineligible study design	10.1111/COB.12386
Substance use mental health and weight-related behaviours during the COVID-19 pandemic in people with obesity	Almandoz et al.	2021	Ineligible study design	10.1111/cob.12440
The medical student response to the mental health consequences of COVID-19	Almazan et al.	2020	Others - letter to the editor	10.1007/s40596-020-01313-x
Short-term impact of social distancing measures during the COVID-19 pandemic on cognitive function and health perception of Brazilian older adults: a pre-post study	Almeida et al.	2021	Ineligible outcomes	10.1177/07334648211015458
Impact of COVID-19 on children's and adolescent's mental health in Saudi Arabia	Almhizai et al.	2021	Ineligible study design	10.7759/cureus.19786
Lifestyle changes associated with COVID-19 quarantine among young Saudi women: a prospective study	Al-Musharaf et al.	2021	Ineligible study design	10.1371/journal.pone.0250625
Efectos psicológicos de la pandemia COVID-19 en la población general de Argentina	Alomo et al.	2020	Ineligible study design	10.31053/1853.0605.v77.n3.28561
Mental impact of COVID-19 among Spanish healthcare workers. A large longitudinal survey	Alonso et al.	2022	Ineligible intervention	10.1017/S2045796022000130
A qualitative examination of the mental health impact of COVID-19 in marginalized communities in Guatemala: the COVID care calls survey	Alonzo et al.	2022	Ineligible study design	10.1177/00207640211028612
The current pandemic a complex emergency? Mental health impact of the COVID-19 pandemic on highly vulnerable communities in Guatemala	Alonzo et al.	2022	Ineligible study design	10.1177/00207640211027212
The effect of pre-quarantine physical activity on anxiety and depressive symptoms during the COVID-19 lockdown in the Kingdom of Saudi Arabia	Alotaibi et al.	2021	Ineligible study design	10.3390/ijerph18157771
The stress, sleep, physical activity, and pain level during the COVID outbreak	Alpozgen et al.	2022	Ineligible study design	Not applicable
COVID-19 pandemic: psycho-social consequences during the social distancing period among Najran City population	Al-Qahtani et al.	2020	Ineligible control	10.24869/PSYD.2020.280
Public response anxiety and behaviour during the first wave of COVID-19 pandemic in Saudi Arabia	Alqahtani et al.	2021	Ineligible study design	10.3390/ijerph18094628
Fall from grace: increased loneliness and depressiveness among extraverted youth during the German COVID-19 lockdown	Alt et al.	2021	Ineligible study design	10.1111/jora.12648
The psychological impact of COVID-19 pandemic and lockdown on caregivers of people with dementia	Altieri et al.	2021	Ineligible study design	10.1016/j.jagp.2020.10.009
Assessment of depression severity during coronavirus disease 2019 pandemic among the Palestinian population: a growing concern and an immediate consideration	Al et al.	2020	Ineligible study design	10.3389/fpsyt.2020.570065
Lockdown effects on healthy cognitive aging during the COVID-19 pandemic: a longitudinal study	Amanzio et al.	2021	Ineligible study design	10.3389/fpsyg.2021.685180
COVID-19 lockdown impact on mental health in a large representative sample of Italian adults	Amerio et al.	2021	Ineligible study design	10.1016/J.JAD.2021.05.117
Hope during COVID-19 lockdown	Amirav et al.	2021	Ineligible study design	10.7759/cureus.15097
Symptoms of anxiety/depression during the COVID-19 pandemic and associated lockdown in the community: longitudinal data from the TEMPO cohort in France	Andersen et al.	2021	Ineligible intervention	10.1186/s12888-021-03383-z
Risk of stress/depression and functional impairment in Denmark immediately following a COVID-19 shutdown	Andersen et al.	2021	Ineligible study design	10.1186/s12889-021-11020-3
Suicide in England in the COVID-19 pandemic: early observational data from real time surveillance	Appleby et al.	2021	Ineligible study design	10.1016/j.lanepe.2021.100110
Effect of the COVID-19 pandemic on anxiety in patients with masticatory muscle pain	Arifagaoglu et al.	2023	Ineligible study design	10.1016/J.PROSDENT.2021.09.002
Suicide in India during the first year of the COVID-19 pandemic	Arya et al.	2022	Ineligible intervention	10.1016/j.jad.2022.03.066
Burnout among adolescent population during COVID-19 lockdown in Sialkot Pakistan	Asif et al.	2021	Ineligible study design	10.32413/pjph.v11i2.750
Anxiety and depression symptoms in the same pregnant women before and during the COVID-19 pandemic	Ayaz et al.	2020	Ineligible study design	10.1515/jpm-2020-0380
Association of dyspnea and physical activity level in adult patients with CF during COVID-19	Aydan et al.	2023	Ineligible study design	10.1183/13993003.congress-2023.PA4525
The effect of COVID-19 pandemic on the sleep quality of patients who have the diagnosis of bipolar disorder	Aydınoğlu et al.	2021	Ineligible study design	10.5505/kpd.2020.26576
Changes on depression and suicidal ideation under severe lockdown restrictions during the first wave of the COVID-19 pandemic in Spain: a longitudinal study in the general population	Ayuso-Mateos et al.	2023	Ineligible outcomes	10.1017/S2045796023000677
The occurrence of anxiety disorders among Poles during the COVID-19 pandemic	Babicki et al.	2021	Ineligible study design	10.12740/PP/ONLINEFIRST/126230
Tendency to worry and fear of mental health during Italy's COVID-19 lockdown	Baiano et al.	2020	Ineligible study design	10.3390/ijerph17165928
COVID-19 impact on psychological outcomes of parents siblings and children with intellectual disability: longitudinal before and during lockdown design	Bailey et al.	2021	Ineligible outcomes	10.1111/jir.12818
Rates of self-reported postpartum depressive symptoms in the United States before and after the start of the COVID-19 pandemic	Bajaj et al.	2022	Ineligible study design	10.1016/j.jpsychires.2022.04.011
Effect of lockdown following COVID-19 pandemic on alcohol use and help-seeking behavior: observations and insights from a sample of alcohol use disorder patients under treatment from a tertiary care center	Balhara et al.	2020	Ineligible study design	10.1111/pcn.13075
Suicide epidemic in Malawi: what can we do?	Banda et al.	2021	Others - commentary	10.11604/pamj.2021.38.69.27843
Effects of COVID-19 pandemic confinement in patients with cognitive impairment	Barguilla et al.	2020	Ineligible study design	10.3389/fneur.2020.589901
Abstinence among alcohol use disorder patients during the COVID-19 pandemic: insights from Spain	Barrio et al.	2021	Ineligible outcomes	10.1111/acer.14555
Self-isolation: a significant contributor to cannabis use during the COVID-19 pandemic	Bartel et al.	2020	Ineligible study design	10.1080/08897077.2020.1823550
Impact of the COVID‑19 pandemic on quality of life and emotional wellbeing in patients with bone metastases treated with radiotherapy: a prospective cohort study	Bartels et al.	2021	Ineligible outcomes	10.1007/s10585-021-10079-x
Impact of the novel coronavirus disease on treatment adherence and sleep duration in patients with obstructive sleep apnea treated with positive airway pressure	Batool-Anwar et al.	2020	Ineligible outcomes	10.5664/jcsm.8746
Effects of the COVID-19 pandemic on suicidal ideation in a representative Australian population sample–Longitudinal cohort study	Batterham et al.	2022	Ineligible control	10.1016/j.jad.2022.01.022
Coronavirus lockdown: excessive alcohol consumption and illicit substance use in DUI subjects	Beccegato et al.	2021	Ineligible study design	10.1080/15389588.2021.1923701
COVID-19 health crisis and lockdown associated with high level of sleep complaints and hypnotic uptake at the population level	Beck et al.	2021	Ineligible study design	10.1111/jsr.13119
Alcohol consumption and COVID-19–related stress among health care workers: the need for continued stress-management interventions	Beiter et al.	2022	Ineligible intervention	10.1177/00333549211058176
An impact analysis of the early months of the COVID-19 pandemic on mental health in a prospective cohort of Canadian adolescents	Bélanger et al.	2021	Ineligible study design	10.1016/J.JADOHEALTH.2021.07.039
Evolution of psychosocial burden and psychiatric symptoms in patients with psychiatric disorders during the COVID‑19 pandemic	Belz et al.	2022	Ineligible outcomes	10.1007/s00406-021-01268-6
No party no drugs? Use of stimulants dissociative drugs and GHB/GBL during the early COVID-19 pandemic	Bendau et al.	2022	Ineligible intervention	10.1016/J.DRUGPO.2022.103582
Association between mental health trajectories and somatic symptoms following a second lockdown in Israel: a longitudinal study	Ben-Ezra et al.	2021	Ineligible control	10.1136/bmjopen-2021-050480
Stay-at-home orders due to the COVID-19 pandemic are associated with elevated depression and anxiety in younger but not older adults: results from a nationwide community sample of adults from Germany	Benke et al.	2022	No appropriate data	10.1017/S0033291720003438
Lockdown-related factors associated with the worsening of cardiovascular risk and anxiety or depression during the COVID-19 pandemic	Bérard et al.	2020	Ineligible study design	10.1016/J.PMEDR.2020.101300
One-year impact of COVID-19 lockdown-related factors on cardiovascular risk and mental health: a population-based cohort study	Bérard et al.	2022	Ineligible study design	10.3390/ijerph19031684
The impact of the COVID-19 pandemic on suicide ideation and suicide attempts in a sample of psychiatric inpatients	Berardelli et al.	2021	Ineligible outcomes	10.1016/J.PSYCHRES.2021.114072
Psychological burden of COVID-19 on mild and moderate chronic spontaneous urticarial	Beyaz et al.	2021	Ineligible control	10.2500/aap.2021.42.210026
Altered sleep duration and poor quality of sleep among pharmacy students amidst COVID‑19 lockdown: a South‑Indian study	Bhat et al.	2022	Ineligible outcomes	10.1007/s41782-021-00178-w
Impact of COVID-19 lockdown on self-harm and violence among patients presenting to the emergency department	Bhattaram et al.	2022	Ineligible outcomes	10.1016/J.AJEM.2021.11.008
Are the kids really alright? Impact of COVID-19 on mental health in a majority Black American sample of schoolchildren	Bhogal et al.	2021	Ineligible population	10.1016/j.psychres.2021.114146
Longitudinal increases in childhood depression symptoms during the COVID-19 lockdown	Bignardi et al.	2021	Ineligible population	10.1136/archdischild-2020-320372
Relationships among behavioural regulations physical activity and mental health pre- and during COVID–19 UK lockdown	Bird et al.	2021	Ineligible outcomes	10.1016/j.psychsport.2021.101945
The impact of the COVID-19 pandemic on the lifestyles and levels of anxiety and depression of patients with schizophrenia: a retrospective observational study	Biviá-Roig et al.	2022	Ineligible study design	10.3390/healthcare10010128
Longitudinal assessment of alcohol consumption throughout the first COVID-19 lockdown: contribution of age and pre-pandemic drinking patterns	Bollen et al.	2022	Ineligible study design	10.1159/000518218
The impact of COVID‑19 on the mental health of dialysis patients	Bonenkamp et al.	2021	Ineligible outcomes	10.1007/s40620-021-01005-1
Changes in alcohol use patterns in the united states during COVID-19 pandemic	Boschuetz et al.	2020	Ineligible study design	Not applicable
Psychological distress in the context of the COVID-19 pandemic: the joint contribution of intolerance of uncertainty and cyberchondria	Bottesi et al.	2022	Ineligible study design	10.1080/08870446.2021.1952584
Loneliness depression and anxiety experienced by the Israeli population during the first COVID-19 lockdown: a cross-sectional survey	Brafman et al.	2021	Ineligible study design	10.5041/rmmj.10449
Coronavirus (COVID-19) outbreak: addictive social media use depression anxiety and stress in quarantine - an exploratory study in Germany and Lithuania	Brailovskaia et al.	2021	Ineligible intervention	10.1016/J.JADR.2021.100182
Prospective impact of COVID-19 on mental health functioning in adolescents with and without ADHD: protective role of emotion regulation abilities	Breaux et al.	2021	Ineligible study design	10.1111/jcpp.13382
Prospective impact of COVID-19 on mental health functioning in adolescents with and without ADHD: protective role of emotion regulation abilities	Breaux et al.	2021	Ineligible study design	10.1111/JCPP.13382
Changes in sleep patterns and disorders in children and adolescents with attention deficit hyperactivity disorders and autism spectrum disorders during the COVID-19 lockdown	Bruni et al.	2021	Ineligible outcomes	10.3390/brainsci11091139
The impact of lockdown on sleep patterns of children and adolescents with ADHD	Bruni et al.	2021	Ineligible outcomes	10.5664/JCSM.9296
Impact of COVID-19 lockdown on sleep in children with autism spectrum disorders	Bruni et al.	2022	Ineligible outcomes	10.5664/JCSM.9518
Changes in sleep patterns and disturbances in children and adolescents in Italy during the COVID-19 outbreak	Bruni et al.	2022	Ineligible study design	10.1016/j.sleep.2021.02.003
Poor sleep quality and unhealthy lifestyle during the lockdown: an Italian study	Bruno et al.	2022	Ineligible study design	10.1016/J.SLEEP.2022.01.002
The impact of early stages of COVID-19 on the mental health of autistic adults in the United Kingdom: a longitudinal mixed-methods study	Bundy et al.	2022	Ineligible study design	10.1177/13623613211065543
Economic expectations and anxiety during the COVID-19 pandemic: a one-year longitudinal evaluation on Italian university students	Busetta et al.	2023	Ineligible control	10.1007/s11135-022-01330-y
Psychological health issues subsequent to SARS-Cov-2 restrictive measures: the role of parental bonding and attachment style	Bussone et al.	2020	Ineligible study design	10.3389/fpsyt.2020.589444
Psychological impact of COVID-19 on ICU caregivers	Caillet et al.	2020	Ineligible study design	10.1016/J.ACCPM.2020.08.006
Shifts in alcohol consumption during the COVID-19 pandemic: early indications from Australia	Callinan et al.	2021	Ineligible study design	10.1111/add.15275
Impact of the COVID-19 pandemic upon patients with burning mouth syndrome	Candela et al.	2022	Ineligible study design	10.1016/J.JORMAS.2021.07.001
Psychological consequences of COVID-19 pandemic in Italian MS patients: signs of resilience ?	Capuano et al.	2021	Ineligible study design	10.1007/s00415-020-10099-9
Associations between mental health, alcohol consumption and drinking motives during COVID-19 second lockdown in Ireland	Carbia et al.	2022	Ineligible intervention	10.1093/alcalc/agab067
Stress/depression across the COVID-19 pandemic in Denmark	Cardona et al.	2023	Ineligible outcomes	10.1186/s12889-023-15129-5
Characterizing changes in screen time during the COVID-19 pandemic school closures in Canada and its perceived impact on children with autism spectrum disorder	Cardy et al.	2021	Ineligible outcomes	10.3389/fpsyt.2021.702774
Impact of COVID-19 lockdown on suicide attempts: a retrospective analysis of the springtime admissions to the trauma resuscitation room at the Medical University of Vienna from 2015–2020	Carlin et al.	2021	Ineligible outcomes	10.1007/s00508-021-01839-6
Changes in substance use among people seeking alcohol and other drug treatment during the COVID-19 pandemic: evaluating mental health outcomes and resilience	Carlyle et al.	2021	Ineligible study design	10.1177/11782218211061746
Impact of COVID-19 lockdown on smoking consumption in a large representative sample of Italian adults	Carreras et al.	2022	Ineligible study design	10.1136/tobaccocontrol-2020-056440
Lockdown impact on lifestyle and its association with oral parafunctional habits and bruxism in a Spanish adolescent population	Carrillo-Diaz et al.	2022	Ineligible study design	10.1111/ipd.12843
A longitudinal observation of general psychopathology before the COVID-19 outbreak and during lockdown in Italy	Castellini et al.	2021	Ineligible study design	10.1016/j.jpsychores.2020.110328
Has the COVID-19 pandemic changed the daily practices and psychological state of orthopaedic residents?	Castioni et al.	2021	Ineligible study design	10.1097/CORR.0000000000001728
Evaluation of quality of life and physical activity in patients with type 1 diabetes mellitus during the COVID-19 pandemic	Çelik et al.	2023	Ineligible intervention	10.20945/2359-3997000000531
Changes in sleep pattern, sense of time and digital media use during COVID-19 lockdown in Italy	Cellini et al.	2020	Ineligible study design	10.1111/jsr.13074
Prevalence of depression, anxiety, and stress among high school students during the COVID-19 pandemic: a survey study in Western Mexico	Cervantes-Cardona et al.	2022	Ineligible study design	10.3390/IJERPH192316154
Adolescents' substance use and physical activity before and during the COVID-19 pandemic	Chaffee et al.	2021	Ineligible outcomes	10.1001/jamapediatrics.2021.0541
Impact of lockdown on patients with congestive heart failure during the coronavirus disease 2019 pandemic	Chagué et al.	2020	Ineligible study design	10.1002/ehf2.13016
Elderly suicide and the 2003 SARS epidemic in Hong Kong	Chan et al.	2006	Ineligible intervention	10.1002/gps.1432
Breastfeeding practices and postpartum depression in Mexican women during the COVID-19 pandemic: a cross-sectional study	Chávez-Tostado et al.	2023	Ineligible study design	10.3390/medicina59071330
The medium-term impact of COVID-19 lockdown on referrals to secondary care mental health services: a controlled interrupted time series study	Chen et al.	2020	Ineligible outcomes	10.3389/fpsyt.2020.585915
Problematic internet-related behaviors mediate the associations between levels of internet engagement and distress among schoolchildren during COVID-19 lockdown: a longitudinal structural equation modeling study	Chen et al.	2021	Ineligible study design	10.1556/2006.2021.00006
The relationship between children's problematic Internet-related behaviors and psychological distress during the onset of the COVID-19 pandemic: a longitudinal study	Chen et al.	2022	Ineligible study design	10.1097/ADM.0000000000000845
Impact of COVID-19 on the health and psychosocial status of vulnerable older adults: study protocol for an observational study	Cheung et al.	2020	Others - study protocol	10.1186/s12889-020-09900-1
Impact of the first wave of COVID-19 on the health and psychosocial well-being of Māori, Pacific Peoples and New Zealand Europeans living in aged residential care	Cheung et al.	2022	Ineligible study design	10.1111/ajag.13025
Relationships between changes in self-reported physical activity, sedentary behaviour and health during the coronavirus (COVID-19) pandemic in France and Switzerland	Cheval et al.	2021	Ineligible study design	10.1080/02640414.2020.1841396
COVID-19 pandemic response behaviors: a Singapore experience of the "circuit breaker"	Chew et al.	2021	Ineligible outcomes	10.1093/tbm/ibaa135
The Impact of SARS-CoV-2 (COVID-19) and its lockdown measures on the mental and functional health of older	Chiara et al.	2021	Ineligible outcomes	10.1007/s11126-021-09943-6
The impact of COVID-19 on the safety, housing stability, and mental health of unstably housed domestic violence survivors	Chiaramonte et al.	2022	No appropriate data	10.1002/jcop.22765
Effects of the change in activity participation during the COVID-19 pandemic on children’s mental health	Chien et al.	2022	Ineligible outcomes	10.5014/ajot.2022.047118
Alcohol consumption reported during the COVID-19 pandemic: the initial stage	Chodkiewicz et al.	2020	Ineligible control	10.3390/ijerph17134677
The importance of sleep and physical activity on well-being during COVID-19 lockdown: reunion island as a case study	Chouchou et al.	2021	Ineligible study design	10.1016/j.sleep.2020.09.014
Impact of the first COVID-19 outbreak on mental health service utilisation at a Dutch mental health centre: retrospective observational study	Chow et al.	2021	Ineligible outcomes	10.1192/bjo.2021.1049
An Internet-based study on the impact of COVID-19 pandemic-related lockdown on migraine in India	Chowdhury et al.	2021	Ineligible outcomes	10.1111/ane.13525
The need for additional mental health support for women in the postpartum period in the times of epidemic crisis	Chrzan-Dętkoś et al.	2021	Ineligible intervention	10.1186/s12884-021-03544-8
Person-centered patterns of substance use during the COVID-19 pandemic and their associations with COVID-related impacts on health and personal finances in young Black and White women	Chung et al.	2022	Ineligible study design	10.1016/j.drugalcdep.2022.109620
The effects of COVID-19 lockdown on lifestyle and emotional state in women undergoing assisted reproductive technology: results of an Italian survey	Cirillo et al.	2021	Ineligible outcomes	10.1016/j.jogoh.2021.102079
Impact of COVID-19 on 'living well' with mild-to-moderate dementia in the community: findings from the IDEAL cohort	Clare et al.	2022	Ineligible outcomes	10.3233/JAD-215095
Violence in intimate partnerships and mental problems in children and adolescents: online survey during the COVID-19 pandemic	Clemens et al.	2021	Ineligible outcomes	10.1007/s00278-021-00501-w
Pain experience and mood disorders during the lockdown of the COVID-19 pandemic in the United States: an opportunistic study	Colloca et al.	2021	Ineligible study design	10.1097/PR9.0000000000000958
COVID-19 lockdowns' effects on the quality of life, perceived health and well-being of healthy elderly individuals: a longitudinal comparison of pre-lockdown and lockdown states of well-being	Colucci et al.	2022	Ineligible outcomes	10.1016/J.ARCHGER.2021.104606
The association between changes in the university educational setting and peer relationships: effects in students' depressive symptoms during the COVID-19 pandemic	Conceição et al.	2021	Ineligible study design	10.3389/fpsyt.2021.783776
Dissociated profiles of sleep timing and sleep quality changes across the first and second wave of the COVID-19 pandemic	Conte et al.	2021	No appropriate data	10.1016/j.jpsychires.2021.09.025
Influence of the COVID-19 outbreak on disease activity and quality of life in inflammatory bowel disease patients	Conti et al.	2021	Ineligible study design	10.3389/fpsyt.2021.664088
Psychological health status of psychiatric patients living in treatment communities before and during the covid-19 lockdown: a brief report	Cordellieri et al.	2021	Ineligible outcomes	10.3390/ijerph18073567
Maintaining social support while social distancing: the longitudinal benefit of basic psychological needs for symptoms of anxiety during the COVID-19 outbreak	Costa et al.	2022	Ineligible control	10.1111/jasp.12870
Loneliness, physical activity, and mental health during COVID-19: a longitudinal analysis of depression and anxiety in adults over the age of 50 between 2015 and 2020	Creese et al.	2021	Ineligible study design	10.1017/S1041610220004135
GROUPS 4 HEALTH protects against unanticipated threats to mental health: evaluating two interventions during COVID-19 lockdown among young people with a history of depression and loneliness	Cruwys et al.	2021	Ineligible study design	10.1016/j.jad.2021.08.029
COVID-19 and mental health: impact on symptom burden in older people living with mental illness in residential aged care	Curran et al.	2022	Ineligible study design	10.1111/ajag.13042
Neuropsychiatric symptoms in patients with Alzheimer’s disease during SARS-COV-2 pandemic in Peru	Custodio et al.	2021	Ineligible study design	10.1177/15333175211039089
Mental health, substance use, and suicidal ideation during a prolonged COVID-19-related lockdown in a region with low SARS-CoV-2 prevalence	Czeisler et al.	2021	Ineligible control	10.1016/j.jpsychires.2021.05.080
Health behaviours of young adults during the outbreak of the COVID-19 pandemic – a longitudinal study	Czenczek-Lewandowska et al.	2021	Ineligible study design	10.1186/s12889-021-11140-w
The effect of quarantine due to COVID-19 pandemic on seizure frequency in 102 adult people with epilepsy from Apulia and Basilicata regions, Southern Italy	d'Orsi et al.	2021	Ineligible outcomes	10.1016/j.clineuro.2021.106592
Mental health and wellbeing of 9–12-year-old children in Northern Canada before the COVID-19 pandemic and after the first lockdown	Dabravolskaj et al.	2021	Ineligible outcomes	10.3389/ijph.2021.1604219
Depression and anxiety before and during the COVID-19 lockdown: a longitudinal cohort study with university students	da et al.	2021	Ineligible study design	2021.02.23.21252284-2021.02.23.21252284
Association between psychological stress and neck pain among college students during the coronavirus disease of 2019 pandemic: a questionnaire-based cross-sectional study	Daher et al.	2021	Ineligible study design	10.3390/healthcare9111526
Mental health during the covid-19 lockdown over the christmas period in austria and the effects of sociodemographic and lifestyle factors	Dale et al.	2021	Ineligible control	10.3390/ijerph18073679
High-risk drinking in midlife before versus during the COVID-19 crisis: longitudinal evidence from the United Kingdom	Daly et al.	2021	Ineligible study design	10.1016/J.AMEPRE.2020.09.004
Depression reported by US adults in 2017–2018 and March and April 2020	Daly et al.	2021	No appropriate data	10.1016/j.jad.2020.09.065
Anxiety reported by US adults in 2019 and during the 2020 COVID-19 pandemic: population-based evidence from two nationally representative samples	Daly et al.	2022	Ineligible intervention	10.1016/j.jad.2021.02.054
Moms are not OK: COVID-19 and maternal mental health	Davenport et al.	2020	Ineligible study design	10.3389/fgwh.2020.00001
The COVID-19 lockdown and changes in routine-oriented lifestyle behaviors and symptoms of depression, anxiety, and insomnia in South Africa	Davy et al.	2021	No appropriate data	10.1123/jpah.2020-0863
Changes in Brazilians’ socioeconomic and health conditions during the COVID-19 pandemic	de Almeida et al.	2020	Ineligible study design	10.1590/1980-549720200105
Asthma patients experience increased symptoms of anxiety, depression and fear during the COVID-19 pandemic	de Boar et al.	2021	Ineligible study design	10.1177/14799731211029658
Psychological impact of the SARS-CoV-2 pandemic in children with neurodevelopmental disorders and their families: evaluation before and during COVID-19 outbreak among an Italian sample	De Giacomo et al.	2021	Ineligible outcomes	10.1708/3654.36348
When residents work less, they feel better: lessons learned from an unprecedent context of lockdown	Degraeve et al.	2020	Ineligible outcomes	10.1016/j.purol.2020.08.005
The short-term psychological impact of the COVID-19 pandemic in psychiatric patients: evidence for differential emotion and symptom trajectories in Belgium	Dejonckheere et al.	2021	Others - descriptive study	10.5334/PB.1028
Evaluation of copeptin and psychological stress among healthcare providers during COVID-19 pandemic	Demerdash et al.	2021	Ineligible intervention	10.1080/11101849.2021.1925442
Decreases in smoking and vaping during COVID-19 stay-at-home orders among a cohort of young adults in the United States	Denlinger-Apte et al.	2022	Ineligible study design	10.1016/j.ypmed.2022.106992
When will this end? Will it end?' the impact of the March-June 2020 UK COVID-19 lockdown response on mental health: a longitudinal survey of mothers in the Born in Bradford study	Dickerson et al.	2022	Ineligible study design	10.1136/bmjopen-2020-047748
The impact of COVID-19 pandemic on Italian university students’ mental health: changes across the waves	Di et al.	2021	Ineligible study design	10.3390/ijerph18189897
Short-term effects of COVID-19 lockdown in Italian children and adolescents with type 1 diabetes mellitus: the role of separation anxiety	Di et al.	2021	Ineligible outcomes	10.3390/ijerph18115549
Effect of confinement during COVID-19 outbreak on sleep quality in Galicia	Diz-Ferreira et al.	2021	Ineligible study design	e202101001-e202101001
The effects of COVID-19 lockdown on health and psychosocial functioning in older adults aged 70 and over	Docherty et al.	2021	Ineligible study design	10.1177/23337214211039974
Mental health of individuals infected with SARS-CoV-2 during mandated isolation and compliance with recommendations-a population-based cohort study	Domenghino et al.	2022	Ineligible intervention	10.1371/journal.pone.0264655
Changes over time in anxiety, depression, and stress symptoms among healthcare workers in French emergency departments during the first COVID-19 outbreak	Douplat et al.	2022	Ineligible study design	10.1016/J.JAD.2022.08.028
Swiss university students’ risk perception and general anxiety during the COVID-19 pandemic	Dratva et al.	2020	Ineligible control	10.3390/ijerph17207433
Time and COVID-19 stress in the lockdown situation: time free, "Dying" of boredom and sadness	Droit-Volet et al.	2020	Ineligible outcomes	10.1371/journal.pone.0236465
Psychological wellness of internal medicine hospitalists during the COVID-19 pandemic pandemic	Dugani et al.	2021	Ineligible intervention	10.1080/21548331.2020.1832792
What does adolescent substance use look like during the COVID-19 pandemic? Examining changes in frequency, social contexts, and pandemic-related predictors	Dumas et al.	2020	Ineligible study design	10.1016/J.JADOHEALTH.2020.06.018
Changes in suicide rates — United States, 2019 and 2020	Ehlman et al.	2022	Ineligible intervention	10.15585/mmwr.mm7108a5
Higher depression of patients with Alzheimer's disease during than before the lockdown	El et al.	2021	Ineligible study design	10.3233/JAD-210190
No impact of confinement during COVID-19 pandemic on anxiety and depression in Parkinsonian patients	El et al.	2021	Ineligible control	10.1016/J.NEUROL.2021.01.005
Students under lockdown: comparisons of students' social networks and mental health before and during the COVID-19 crisis in Switzerland	Elmer et al.	2020	Ineligible study design	10.1371/journal.pone.0236337
Analyses of posts written in online eating disorder and depression/anxiety moderated communities: emotional and informational communication before and during the COVID-19 outbreak	Elran-barak et al.	2021	Ineligible population	10.1016/j.invent.2021.100438
Gambling by young adults in the UK During COVID‑19 lockdown	Emond et al.	2022	Ineligible outcomes	10.1007/s10899-021-10029-y
Bullying, cyberbullying, anxiety, and depression in a sample of youth during the coronavirus pandemic	Englander et al.	2021	Ineligible study design	10.3390/PEDIATRIC13030064
Prevalence of depressive symptoms in patients with psoriatic arthritis: have numbers changed during the COVID-19 pandemic?	Englbrecht et al.	2021	Ineligible study design	10.3389/fmed.2021.748262
Pseudoscientific beliefs and psychopathological risks increase after COVID-19 social quarantine	Escolà-Gascón et al.	2020	Ineligible study design	10.1186/s12992-020-00603-1
Associations between anxiety and the willingness to be exposed to COVID-19 risk among French young adults during the first pandemic wave	Etilé et al.	2022	Ineligible study design	10.1371/journal.pone.0262368
Prevalence of depression symptoms in US adults before and during the COVID-19 pandemic	Ettman et al.	2020	Ineligible study design	10.1001/jamanetworkopen.2020.19686
Effects of the COVID-19 lockdown on mental health, wellbeing, sleep, and alcohol use in a UK student sample	Evans et al.	2022	Ineligible study design	10.1016/j.psychres.2021.113819
Psychedelic experiences during the early COVID-19 pandemic: findings from an international online survey	Evens et al.	2021	Ineligible intervention	10.3389/fpsyt.2021.732028
The relationship between acceptance and sleep–wake quality before, during, and after the first Italian COVID-19 lockdown	Fabbri et al.	2022	No appropriate data	10.3390/clockssleep4010016
Lock-down effect on the mental health status of healthcare workers during COVID-19 pandemic	Fageera et al.	2021	Ineligible control	10.3389/fpsyt.2021.683603
Prospective longitudinal study of 'sleepless in lockdown': unpacking differences in sleep loss during the coronavirus pandemic in the UK	Falkingham et al.	2022	Ineligible study design	10.1136/bmjopen-2021-053094
The impact of Covid-19-related distress on general health, oral behaviour, psychosocial features, disability and pain intensity in a cohort of Italian patients with temporomandibular disorders	Falla et al.	2021	Ineligible intervention	10.1371/journal.pone.0245999
Changes in tobacco use patterns among veterans in San Diego during the recent peak of the COVID-19 pandemic	Fatollahi et al.	2021	Ineligible outcomes	10.3390/ijerph182211923
The importance of physical activity to augment mood during COVID-19 lockdown	Fennell et al.	2022	Ineligible study design	10.3390/ijerph19031270
Impact of the COVID-19 pandemic in the Portuguese population: consumption of alcohol, stimulant drinks, illegal substances, and pharmaceuticals	Fernandes et al.	2021	Ineligible study design	10.1371/journal.pone.0260322
A longitudinal study on maternal depressive symptoms during the COVID-19 pandemic: the role of strict lockdown measures and social support	Fernandes et al.	2022	No appropriate data	10.3389/ijph.2022.1604608
Mental health and illness of medical students and newly graduated doctors during the pandemic of SARS-Cov-2/COVID-19	Ferreira et al.	2021	Ineligible intervention	10.1371/journal.pone.0251525
Quality of life under the COVID‑19 quarantine	Ferreira et al.	2021	Ineligible study design	10.1007/s11136-020-02724-x
COVID-19-related psychological and psychosocial distress among parents and youth with physical illness: a longitudinal study	Ferro et al.	2021	Ineligible study design	10.3389/fpsyt.2021.761968
Depression and anxiety symptoms remained elevated after 10 months of the COVID-19 pandemic in southern Brazil: findings from the PAMPA cohort	Feter et al.	2022	Ineligible study design	10.1016/J.PUHE.2021.12.019
Cognitive and mental health changes and their vulnerability factors related to COVID-19 lockdown in Italy	Fiorenzato et al.	2021	Ineligible study design	10.1371/journal.pone.0246204
Substance use, depression, and loneliness among American veterans during the COVID-19 pandemic	Fitzke et al.	2021	Ineligible study design	10.1111/ajad.13211
The impact of COVID-19 stay-at-home orders on health behaviors in adults	Flanagan et al.	2021	Ineligible study design	10.1002/oby.23066
The early impact of the COVID-19 lockdown on stress and addictive behaviors in an alcohol-consuming student population in France	Flaudias et al.	2021	Ineligible study design	10.3389/fpsyt.2021.628631
Predictors and patterns of gambling behaviour across the COVID-19 lockdown: findings from a UK cohort study	Fluharty et al.	2022	Ineligible outcomes	10.1016/j.jad.2021.10.117
Impact of the COVID-19 lockdown in Malaysia: an examination of the psychological well-being of parent-child dyads and child behavior in families with children on the autism spectrum	Fong et al.	2021	Ineligible study design	10.3389/fpsyt.2021.733905
Prevalence and risk factors of psychiatric symptoms among Swiss elite athletes during the first lockdown of the COVID-19 pandemic	Fröhlich et al.	2021	Ineligible study design	10.3390/ijerph182010780
The COVID-19 pandemic and mental health of first-year college students: examining the effect of COVID-19 stressors using longitudinal data	Fruehwirth et al.	2021	Ineligible study design	10.1371/journal.pone.0247999
The short-term effect of COVID-19 pandemic on disability, pain intensity, psychological status, and exercise habits in patients with chronic pain	Fujiwara et al.	2021	Ineligible study design	10.1007/s00540-021-02992-y
Problematic use of Internet-related activities and perceived weight stigma in schoolchildren: a longitudinal study across different epidemic periods of COVID-19 in China	Fung et al.	2021	Ineligible study design	10.3389/fpsyt.2021.675839
Social distancing and influenza mortality in 1918 did not increase suicide rates in the United States	Gaddy et al.	2021	Ineligible study design	10.1016/j.ssmph.2021.100944
Self-reported wellbeing and health-related quality of life of Aboriginal and Torres Strait Islander people pre and post the first wave of the COVID-19 2020 pandemic	Gall et al.	2022	Ineligible outcomes	10.1111/1753-6405.13199
Use of electronic cigarettes and heated tobacco products during the COVID-19 pandemic	Gallus et al.	2022	Ineligible study design	10.1038/s41598-021-04438-7
Changes in health behaviors, mental and physical health among older adults under severe lockdown restrictions during the COVID-19 pandemic in Spain	García-Esquinas et al.	2021	No appropriate data	10.3390/ijerph18137067
Lockdown strictness and mental health effects among older populations in Europe	García-Prado et al.	2022	Ineligible outcomes	10.1016/j.ehb.2022.101116
Factors associated with drinking behaviour during COVID-19 social distancing and lockdown among adults in the UK	Garnett et al.	2021	Ineligible study design	10.1016/j.drugalcdep.2020.108461
Changes in alcohol consumption and determinants of excessive drinking during the COVID-19 lockdown in the Slovak Republic	Gavurova et al.	2022	Ineligible study design	10.3389/fpubh.2021.791077
The significance of demographic variables on psychosocial health from the early stage and nine months after the covid-19 pandemic outbreak. A cross-national study	Geirdal et al.	2021	Ineligible study design	10.3390/ijerph18084345
Changes in tobacco use during the 2020 COVID-19 lockdown in New Zealand	Gendall et al.	2021	Ineligible study design	10.1093/ntr/ntaa257
Police-reported suicides during the first 16 months of the COVID-19 pandemic in Ecuador: a time-series analysis of trends and risk factors until June 2021	Gerstner et al.	2022	Ineligible intervention	10.1016/j.lana.2022.100324
Adding stress to the stressed: senior high school students' mental health amidst the COVID-19 nationwide lockdown in Greece	Giannopoulou et al.	2021	Ineligible study design	10.1016/J.PSYCHRES.2020.113560
Eating behaviour and symptom trajectories in patients with a history of binge eating disorder during COVID-19 pandemic	Giel et al.	2021	Ineligible study design	10.1002/erv.2837
In systemic sclerosis patients the anxiety disorder and Raynaud’s phenomenon are increased during lock down period for COVID-19 pandemic	Gigante et al.	2021	Ineligible study design	10.1007/s11739-020-02557-z
Depressive symptoms among adults in 2018–2019 and during the 2020 COVID-19 pandemic in Italy	Gigantesco et al.	2022	Ineligible study design	10.1016/j.jad.2022.04.131
Evidencing the influence of pre-pandemic sports participation and substance misuse on physical activity during the COVID-19 lockdown: a prospective analysis among older adolescents	Gilic et al.	2021	Ineligible outcomes	10.13075/IJOMEH.1896.01733
Lockdown dreams: dream content and emotions during the COVID-19 pandemic in an Italian sample	Giovanardi et al.	2021	Ineligible outcomes	10.1037/pap0000385
Emotional impact of COVID-19 lockdown among the Spanish population	Gismero-González et al.	2020	Ineligible study design	10.3389/fpsyg.2020.616978
COVID-19 and lockdown: impact on mental health among the residents of Assam, India	Gogoi et al.	2020	Ineligible study design	10.1177/1010539520962952
Examining the impact of the COVID-19 pandemic on youth alcohol consumption: longitudinal changes from pre-to intra-pandemic drinking in the COMPASS study	Gohari et al.	2022	Ineligible outcomes	10.1016/j.jadohealth.2022.07.007
Caregiving of older persons during the COVID-19 pandemic in the Russian arctic province: challenges and practice	Golubeva et al.	2022	Ineligible study design	10.3390/ijerph19052775
Sleep quality, depression and anxiety in a community sample of Havana, Cuba, during the 2020 COVID-19 pandemic	González et al.	2021	Ineligible study design	10.1093/sleep/zsab072.696
A comparative cross-sectional study of the consequences of the COVID-19 lockdown on women’s health behaviors in Spain	González-Calderón et al.	2022	Ineligible study design	10.3390/nu14040846
Deterioration of mental health and insufficient COVID-19 information among disadvantaged immigrants in the greater Paris area	Gosselin et al.	2021	Ineligible study design	10.1016/j.jpsychores.2021.110504
“A blessing and a curse”: work loss during coronavirus lockdown on short-term health changes via threat and recovery	Grandey et al.	2021	Ineligible study design	10.1037/ocp0000283
COVID-19 lockdown and consumption patterns among substance use disorder outpatients: a multicentre study	Grau-López et al.	2022	Ineligible outcomes	10.1159/000521425
The Influence of the COVID-19 pandemic on mental well-being and psychological distress: impact upon a single country	Gray et al.	2020	Ineligible outcomes	10.3389/fpsyt.2020.594115
The health impacts of a 4-month long community-wide COVID-19 lockdown: findings from a prospective longitudinal study in the state of Victoria, Australia	Griffiths et al.	2022	Ineligible outcomes	10.1371/journal.pone.0266650
How has COVID-19 lockdown impacted smoking? A thematic analysis of written accounts from UK smokers	Grogan et al.	2022	Ineligible study design	10.1080/08870446.2020.1862110
Monitoring the impact of COVID-19 pandemic on mental health: a public health challenge? Reflection on Italian data	Gualano et al.	2021	Others - letter to the editor	10.1007/s00127-020-01971-0
Changes in smoking and alcohol consumption during COVID-19-related lockdown: a cross-sectional study in France	Guignard et al.	2021	Ineligible study design	10.1093/eurpub/ckab054
How has the COVID-19 pandemic affected tobacco users in India: Lessons from an ongoing tobacco cessation program	Gupte et al.	2020	Ineligible study design	10.18332/tpc/127122
Examining children and adolescent mental health trajectories during the COVID‐19 pandemic: findings from a year of the Co‐SPACE study	Guzman et al.	2023	Ineligible outcomes	10.1002/jcv2.12153
Predictors of COVID-related changes in mental health in a South African sample of adolescents and young adults.	Haag et al.	2022	Ineligible study design	10.1080/13548506.2022.2108087
Association between mental health and physical activity levels in people with Parkinson’s disease during the COVID-19 pandemic: an observational cross-sectional survey in Brazil.	Haas et al.	2022	Ineligible intervention	10.1007/s11332-021-00868-y
COVID-19 and psychosis, depression, obsession and quality of life in Lebanese patients with schizophrenia: any changes after 5 months of quarantine?	Haddad et al.	2022	Ineligible intervention	10.1186/s40359-022-00750-7
Adolescents’ symptoms of anxiety and depression before and during the COVID-19 outbreak – a prospective population-based study of teenagers in Norway.	Hafstad et al.	2021	Ineligible study design	10.1016/j.lanepe.2021.100093
Predictors of change in mental health during the COVID-19 pandemic	Haliwa et al.	2021	Ineligible intervention	10.1016/j.jad.2021.05.045
The impact of COVID-19 on sleep in autistic adults: longitudinal comparisons pre and during lockdown	Halstead et al.	2021	Ineligible study design	10.3389/fpsyt.2021.708339
Insomnia symptoms in the general population during the COVID-19 pandemic	Halsøy et al.	2021	Ineligible study design	10.3389/fpsyt.2021.762799
Immediate impact of stay-at-home orders to control COVID-19 transmission on socioeconomic conditions, food insecurity, mental health, and intimate partner violence in Bangladeshi women and their families: an interrupted time series	Hamadani et al.	2020	Ineligible study design	10.1016/S2214-109X(20)30366-1
The toll of a second lockdown: a longitudinal study	Hamama-Raz et al.	2021	Ineligible control	10.1016/j.jad.2021.06.080
Experiences of American older adults with pre-existing depression during the beginnings of the COVID-19 pandemic: a multicity, mixed-methods study	Hamm et al.	2020	Ineligible intervention	10.1016/j.jagp.2020.06.013
Item-level analysis of mental health symptom trajectories during the COVID-19 pandemic in the UK: associations with age, sex and pre-existing psychiatric conditions.	Hampshire et al.	2022	Ineligible outcomes	10.1016/j.comppsych.2022.152298
When social isolation is nothing new: a longitudinal study on psychological distress during COVID-19 among university students with and without preexisting mental health concerns.	Hamza et al.	2021	Ineligible study design	10.1037/cap0000255
Depression following COVID-19 lockdown in severely, moderately, and mildly impacted areas in China	Han et al.	2021	Ineligible study design	10.3389/fpsyt.2021.596872
Alcohol- and cigarette-use related behaviors during quarantine and physical distancing amid COVID-19 in Indonesia	Hanafi et al.	2021	Ineligible study design	10.3389/fpsyt.2021.622917
Covid-fatigued? A longitudinal study of Norwegian older adults’ psychosocial well-being before and during early and later stages of the COVID-19 pandemic	Hansen et al.	2022	Ineligible outcomes	10.1007/s10433-021-00648-0
The role of mindfulness and life satisfaction in psychological distress during the COVID-19 lockdown in New Zealand: a quasi-experimental study	Hartstone et al.	2021	Ineligible study design	10.1007/s12671-021-01731-4
The impact of COVID-19 lockdown on daily activities, cognitions, and stress in a lonely and distressed population: temporal dynamic network analysis	Haucke et al.	2022	Ineligible outcomes	10.2196/32598
Effects of COVID-19 lockdown on parental functioning in vulnerable families	Helland et al.	2021	Ineligible outcomes	10.1111/jomf.12789
Effects of the COVID-19 pandemic on the mental health of prisoners	Hewson et al.	2020	Others - comments	10.1016/S2215-0366(20)30241-8
The impact of the COVID-19 pandemic on functional and mental health outcomes after trauma	Heyman et al.	2022	Ineligible intervention	10.1016/J.AMJSURG.2022.03.012
Psychological stress associated with the COVID-19 pandemic in postpartum women in Yokohama, Japan	Hiiragi et al.	2021	Ineligible intervention	10.1111/jog.14776
COVID 19: impact of lock-down on mental health and tips to overcome	Hiremath et al.	2020	Others - narrative review	10.1016/J.AJP.2020.102088
The impact of the COVID-19 outbreak on mental wellbeing in children with a chronic condition compared to healthy peers	Hoefnagels et al.	2022	Ineligible outcomes	10.3390/ijerph19052953
Loneliness and social distancing during the COVID-19 pandemic: risk factors associations with psychopathology	Hoffart et al.	2020	Ineligible study design	10.3389/fpsyt.2020.589127
Longitudinal factors associated with increased alcohol consumption in adults during the COVID-19 pandemic	Holland et al.	2023	Ineligible outcomes	10.1080/00952990.2023.2176236
Adolescent and maternal anxiety symptoms decreased but depressive symptoms increased before to during COVID-19 lockdown	Hollenstein et al.	2021	Ineligible study design	10.1111/jora.12663
Loneliness, mental health, and substance use among US young adults during COVID-19	Horigian et al.	2021	Ineligible study design	10.1080/02791072.2020.1836435
Adolescents' longitudinal trajectories of mental health and loneliness: the impact of COVID-19 school closures	Houghton et al.	2022	Ineligible study design	10.1002/jad.12017
A longitudinal assessment of depression and anxiety in the Republic of Ireland before and during the COVID-19 pandemic	Hyland et al.	2021	Ineligible study design	10.1016/j.psychres.2021.113905
COVID-19 lockdown 2020 changed patterns of alcohol and cannabis use in Swiss elite athletes and bodybuilders: results from an online survey	Imboden et al.	2021	Ineligible study design	10.3389/fspor.2021.759335
Prevalence of anxiety and depression in patients with rheumatoid arthritis before and during the COVID-19 pandemic	Itaya et al.	2021	Ineligible intervention	10.1093/rheumatology/keab065
A comparative study of access to inpatient psychiatric treatment in a public mental health service in Melbourne during COVID-19	Itrat et al.	2020	Ineligible outcomes	10.4103/psychiatry.IndianJPsychiatry_852_20
Association of the COVID-19 lockdown with smoking, drinking and attempts to quit in England: an analysis of 2019–20 data	Jackson et al.	2021	Ineligible outcomes	10.1111/add.15295
Alcohol use and mental health during COVID-19 lockdown: a cross-sectional study in a sample of UK adults.	Jacob et al.	2021	Ineligible study design	10.1016/J.DRUGALCDEP.2020.108488
Emergency department visits for psychiatric care during the first lockdown in Melbourne	Jagadheesan et al.	2022	Ineligible outcomes	10.1177/10398562211037329
Mental ill-health during COVID-19 confinement	Jané-Llopis et al.	2021	Ineligible study design	10.1186/S12888-021-03191-5
Resilience of adolescents, though weakened during pandemic-related lockdown, serves as a protection against depression and sleep problems	Jiang et al.	2022	Ineligible outcomes	10.1080/13548506.2021.1990367
The impact of the initial and second national COVID-19 lockdowns on mental health in young people with and without pre-existing depressive symptoms	Joensen et al.	2022	Ineligible outcomes	10.1016/J.JPSYCHIRES.2022.03.001
Parenting in a pandemic: parental stress, anxiety and depression among parents during the government-initiated physical distancing measures following the first wave of COVID-19	Johnson et al.	2022	Ineligible study design	10.1002/smi.3120
Mental health and quality of life for people with rheumatoid arthritis or ankylosing spondylitis in Aotearoa New Zealand following the COVID-19 national lockdown	Johnstone et al.	2021	Ineligible study design	10.1007/s00296-021-04952-x
The beneficial effect of the first COVID-19 lockdown on undergraduate students of education: prospective cohort study	Joseph et al.	2022	Ineligible study design	10.2196/27286
Impact of COVID-19 pandemic and lockdown in a cohort of myasthenia gravis patients in India	Kalita et al.	2021	Ineligible study design	10.1016/J.CLINEURO.2021.106488
Longitudinal comparisons of mental health, burnout and well-being in patient-facing, non-patient-facing healthcare professionals and non-healthcare professionals during the COVID-19 pandemic: findings from the CoPE-HCP study	Kapil et al.	2022	Ineligible study design	10.1192/bjo.2022.579
Multinational dietary changes and anxiety during the coronavirus pandemic-findings from Israel	Kaufman-Shriqui et al.	2021	Ineligible study design	10.1186/S13584-021-00461-1
Estimated prevalence of and factors associated with clinically significant anxiety and depression among US adults during the first year of the COVID-19 pandemic	Kessler et al.	2022	Ineligible outcomes	10.1001/jamanetworkopen.2022.17223
Effect of COVID-19 lockdown on alcohol consumption in patients with pre-existing alcohol use disorder	Kim et al.	2020	Ineligible study design	10.1016/S2468-1253(20)30251-X
Impacts of coping mechanisms on nursing students’ mental health during covid-19 lockdown: a cross-sectional survey.	Kim et al.	2021	Ineligible study design	10.3390/nursrep11010004
Changes in physical activity and depressive symptoms during COVID-19 lockdown: United States adult age groups	Kim et al.	2022	Ineligible study design	10.3389/fpsyg.2022.769930
Evaluating the mental health impacts of the COVID-19 pandemic: perceived risk of COVID-19 infection and childhood trauma predict adult depressive symptoms in urban South Africa	Kim et al.	2022	Ineligible outcomes	10.1017/S0033291720003414
COVID-19, social restrictions, and mental distress among young people: a UK longitudinal, population-based study	Knowles et al.	2022	Ineligible outcomes	10.1111/jcpp.13586
Sleep quality during the COVID-19 pandemic: not one size fits all	Kocevska et al.	2020	Ineligible study design	10.1016/j.sleep.2020.09.029
Lockdown of 1.3 billion people in India during COVID-19 pandemic: a survey of its impact on mental health	Kochhar et al.	2020	Ineligible study design	10.1016/J.AJP.2020.102213
Impact of COVID-19 pandemic exacerbation of depressive symptoms for social frailty from the ORANGE registry	Kodama et al.	2022	Ineligible study design	10.3390/ijerph19020986
Did the general population in Germany drink more alcohol during the COVID-19 pandemic lockdown?	Koopmann et al.	2020	Others - letter to the editor	10.1093/alcalc/agaa058
The effects of the lockdown during the COVID-19 pandemic on alcohol and tobacco consumption behavior in Germany	Koopmann et al.	2021	Ineligible study design	10.1159/000515438
Psychological health of pregnant and postpartum women before and during the COVID-19 pandemic	Kuipers et al.	2022	Ineligible study design	10.1371/journal.pone.0267042
Low uptake of COVID-19 prevention behaviours and high socioeconomic impact of lockdown measures in South Asia: evidence from a large-scale multi-country surveillance programme	Kusuma et al.	2021	Ineligible study design	10.1016/j.ssmph.2021.100751
Mental health before and during the COVID-19 pandemic in two longitudinal UK population cohorts	Kwong et al.	2021	Ineligible study design	10.1192/bjp.2020.242
Socioeconomic and environmental factors associated with increased alcohol purchase and consumption in 38 countries during the COVID-19 pandemic	Kyaw et al.	2022	Ineligible study design	10.3389/fpsyt.2021.802037
Early postpartum stress, anxiety, depression, and resilience development among danish first-time mothers before and during first-wave COVID-19 pandemic	Ladekarl et al.	2021	Ineligible study design	10.3390/ijerph182211734
The impact of the COVID-19 pandemic on suicide rates in Hungary: an interrupted time-series analysis	Lantos et al.	2022	Ineligible intervention	10.1186/s12888-022-04322-2
Neuropsychiatric symptoms and quality of life in Spanish patients with Alzheimer’s disease during the COVID-19 lockdown	Lara et al.	2020	Ineligible study design	10.1111/ene.14339
The COVID-19 resource center is hosted on Elsevier Connect, the company's public news and information	Lebel et al.	2020	Ineligible study design	Not applicable
Changes in alcohol use as a function of psychological distress and social support following COVID-19 related university closings	Lechner et al.	2020	Ineligible study design	10.1016/j.addbeh.2020.106527
Posttraumatic stress disorder symptoms and coping with the lockdown among help-seeking veterans before and during the COVID-19 pandemic	Letica-Crepulja et al.	2021	Ineligible study design	10.3325/cmj.2021.62.241
The impact of sleep, physical activity and sedentary behaviour on symptoms of depression and anxiety before and during the COVID-19 pandemic in a sample of South African participants	Lewis et al.	2021	Ineligible study design	10.1038/s41598-021-02021-8
Novelty seeking and mental health in Chinese university students before, during, and after the COVID-19 pandemic lockdown: a longitudinal study	Li et al.	2020	Ineligible study design	10.3389/fpsyg.2020.600739
Anxiety and depression among general population in China at the peak of the COVID-19 epidemic	Li et al.	2020	Ineligible study design	10.1002/wps.20758
Self-reported hearing difficulties are associated with loneliness, depression and cognitive dysfunction during the COVID-19 pandemic	Littlejohn et al.	2022	Ineligible study design	10.1080/14992027.2021.1894492
Effect of coronavirus disease 2019 on the psychology and behavior of patients on methadone maintenance treatment in Wuhan, China: a clinical observational study	Liu et al.	2021	Ineligible study design	10.3389/fpsyt.2021.653662
Hidden in plain sight? Men's coping patterns and psychological distress before and during the COVID-19 pandemic	Livingston et al.	2022	Ineligible study design	10.3389/fpsyt.2021.772942
Pandemic-associated mental health changes in youth with neuroinflammatory disorders	Logan et al.	2022	Ineligible study design	10.1016/j.msard.2021.103468
Psychological distress associated with the COVID-19 pandemic and suppression measures during the first wave in Belgium	Lorant et al.	2021	Ineligible study design	10.1186/s12888-021-03109-1
The first wave of COVID-19 and concurrent social restrictions were not associated with a negative impact on mental health and psychiatric well-being	Love et al.	2022	Ineligible intervention	10.1111/joim.13461
Emotion regulation and psychological and physical health during a nationwide COVID-19 lockdown	Low et al.	2021	Ineligible study design	10.1037/emo0001046
Mental health of new undergraduate students before and after COVID-19 in China	Lu et al.	2021	No appropriate data	10.1038/s41598-021-98140-3
COVID-19 pandemic effects in people with autism spectrum disorder and their caregivers: evaluation of social distancing and lockdown impact on mental health and general status	Lugo-Marín et al.	2021	Ineligible study design	10.1016/J.RASD.2021.101757
The impact of lockdown during the COVID-19 pandemic on mental and social health of children and adolescents	Luijten et al.	2021	No appropriate data	10.1007/s11136-021-02861-x
Changes in alcohol-related behaviors and quality of life during the COVID-19 pandemic: impact of alcohol use disorder diagnosis and treatment history	Luk et al.	2023	Ineligible intervention	10.4088/JCP.22br14462
Mental well-being of university students in social isolation	Lukacs et al.	2021	Ineligible study design	10.1027/2512-8442/a000065
Influence of social isolation caused by coronavirus disease 2019 (COVID-19) on the psychological characteristics of hospitalized schizophrenia patients: a case-control study	Ma et al.	2020	Ineligible population	10.1038/s41398-020-01098-5
Children and adolescents’ psychological well-being became worse in heavily hit Chinese provinces during the COVID-19 epidemic	Ma et al.	2021	Ineligible design	10.20900/jpbs.20210020
Depression in the pediatric otolaryngology clinic setting	MacDonald et al.	2022	Ineligible study design	10.1002/lary.29856
Risk and protective factors for prospective changes in adolescent mental health during the COVID-19 pandemic	Magson et al.	2021	Ineligible study design	10.1007/s10964-020-01332-9
Safe in my heart: resting heart rate variability longitudinally predicts emotion regulation, worry, and sense of safeness during COVID-19 lockdown	Makovac et al.	2022	Ineligible outcomes	10.1080/10253890.2021.1999408
COVID-19 quarantine-related mental health symptoms and their correlates among mothers: a cross sectional study	Malkawi et al.	2021	Ineligible study design	10.1007/s10995-020-03034-x
Impact of lockdown due to COVID-19 pandemic in changes of prevalence of predictive psychiatric disorders among children and adolescents in Bangladesh	Mallik et al.	2021	Ineligible outcomes	10.1016/j.ajp.2021.102554
The impact of lockdown during SARS-CoV-2 outbreak on behavioral and psychological symptoms of dementia	Manini et al.	2021	Ineligible study design	10.1007/s10072-020-05035-8
Convicted drinking and driving offenders: comparing alcohol use before and after the pandemic outbreak	Manning et al.	2021	Ineligible study design	10.1111/acer.14613
Indirect acute effects of the COVID-19 pandemic on physical and mental health in the UK: a population-based study	Mansfield et al.	2021	Ineligible outcomes	10.1016/S2589-7500(21)00017-0
Study of resilience and loneliness in youth (18–25 years old) during the COVID-19 pandemic lockdown measures	Marchini et al.	2021	Ineligible outcomes	10.1002/jcop.22473
Impact of COVID-19 lockdown on sleep quality in university students and administration staff	Marelli et al.	2021	Ineligible study design	10.1007/s00415-020-10056-6
The impact of the COVID-19 pandemic on suicide mortality in Spain: differences by sex and age	Martínez-Alés et al.	2023	Ineligible study design	10.1016/j.jad.2023.02.115
Psychoactive substance use and its relationship to stress, emotional state, depressive symptomatology, and perceived threat during the COVID-19 pandemic in Mexico	Martínez-Vélez et al.	2021	Ineligible study design	10.3389/fpubh.2021.709410
Alcohol consumption and COVID-19 in Europe: how the pandemic hit the weak	Matone et al.	2022	Ineligible study design	10.4415/ANN_22_01_02
The analysis of alcohol consumption during the severe acute respiratory syndrome coronavirus 2 Italian lockdown	Mazzarella et al.	2022	Ineligible study design	10.23736/S0026-4806.21.07354-7
Depression, anxiety and suicidal behaviour among college students: comparisons pre-COVID-19 and during the pandemic	McLafferty et al.	2021	Ineligible study design	10.1016/J.PSYCOM.2021.100012
Depression, environmental reward, coping motives and alcohol consumption during the COVID-19 pandemic	McPhee et al.	2020	Ineligible study design	10.3389/fpsyt.2020.574676
The COVID-19 pandemic in Italy: depressive symptoms immediately before and after the first lockdown	Medda et al.	2022	Ineligible study design	10.1016/J.JAD.2021.10.129
Association between COVID-19-related loneliness or worry and symptoms of anxiety and depression among first-year college students	Mehus et al.	2023	Ineligible study design	10.1080/07448481.2021.1942009
A longitudinal study on the COVID-19 pandemic and its divergent effects on social participation and mental health across different study groups with and without mental disorders	Mergel et al.	2021	Ineligible study design	10.1007/s00127-021-02025-9
Mood and changes in alcohol consumption in young adults during covid-19 lockdown: a model explaining associations with perceived immune fitness and experiencing covid-19 symptoms	Merlo et al.	2021	Ineligible study design	10.3390/ijerph181910028
Changes in cannabis consumption among college students during COVID-19	Merrill et al.	2022	Ineligible outcomes	10.15288/jsad.2022.83.55
Changes in cannabis use and associated correlates during France’s first COVID-19 lockdown in daily cannabis users: results from a large community-based online survey	Mezaache et al.	2022	Ineligible study design	10.1186/s12954-022-00611-x
Adolescent drug use before and during U.S. national COVID-19 social distancing policies	Miech et al.	2021	Ineligible outcomes	10.1016/j.drugalcdep.2021.108822
Cannabis use during the early COVID-19 pandemic: use patterns, predictors, and subjective experiences	Mielau et al.	2023	Ineligible study design	10.3389/fpsyt.2022.1037451
Is talk cheap? Correspondence between self-attributions about changes in drinking and longitudinal changes in drinking during the 2019 coronavirus pandemic	Minhas et al.	2021	Ineligible study design	10.1111/acer.14724
Determinants and predictors of mental health during and after COVID-19 lockdown among university students in Malaysia	Mir et al.	2023	Ineligible intervention	10.1371/journal.pone.0280562
Assessment of level of perceived stress and sources of stress among dental professionals before and during the COVID-19 outbreak	Mishra et al.	2020	Ineligible outcomes	10.4103/JISPCD.JISPCD_340_20
Epidemiology of suicide in Western Odisha during COVID pandemic: a cross-sectional analysis	Mishra et al.	2022	Ineligible study design	10.7759/cureus.21438
Disentangling the root causes of COVID-19 related increases in alcohol consumption	Molsberry et al.	2021	Others - commentary	10.1080/00952990.2021.1881532
Mental health emergencies and COVID-19: the impact of ‘lockdown’ in the East Midlands of the UK	Moore et al.	2021	Ineligible outcomes	10.1192/bjo.2021.973
Mental health and life satisfaction among 10–11-year-olds in Wales, before and one year after onset of the COVID-19 pandemic	Moore et al.	2022	Ineligible outcomes	10.1186/s12889-022-12752-6
Psychological effects of the COVID-19 lockdown on children and families in the UK	Morgül et al.	2020	Ineligible outcomes	10.21134/rpcna.2020.mon.2049
Prevalent, incident, and persistent insomnia in a population-based cohort tested before (2018) and during the first-wave of COVID-19 pandemic (2020)	Morin et al.	2022	Ineligible study design	10.1093/sleep/zsab258
COVID shelter in place orders and mental health outcomes among college undergraduates	Morris et al.	2021	Ineligible study design	10.1080/07448481.2021.1978459
Psychological distress and tobacco use among hospital workers during COVID-19	Mounir et al.	2021	Ineligible study design	10.3389/fpsyt.2021.701810
Alcohol use in self-isolation during the COVID-19 pandemic: a cross-sectional survey in Brazil	Moura et al.	2023	Ineligible study design	10.47626/2237-6089-2021-0337
Mental health profiles in a sample of Moroccan high school students: comparison before and during the COVID-19 pandemic	Mzadi et al.	2022	Ineligible study design	10.3389/fpsyt.2021.752539
Effects of quarantine due to the COVID-19 on sleep time, anxiety, and physical activity in adult population: a longitudinal study in Kerman, southeastern Iran	Najafipour et al.	2021	Ineligible study design	10.22062/jkmu.2021.91661
Adolescent carers' psychological symptoms and mental well-being during the COVID-19 pandemic: longitudinal study using data from the UK Millennium cohort study	Nakanishi et al.	2022	Ineligible outcomes	10.1016/j.jadohealth.2022.01.228
Increased prevalence of gastrointestinal symptoms and disorders of gut-brain interaction during the COVID-19 pandemic: an Internet-based survey	Nakov et al.	2022	Ineligible outcomes	10.1111/nmo.14197
Impact of the COVID-19 pandemic on inflammatory bowel disease: the role of emotional stress and social isolation	Nass et al.	2022	Ineligible outcomes	10.1002/smi.3080
Health behaviour change during the UK COVID-19 lockdown: findings from the first wave of the C-19 health behaviour and well-being daily tracker study	Naughton et al.	2021	Ineligible study design	10.1111/bjhp.12500
Increase of depressive symptoms among adolescents during the first COVID-19 lockdown in Germany: results from the German family panel pairfam	Naumann et al.	2021	Ineligible study design	10.1007/s00103-021-03451-5
Alcohol use in Australia during the early days of the COVID-19 pandemic: initial results from the COLLATE project	Neill et al.	2020	Ineligible study design	10.1111/pcn.13099
Mental health and health behaviours before and during the initial phase of the COVID-19 lockdown: longitudinal analyses of the UK Household Longitudinal Study	Niedzwiedz et al.	2021	No appropriate data	10.1136/jech-2020-215060
Substances use between early and later stages of the COVID‑19 pandemic in Israel	Noach et al.	2021	Ineligible control	10.1186/s13584-021-00484-8
Effects of restraining measures due to COVID-19: pre- and post-lockdown cognitive status and mental health	Nogueira et al.	2021	Ineligible study design	10.1007/s12144-021-01747-y
Risk factors underlying COVID-19 lockdown-induced mental distress	Novotný et al.	2020	Ineligible study design	10.3389/fpsyt.2020.603014
The longitudinal effect of COVID-19 infections and lockdown on mental health and the protective effect of neighbourhood social relations	O'Donnell et al.	2022	Ineligible control	10.1016/J.SOCSCIMED.2022.114821
Child suicide rates during the COVID-19 pandemic in England	Odd et al.	2021	Ineligible study design	10.1016/J.JADR.2021.100273
Prevalence of depression and its relation to quality of life during the initial period of COVID-19 pandemic: a cross-sectional study on Turkish society and suggestions on potential solutions	Okudan et al.	2021	Ineligible study design	10.23736/S2724-6612.20.02118-4
Impact of COVID-19 lockdown on symptoms in patients with functional gastrointestinal disorders: Relationship with anxiety and perceived stress	Oliviero et al.	2021	Ineligible outcomes	10.1111/nmo.14092
Effects of COVID-19-related stay-at-home order on neuropsychophysiological response to urban spaces: beneficial role of exposure to nature?	Olszewska-Guizzo et al.	2021	Ineligible study design	10.1016/j.jenvp.2021.101590
Psychological and sexual health during the COVID-19 pandemic in Egypt: are women suffering more?	Omar et al.	2021	Ineligible outcomes	10.1016/j.esxm.2020.100295
How much of an impact did COVID-19 self-isolation measures have on mental health?	Omiya et al.	2020	Ineligible outcomes	10.1016/J.AJP.2020.102445
A 6-month follow-up study on worry and its impact on well-being during the first wave of COVID-19 pandemic in an Italian sample	Ongaro et al.	2021	Ineligible control	10.3389/fpsyg.2021.703214
Alcohol use cravings as a mediator between associated risk factors on increased alcohol use among youth adults in New York during the COVID-19 pandemic	Opara et al.	2021	Ineligible study design	10.1080/07347324.2021.1950091
Mental health in the post-lockdown pandemic phase: relief or exacerbation of psychological distress? A cross-sectional study in the general population in Italy	Orfei et al.	2022	Ineligible study design	10.1016/j.actpsy.2022.103555
Mental Health, resilience, and religiosity in the elderly under COVID-19 quarantine in Qatar	Ouanes et al.	2021	Ineligible intervention	10.1016/j.archger.2021.104457
Mental well-being during stages of COVID-19 lockdown among pregnant women and new mothers	Overbeck et al.	2022	No appropriate data	10.1186/s12884-021-04374-4
Stress, anxiety, and depression levels in the initial stage of the COVID-19 outbreak in a population sample in the northern Spain	Ozamiz-Etxebarria et al.	2020	Ineligible study design	10.1590/0102-311X00054020
Coronavirus pandemic: mood statuses of renal transplant recipients during social isolation and lockdown periods	Ozcan et al.	2021	Ineligible intervention	10.6002/ect.2020.0488
Does the progression of the COVID-19 pandemic have an influence on the mental health and well-being of young people? A cross-sectional multicenter study	Özlü-Erkilic et al.	2021	Ineligible control	10.3390/ijerph182312795
Impact of mental health on disease activity in mastocytosis during COVID-19 pandemic	Öztop et al.	2022	Ineligible control	10.1016/J.ALIT.2021.08.002
Changes in the frequency and pattern of drugs detected among suspected drug users during the COVID-19 pandemic in Turkey	Öztürk et al.	2022	Ineligible outcomes	10.1007/s00414-022-02794-1
Alcohol consumption changes during the first COVID-19 lockdown: an online population survey in a convenience sample of French-speaking Belgian residents	Pabst et al.	2021	Ineligible control	10.1016/j.psychres.2021.113938
Alcohol consumption changes following COVID-19 lockdown among French-speaking Belgian individuals at risk for alcohol use disorder	Pabst et al.	2021	Ineligible control	10.1016/J.PNPBP.2021.110282
Impact of COVID-19 physical distancing policies on incidence of intentional self-harm in Western Sydney	Page et al.	2021	Ineligible outcomes	10.1177/10398562211010808
Shifts in drug use behavior among electronic dance music partygoers in New York during COVID-19 social istancing	Palamar et al.	2021	Ineligible study design	10.1080/10826084.2020.1857408
Beyond lockdown: the potential side effects of the sSARS-CoV-2 pandemic on public health	Paltrinieri et al.	2021	Ineligible study design	10.3390/nu13051600
The mental health impact of the COVID-19 pandemic on people with and without depressive, anxiety, or obsessive-compulsive disorders: a longitudinal study of three Dutch case-control cohorts	Pan et al.	2021	No appropriate data	10.1016/S2215-0366(20)30491-0
Changes in alcohol use habits in the general population, during the COVID-19 lockdown in gGeece	Panagiotidis et al.	2020	Ineligible study design	10.1093/ALCALC/AGAA092
Psychiatric hospitalization during the two SARS-CoV-2 pandemic waves: new warnings for acute psychotic episodes and suicidal behaviors	Panariello et al.	2021	Ineligible outcomes	10.5498/wjgpt.v11.i11.1095
Psychological impact of mass quarantine on population during pandemics-the COVID-19 lock-down (COLD) study	Pandey et al.	2020	Ineligible control	10.1371/journal.pone.0240501
Relationships between psychopathology, psychological process variables, and sociodemographic variables and comparison of quarantined and non-quarantined groups of malaysian university students in the covid-19 pandemic	Pang et al.	2021	Ineligible study design	10.3390/ijerph18189656
Were self-described introverts "immune" to increased drug use and entrapment during the pandemic?	Panlilio et al.	2022	Ineligible study design	10.1016/J.DADR.2022.100024
COVID-19 related distress is associated with alcohol problems, social media and food addiction symptoms: insights from the Italian experience during the lockdown	Panno et al.	2020	Ineligible study design	10.3389/fpsyt.2020.577135
Suicidal ideation during COVID-19 lockdown in Greece: prevalence in the community, risk and protective factors	Papadopoulou et al.	2021	Ineligible study design	10.1016/j.psychres.2021.113713
Pain in chronic pancreatitis during the COVID-19 lockdown: has it given us a new dimension for treatment?	Parasar et al.	2021	Ineligible study design	10.7759/cureus.13423
Risk for probable post-partum depression among women during the COVID-19 pandemic	Pariente et al.	2020	No appropriate data	10.1007/s00737-020-01075-3
Impact of the COVID-19 pandemic on the lifestyle, mental health, and quality of life of adults in South Korea	Park et al.	2021	Ineligible study design	10.1371/journal.pone.0247970
Differences in multi-faceted lifestyles in response to the covid-19 pandemic and their association with depression and quality of life of older adults in South Korea: a cross-sectional study	Park et al.	2021	Ineligible intervention	10.3390/nu13114124
Risk factors for prospective increase in psychological stress during COVID-19 lockdown in a representative sample of adolescents and their parents	Paschke et al.	2021	Ineligible outcomes	10.1192/bjo.2021.49
COVID-19 pandemic: 1-year follow-Up in children and adolescents with neuropsychiatric disorders	Pastorino et al.	2023	Ineligible study design	10.3390/ijerph20053924
Psychological distress before and during the COVID-19 pandemic among adults in the United Kingdom based on coordinated analyses of 11 longitudinal studies	Patel et al.	2022	Others - narrative reveiw	10.1001/jamanetworkopen.2022.7629
Using substances to cope with the COVID-19 pandemic: U.S. national data at age 19 years	Patrick et al.	2022	Ineligible outcomes	10.1016/J.JADOHEALTH.2021.11.006
Alcohol use and the COVID-19 pandemic: historical trends in drinking, contexts, and reasons for use among U.S. adults	Patrick et al.	2022	Ineligible outcomes	10.1016/j.socscimed.2022.114887
Age- and sex-varying associations between depressive symptoms and substance use from modal ages 35 to 55 in a national sample of U.S. adults	Patrick et al.	2023	Ineligible intervention	10.1007/s11121-023-01491-8
Time trends in mental health indicators during the initial 16 months of the COVID-19 pandemic in Denmark	Pedersen et al.	2022	Ineligible control	10.1186/s12888-021-03655-8
Early adolescent substance use before and during the COVID-19 pandemic: a longitudinal survey in the ABCD study cohort	Pelham et al.	2021	Ineligible population	10.1016/j.jadohealth.2021.06.015
Coronial postmortem reports and indirect COVID-19 pandemic-related mortality	Pell et al.	2022	Ineligible study design	10.1136/jclinpath-2021-208003
COVID-19, impacts on the mental health of people suffering from anxiety and depression	Pellegrina et al.	2020	Ineligible study design	10.1016/S0241-6972(20)30123-7
Change in youth mental health during the COVID-19 pandemic in a majority Hispanic/Latinx US sample	Penner et al.	2021	Ineligible outcomes	10.1016/j.jaac.2020.12.027
Greatest changes in objective sleep architecture during COVID-19 lockdown in night owls with increased REM sleep	Pépin et al.	2021	Ineligible study design	10.1093/sleep/zsab075
Impact of the COVID-19 lockdown on a long-term care facility: the role of social contact	Pereiro et al.	2021	Ineligible study design	10.3390/brainsci11080986
Effects of a pandemic and isolation on alcohol and psychoactive medication use in a population of rehabilitation and pain patients	Pesce et al.	2021	Ineligible outcomes	
Iranian older adult's mental wellbeing during the COVID-19 epidemic	Peyman et al.	2020	Ineligible study design	10.1016/J.AJP.2020.102331
Cohort profile: the UK COVID-19 Public Experiences (COPE) prospective longitudinal mixed-methods study of health and well-being during the SARSCoV2 coronavirus pandemic	Phillips et al.	2021	Ineligible outcomes	10.1371/journal.pone.0258484
Sociodemographic and lifestyle predictors of mental health adaptability during COVID-19 compulsory confinement: a longitudinal study in the Portuguese population	Picó-Pérez et al.	2021	Ineligible control	10.1016/J.JAD.2021.08.150
Corrigendum to "The effect of age, gender, income, work, and physical activity on mental health during coronavirus disease (COVID-19) lockdown in Austria" [Journal of Psychosomatic Research 136 (2020) 110186]	Pieh et al.	2020	Ineligible study design	10.1016/J.JPSYCHORES.2020.110278
Mental health during COVID-19 lockdown in the United Kingdom	Pieh et al.	2021	Ineligible study design	10.1097/PSY.0000000000000871
Comparing mental health during the COVID-19 lockdown and 6 months after the lockdown in Austria: a longitudinal study	Pieh et al.	2021	Ineligible control	10.3389/fpsyt.2021.625973
Assessment of mental health of high school students during social distancing and remote schooling during the COVID-19 pandemic in Austria	Pieh et al.	2021	Ineligible study design	10.1001/jamanetworkopen.2021.14866
Mental health before and during the COVID-19 pandemic: a longitudinal probability sample survey of the UK population	Pierce et al.	2020	Ineligible outcomes	10.1016/S2215-0366(20)30308-4
Mental health responses to the COVID-19 pandemic: a latent class trajectory analysis using longitudinal UK data	Pierce et al.	2021	Ineligible outcomes	10.1016/S2215-0366(21)00151-6
Association of symptoms of posttraumatic stress disorder with posttraumatic psychological growth among US veterans during the COVID-19 pandemic	Pietrzak et al.	2021	Ineligible outcomes	10.1001/jamanetworkopen.2021.4972
Suicide trends in the early months of the COVID-19 pandemic: an interrupted time-series analysis of preliminary data from 21 countries	Pirkis et al.	2021	Ineligible intervention	10.1016/S2215-0366(21)00091-2
Suicide numbers during the first 9-15 months of the COVID-19 pandemic compared with pre-existing trends: an interrupted time series analysis in 33 countries	Pirkis et al.	2022	Ineligible intervention	10.1016/j.eclinm.2022.101573
Longitudinal evaluation of the psychological impact of the COVID-19 crisis in Spain	Planchuelo-Gómez et al.	2020	Ineligible study design	10.1016/j.jad.2020.09.018
Psychological effects of social isolation during the COVID-19 pandemic 2020	Plangger et al.	2022	Ineligible study design	10.1024/1662-9647/a000283
Impact of the COVID-19 pandemic on patients with pre-existing anxiety disorders attending secondary care	Plunkett et al.	2021	Ineligible study design	10.1017/ipm.2020.75
How are you coping with the COVID-19 pandemic? Survey of undergraduate dental students’ well-being during an unexpected global event	Poma et al.	2022	Ineligible study design	10.1111/eje.12721
Evaluating changes in student health, wellbeing and social circumstances before and during COVID-19 pandemic restrictions in Australia	Post et al.	2021	Ineligible outcomes	10.7717/peerj.12078
Depression in and after COVID-19 lockdown in Austria and the role of stress and loneliness in lockdown: a longitudinal study	Probst et al.	2020	Ineligible control	10.1016/J.JAD.2020.09.047
Change in health, wellbeing and physical activity levels during the COVID-19 pandemic: a longitudinal cohort of parkrun participants in the United Kingdom	Quirk et al.	2023	Ineligible outcomes	10.1093/heapro/daac012
Maternal depressive and anxiety symptoms before and during the COVID-19 pandemic in Canada: a longitudinal analysis	Racine et al.	2021	Ineligible study design	10.1016/S2215-0366(21)00074-2
Does a ban on liquor sales benefit alcohol dependence patients? A study on usage and procurement of alcohol during the COVID-19 lockdown	Rajendran et al.	2023	Ineligible study design	10.4103/kleuhsj.kleuhsj_489_22
Impact of the COVID-19 pandemic on the mental health of the general population: reflections and proposals	Ramírez et al.	2021	Others - editorial	10.1016/j.aprim.2021.102143
A longitudinal study of mental health before and during COVID-19 lockdown in the French population	Ramiz et al.	2021	Ineligible study design	10.1186/s12992-021-00682-8
Elderly suicides in India: an emerging concern during COVID-19 pandemic	Rana et al.	2020	Ineligible outcomes	10.1017/S1041610220001052
Risky alcohol consumption in older people before and during the COVID-19 pandemic in the United Kingdom	Rao et al.	2022	Ineligible study design	10.1080/14659891.2021.1916851
Factors associated with changes in consumption among smokers and alcohol drinkers during the COVID-19 'lockdown' period	Reynolds et al.	2021	Ineligible study design	10.1093/eurpub/ckab050
Longitudinal examination of COVID-19 public health measures on mental health for rural patients with serious mental illness	Riblet et al.	2021	Ineligible outcomes	10.1093/milmed/usaa559
Coping with COVID: risk and resilience factors for mental health in a German representative panel study	Riepenhausen et al.	2023	Ineligible outcomes	10.1017/S0033291722000563
Exploring mental health during the initial COVID-19 lockdown in Mumbai: serendipity for some women	Roberts et al.	2021	Ineligible study design	10.3390/ijerph182312542
The medium-term consequences of a COVID-19 lockdown on lifestyle among Spanish older people with hypertension, pulmonary disease, cardiovascular disease, musculoskeletal disease, depression, and cancer	Rodríguez-Gómez et al.	2022	Ineligible control	10.4178/epih.e2022026
Adolescents' perceived socio-emotional impact of COVID-19 and implications for mental health: results from a U.S.-based mixed-methods study	Rogers et al.	2021	Ineligible study design	10.1016/j.jadohealth.2020.09.039
Consumption of alcohol, cannabis, and tobacco in a cohort of adolescents before and during COVID-19 confinement	Rogés et al.	2021	Ineligible outcomes	10.3390/ijerph18157849
Lockdown duration and training intensity affect sleep behavior in an international sample of 1,454 elite athletes	Romdhani et al.	2022	Ineligible study design	10.3389/fphys.2022.904778
Impact of the COVID-19 pandemic and lockdown on the clinical response to dupilumab treatment and the psychological status of non-infected atopic patients	Rovati et al.	2021	Ineligible study design	10.1684/ejd.2021.4135
Anxiety and motivation to return to sport during the French COVID-19 lockdown	Ruffault et al.	2020	Ineligible outcomes	10.3389/fpsyg.2020.610882
The role of online social comparison as a protective factor for psychological wellbeing: a longitudinal study during the COVID-19 quarantine	Ruggieri et al.	2021	Ineligible study design	10.1016/J.PAID.2020.110486
Changes in mental health across the COVID-19 pandemic for local and international university students in Australia: a cohort study	Russell et al.	2023	Ineligible study design	10.1186/s40359-023-01075-9
Health behaviors and subsequent mental health problems during the COVID-19 pandemic: a longitudinal analysis of adults in the UK	Russell et al.	2023	Ineligible outcomes	10.3389/fpubh.2022.1064677
Risk factors for depression during the COVID-19 pandemic: a longitudinal study in middle-aged and older adults	Rutland-Lawes et al.	2021	Ineligible study design	10.1192/bjo.2021.997
Behavioral and psychological correlates of well-being during COVID-19	Ryerson et al.	2022	Ineligible study design	10.1177/0033294120978160
Psychiatric admissions, referrals, and suicidal behavior before and during the COVID-19 pandemic in Denmark: a time-trend study	Rømer et al.	2021	Ineligible outcomes	10.1111/acps.13369
Minimal impact of COVID-19 pandemic on the mental health and wellbeing of people living with dementia: analysis of matched longitudinal data from the IDEAL study	Sabatini et al.	2022	Ineligible outcomes	10.3389/fpsyt.2022.849808
Never too late to plan: “refocus on planning” as an effective way to lower symptoms and difficulties in emotion regulation during the COVID-19 first lockdown	Sacchi et al.	2021	Ineligible study design	10.1037/emo0001039
The immediate impact of lockdown measures on mental health and couples’ relationships during the COVID-19 pandemic - results of a representative population survey in Germany	Sachser et al.	2021	Ineligible outcomes	10.1016/j.socscimed.2021.113954
Risk for depressive symptoms among hospitalized women in high-risk pregnancy units during the covid-19 pandemic	Sade et al.	2020	No appropriate data	10.3390/jcm9082449
Mood and behaviors of adolescents with depression in a longitudinal study before and during the COVID-19 pandemic	Sadeghi et al.	2022	Ineligible study design	10.1016/j.jaac.2022.04.004
Changes in the clustering of health-related behaviors during the COVID-19 pandemic: examining predictors using latent transition analysis	Salazar-Fernández et al.	2022	Ineligible study design	10.1186/s12889-022-13854-x
Negative impact of COVID-19 pandemic on sleep quantitative parameters, quality, and circadian alignment: Implications for health and psychological well-being	Salehinejad et al.	2020	Ineligible study design	10.17179/excli2020-2831
Changes in mental health and well-being are associated with living arrangements with parents during COVID-19 among sexual minority young persons in the U.S.	Salerno et al.	2021	Ineligible outcomes	10.1037/sgd0000520
Changes in cannabis consumption during the global COVID-19 lockdown: the International COVISTRESS Study	Salles et al.	2021	Ineligible study design	10.3389/fpsyt.2021.689634
Assessing international alcohol consumption patterns during isolation from the COVID-19 pandemic using an online survey: highlighting negative emotionality mechanisms	Sallie et al.	2020	Ineligible study design	10.1136/bmjopen-2020-044276
Effects of COVID-19 lockdown on physical activity in coronary patients on a phase III cardiac rehabilitation program	Santaularia et al.	2021	Ineligible outcomes	10.1093/eurjpc/zwab061.324
Effect of COVID-19 confinement on the mental status of patients with systemic lupus erythematosus	Santos-Ruiz et al.	2021	Ineligible study design	10.1016/J.MEDCLE.2020.12.009
Pre-pandemic individual- and community-level social capital and depressive symptoms during COVID-19: A longitudinal study of Japanese older adults in 2019-21	Sato et al.	2022	Ineligible study design	10.1016/J.HEALTHPLACE.2022.102772
Trends in depression & anxiety symptom severity among mental health service attendees during the COVID-19 pandemic	Saunders et al.	2021	Ineligible study design	10.1016/J.JAD.2021.04.020
Mental health and movement behaviour during the COVID-19 pandemic in UK university students: Prospective cohort study	Savage et al.	2020	Ineligible outcomes	10.1016/j.mhpa.2020.100357
Mental health in clinically referred children and young people before and during the COVID-19 pandemic	Sayal et al.	2022	Ineligible outcomes	10.1007/s00787-022-02115-2
Embracing resilience in multiple sclerosis: a new perspective from COVID-19 pandemic	Sbragia et al.	2022	Ineligible study design	10.1080/13548506.2021.1916964
A longitudinal study of depression before, during, and following the COVID-19 nation-wide lockdown in Aotearoa New Zealand	Scarf et al.	2022	Ineligible study design	10.1177/10105395221074536
Distancing measures in COVID-19 pandemic: loneliness, more than physical isolation, affects health status and psycho-cognitive wellbeing in elderly patients with chronic obstructive pulmonary disease	Scarlata et al.	2021	Ineligible study design	10.1080/15412555.2021.1941834
COVID-19 and the subsequent lockdown modified dietary habits of almost half the population in an Italian sample	Scarmozzino et al.	2020	Ineligible study design	10.3390/foods9050675
Psychological burden during the COVID-19 pandemic in Germany	Schelhorn et al.	2021	Ineligible study design	10.3389/fpsyg.2021.640518
Effects of the COVID-19 pandemic Nationwide lockdown on mental health, environmental concern, and prejudice against other social groups	Schiller et al.	2022	Ineligible study design	10.1177/00139165211036991
The effect of environmental stressors on tinnitus: a prospective longitudinal study on the impact of the covid-19 pandemic.	Schlee et al.	2020	Ineligible study design	10.3390/jcm9092756
Changes in alcohol use during the COVID-19 pandemic: impact of the lockdown conditions and mental health factors	Schmits et al.	2022	Ineligible study design	10.1007/s11469-020-00432-8
Partners in lockdown: relationship stress in men and women during the COVID-19 pandemic	Schokkenbroek et al.	2021	Ineligible outcomes	10.1037/cfp0000172
Psychosocial and behavioral outcomes and transmission prevention behaviors: working during the coronavirus disease 2019 pandemic	Senerat et al.	2021	Ineligible outcomes	10.1016/j.mayocpiqo.2021.08.014
Psychiatric rehabilitation in Austria - a comparison of symptoms at admission before and during COVID-19 pandemic, as well as rehabilitation success	Senft et al.	2022	Ineligible study design	10.1055/a-1647-8566
Noise annoyance during COVID-19 lockdown: a research of public opinion before and during the pandemic.	Şentop et al.	2020	Ineligible study design	10.1121/10.0002667
Impact of social isolation due to COVID-19 on health in older people: mental and physical effects and recommendations	Sepulveda et al.	2020	Others - narrative review	10.3390/ijerph18094627
Neural responses to social reward predict depressive symptoms in adolescent girls during the COVID-19 pandemic	Sequeira et al.	2021	No appropriate data	10.1093/jpepsy/jsab037
The impact of the COVID-19 pandemic on the mental health of Portuguese university students	Sequeira et al.	2022	Ineligible outcomes	10.1111/inm.12999
Impact of COVID-19 lockdowns on mental health: evidence from a quasi-natural experiment in England and Scotland	Serrano-Alarcón et al.	2022	Ineligible outcomes	10.1002/hec.4453
Frequency of depressive symptoms in Syrian refugees and Turkish maintenance hemodialysis patients during COVID-19 pandemic	Sevinc et al.	2021	Ineligible study design	10.1371/journal.pone.0244347
Association of global cognitive function with psychological distress and adherence to public health recommendations during the coronavirus disease 2019 pandemic: the Women's Health Initiative	Shadyab et al.	2022	Ineligible outcomes	10.1093/GERONA/GLAC053
Mental-health before and during the COVID-19 pandemic in adults with neurodevelopmental disorders	Shakeshaft et al.	2023	No appropriate data	10.1016/j.jpsychires.2023.01.029
Impact of COVID-19 on loneliness, mental health, and health service utilisation: a prospective cohort study of older adults with multimorbidity in primary care	Shan et al.	2020	Ineligible design	10.3399/BJGP20X713021
Emotional distress in young adults during the COVID-19 pandemic: evidence of risk and resilience from a longitudinal cohort study	Shanahan et al.	2022	Ineligible outcomes	10.1017/S003329172000241X
Changes in substance use among young adults during a respiratory disease pandemic	Sharma et al.	2020	Ineligible study design	10.1177/2050312120965321
Gambling in COVID-19 lockdown in the UK: depression, stress, and anxiety	Sharman et al.	2021	Ineligible study design	10.3389/fpsyt.2021.621497
A longitudinal cohort study of youth mental health and substance use before and during the COVID-19 pandemic in Ontario, Canada: an exploratory analysis	Sheikhan et al.	2022	Ineligible outcomes	10.1177/07067437221097906
The impact of positive youth development attributes on posttraumatic stress disorder symptoms among Chinese adolescents under COVID-19	Shek et al.	2021	Ineligible outcomes	10.1016/j.jadohealth.2021.01.011
Mental disorders and emotional competence among Chinese adolescents before and during COVID-19 pandemic: a longitudinal mediation model	Shi et al.	2021	Ineligible study design	10.3389/fpubh.2021.767004
Impact of the COVID-19 pandemic on suicide and self-harm among patients presenting to the emergency department of a teaching hospital in Nepal	Shrestha et al.	2021	Ineligible study design	10.1371/journal.pone.0250706
Effects of the COVID-19 pandemic and nationwide lockdown on trust, attitudes toward government, and well-being	Sibley et al.	2020	Ineligible outcomes	10.1037/amp0000662
Top problems of adolescents and young adults with ADHD during the COVID-19 pandemic	Sibley et al.	2021	Ineligible outcomes	10.1016/J.JPSYCHIRES.2021.02.009
Biopsychosocial response to the COVID-19 lockdown in people with major depressive disorder and multiple sclerosis	Siddi et al.	2022	No appropriate data	10.3390/jcm11237163
Directional effects of social isolation and quality of life on anxiety levels among community-dwelling older adults during a COVID-19 lockdown	Siew et al.	2021	Ineligible study design	10.1016/j.jagp.2021.03.012
Threatening increase in alcohol consumption in physicians quarantined due to coronavirus outbreak in Poland: the ALCOVID survey	Silczuk et al.	2020	Ineligible intervention	10.1093/pubmed/fdaa110
Coping with the COVID-19 pandemic: perceived changes in psychological vulnerability, resilience and social cohesion before, during and after lockdown.	Silveira et al.	2022	Ineligible study design	10.3390/ijerph19063290
Postpartum mood among universally screened high and low socioeconomic status patients during COVID-19 social restrictions in New York City	Silverman et al.	2020	Ineligible study design	10.1038/s41598-020-79564-9
Early pregnancy mood before and during COVID-19 community restrictions among women of low socioeconomic status in New York City: a preliminary study	Silverman et al.	2020	Ineligible study design	10.1007/S00737-020-01061-9
The psychological impact of SARS: a matter of heart and mind	Sim et al.	2004	Others - commentary	10.1503/cmaj.1032003
Mental health impact on at-risk high-level athletes during COVID-19 lockdown: a pre-, during and post-lockdown longitudinal cohort study of adjustment disorder	Simons et al.	2021	Ineligible outcomes	10.1016/J.JSAMS.2020.12.012
Parental burnout during the COVID-19 pandemic	Skjerdingstad et al.	2022	Ineligible study design	10.1111/FAMP.12740
Change in psychological burden during the COVID-19 pandemic in Germany: fears, individual behavior, and the relevance of information and trust in governmental institutions	Skoda et al.	2021	Ineligible study design	10.1007/s00103-021-03278-0
COVID-19 and shielding: experiences of UK patients with lupus and related diseases.	Sloan et al.	2021	Ineligible outcomes	10.1093/rap/rkab003
The impact of Covid-19 restrictions on depressive symptoms in low-risk and high-risk pregnant women: a cross-sectional study before and during pandemic	Smorti et al.	2022	Ineligible study design	10.1186/s12884-022-04515-3
Becoming a mother during the COVID-19 national lockdown in Italy: Issues linked to the wellbeing of pregnant women	Smorti et al.	2022	Ineligible study design	10.1002/ijop.12806
Effects of COVID-19 home confinement on mental health in individuals with increased risk of Alzheimer's disease	Soldevila-Domenech et al.	2021	Ineligible study design	10.3233/JAD-201408
The effects of COVID-19 lockdown 1.0 on working patterns, income, and wellbeing among performing arts professionals in the United Kingdom (April–June 2020)	Spiro et al.	2021	Ineligible study design	10.3389/fpsyg.2020.594086
Parents’ perceived impact of the societal lockdown of COVID-19 on family well-being and on the emotional and behavioral state of walloon belgian children aged 4 to 13 years: an exploratory study	Stassart et al.	2021	Ineligible study design	10.5334/pb.1059
Altered alcohol consumption during COVID-19 pandemic lockdown	Steffen et al.	2021	Ineligible study design	10.1186/s12937-021-00699-0
Stop talking about it already! Co-ruminating and social media focused on COVID-19 was associated with heightened state anxiety, depressive symptoms, and perceived changes in health anxiety during Spring 2020	Stone et al.	2022	Ineligible study design	10.1186/s40359-022-00734-7
Surviving a global pandemic: the experience of depression, anxiety, and loneliness among individuals with multiple sclerosis	Strober et al.	2022	Ineligible study design	10.1016/J.MSARD.2022.103497
Prevalence of mental health complaints among performing arts students is associated with COVID-19 preventive measures	Stubbe et al.	2021	Ineligible study design	10.3389/fpsyg.2021.676587
Prevalence of depression, anxiety, and perceived stress in postpartum Mexican women during the covid-19 lockdown	Suárez-Rico et al.	2021	Ineligible study design	10.3390/ijerph18094627
In-person contacts and their relationship with alcohol consumption among young adults with hazardous drinking during a pandemic	Suffoletto et al.	2020	Ineligible outcomes	10.1016/J.JADOHEALTH.2020.08.007
Longitudinal associations between internalizing symptoms, social behavior, and social perceptions in the initial months of the COVID-19 pandemic: findings from a transdiagnostic community sample	Swerdlow et al.	2021	Ineligible study design	10.1016/J.JAD.2021.07.093
A longitudinal study of change in substance use from before to during the COVID-19 pandemic in young adults	Sylvestre et al.	2022	Ineligible outcomes	10.1016/j.lana.2021.100168
COVID-19 lockdown leads to changes in alcohol consumption patterns. Results from the Polish national survey	Szajnoga et al.	2020	Ineligible study design	10.1080/10550887.2020.1848247
COVID-19 related depression and anxiety among quarantined respondents	Tang et al.	2021	Ineligible intervention	10.1080/08870446.2020.1782410
Changes in sleep behavior, sleep problems, and psychological distress/health-related quality of life of young Japanese individuals before and during the COVID-19 pandemic	Tanioka et al.	2022	Ineligible study design	10.1080/07420528.2022.2034839
Adapting to uncertainty: a mixed-method study on the effects of the COVID-19 pandemic on expectant and postpartum women and men	Tavares et al.	2021	Ineligible control	10.3389/fpsyg.2021.688340
The impact of COVID-19 lockdown on health behaviors among students of a French university	Tavolacci et al.	2021	Ineligible outcomes	10.3390/ijerph18084346
Job loss predicts worsening depressive symptoms for young adults with autism: a COVID-19 natural experiment	Taylor et al.	2022	Ineligible study design	10.1002/aur.2621
An analysis of mother stress before and during COVID-19 pandemic: the case of China	Tchimtchoua et al.	2020	Ineligible outcomes	10.1080/07399332.2020.1841194
Did the UK COVID-19 lockdown modify the influence of neighbourhood disorder on psychological distress? Evidence from a prospective cohort study	Teo et al.	2021	Ineligible outcomes	10.3389/fpsyt.2021.702807
Sleep quality and physical activity as predictors of mental wellbeing variance in older adults during COVID-19 lockdown: Eclb COVID-19 international online survey	Trabelsi et al.	2021	Ineligible study design	10.3390/ijerph18084329
Substance use, financial stress, employment disruptions, and anxiety among veterans during the COVID-19 pandemic	Tran et al.	2023	Ineligible study design	10.1177/00332941221080413
Are there any cognitive and behavioral changes potentially related to quarantine due to the COVID-19 pandemic in people with mild cognitive impairment and AD dementia? A longitudinal study	Tsatali et al.	2021	Ineligible study design	10.3390/brainsci11091165
U.S. Census Bureau-assessed prevalence of anxiety and depressive symptoms in 2019 and during the 2020 COVID-19 pandemic	Twenge et al.	2020	Ineligible study design	10.1002/da.23077
Mental distress among U.S. adults during the COVID-19 pandemic	Twenge et al.	2020	Ineligible outcomes	10.1002/jclp.23064
Self-isolation due to COVID-19 is linked to small one-year changes in depression, sleepiness, and insomnia: Results from a clinic for sleep disorders in Shiga Prefecture, Japan	Ubara et al.	2020	Ineligible study design	10.3390/ijerph17238971
Health, lifestyle, and psycho-social determinants of poor sleep quality during the early phase of the COVID-19 pandemic: a focus on UK older adults deemed clinically extremely vulnerable	Udeh-Momoh et al.	2021	Ineligible study design	10.3389/fpubh.2021.753964
The risk and protective factors of heightened prenatal anxiety and depression during the COVID-19 lockdown.	Vacaru et al.	2021	Ineligible study design	10.1038/s41598-021-99662-6
Internalizing symptoms and family functioning predict adolescent depressive symptoms during COVID-19: A longitudinal study in a community sample.	Vacaru et al.	2022	Ineligible outcomes	10.1371/journal.pone.0264962
Psychological wellbeing of vulnerable children during the COVID-19 pandemic	Vallejo-Slocker et al.	2020	Ineligible outcomes	10.7334/psicothema2020.218
Hazardous alcohol use among Danish adolescents during the second wave of COVID-19: link between alcohol use and social life	Vallentin-Holbech et al.	2023	Ineligible intervention	10.1177/14550725221149489
Loneliness and mental health during the COVID-19 pandemic: a study among Dutch older adults	van Tilburg et al.	2021	Ineligible study design	10.1093/geronb/gbaa111
Emerging adults’ mental health during the COVID-19 pandemic: a prospective longitudinal study on the importance of social support	van den Berg et al.	2021	Ineligible study design	10.1177/21676968211039979
Self-reported alcohol, tobacco, and cannabis use during COVID-19 lockdown measures: results from a web-based survey	Vanderbruggen et al.	2020	Ineligible study design	10.1159/000510822
Anxiety and depression symptoms, the recovery from symptoms, and loneliness before and after the COVID-19 outbreak among the general population: findings from a Dutch population-based longitudinal study	van der Velden et al.	2021	Ineligible outcomes	10.1371/journal.pone.0245057
Mental health problems among Dutch adolescents of the general population before and 9 months after the COVID-19 outbreak: a longitudinal cohort study	van der Velden et al.	2022	Ineligible outcomes	10.1016/j.psychres.2022.114528
The prevalence, incidence, and risk factors of mental health problems and mental health service use before and 9 months after the COVID-19 outbreak among the general Dutch population. A 3-wave prospective study	van der Velden et al.	2022	Ineligible outcomes	10.1371/journal.pone.0276834
Longitudinal trajectories of study characteristics and mental health before and during the COVID-19 lockdown	van Zyl et al.	2021	Ineligible outcomes	10.3389/fpsyg.2021.633533
Effects of the COVID-19 mitigation measures on alcohol consumption and binge drinking in college students: a longitudinal survey	Vasconcelos et al.	2021	Ineligible study design	10.3390/ijerph18189822
Impact of COVID-19 pandemic on postpartum depression among mothers of extreme and early preterm infants	Vatcheva et al.	2021	No appropriate data	10.1002/ijgo.13859
The mental health impact of the COVID-19 epidemic on college students in India	Verma et al.	2020	Ineligible study design	10.1016/J.AJP.2020.102398
Impact of COVID-19 lockdown on maternal psychological status, the couple’s relationship and mother-child interaction: a prospective study	Viaux-Savelon et al.	2022	Ineligible study design	10.1186/s12884-022-05063-6
Mental health in relation to changes in sleep, exercise, alcohol and diet during the COVID-19 pandemic: examination of four UK cohort studies	Villadsen et al.	2023	Ineligible study design	10.1017/S0033291721004657
Impact of COVID-19-related lockdown on psychosocial, cognitive, and functional well-being in adults with Down syndrome	Villani et al.	2020	Ineligible study design	10.3389/fpsyt.2020.578686
Sleep quality, insomnia symptoms, and depressive symptomatology among Italian university students before and during the COVID-19 lockdown.	Viselli et al.	2021	Ineligible study design	10.3390/ijerph182413346
The impact of the COVID-19 pandemic on stress, mental health and coping behavior in German University students – a longitudinal study before and after the onset of the pandemic	Voltmer et al.	2021	Ineligible study design	10.1186/s12889-021-11295-6
A study of the association between the stringency of COVID-19 government measures and depression in older adults across Europe and Israel	Voss et al.	2021	Ineligible study design	10.3390/ijerph18158017
Attachment anxiety predicts worse mental health outcomes during COVID-19: evidence from two studies	Vowels et al.	2022	Ineligible study design	10.1016/J.PAID.2021.111256
Increased depression during COVID-19 lockdown associated with food insecurity and antiretroviral non-adherence among people living with HIV in Uganda	Wagner et al.	2022	Ineligible control	10.1007/s10461-021-03371-0
Is quarantine related to immediate negative psychological consequences during the 2009 H1N1 epidemic?	Wang et al.	2011	Ineligible intervention	10.1016/j.genhosppsych.2010.11.001
A longitudinal study on the mental health of general population during the COVID-19 epidemic in China	Wang et al.	2020	Ineligible study design	10.1371/journal.pone.0250706
Alcohol consumption in China before and during COVID-19: preliminary results from an online retrospective survey	Wang et al.	2020	Ineligible study design	10.3389/fpsyt.2020.597826
Depressive, anxiety, and insomnia symptoms between population in quarantine and general population during the COVID-19 pandemic: a case-controlled study	Wang et al.	2021	Ineligible intervention	10.1186/s12888-021-03108-2
Bidirectional associations between depressive symptoms and cigarette, e-cigarette, cannabis, and alcohol use: cross-lagged panel analyses among young adults before and during COVID-19	Wang et al.	2022	Ineligible intervention	10.1016/j.addbeh.2022.107422
Drinking to cope during COVID-19 pandemic: the role of external and internal factors in coping motive pathways to alcohol use, solitary drinking, and alcohol problems	Wardell et al.	2020	Ineligible study design	10.1111/acer.14425
Disordered eating and self-harm as risk factors for poorer mental health during the COVID-19 pandemic: a UK-based birth cohort study	Warne et al.	2021	Ineligible study design	10.1186/s40337-021-00510-9
The impact of lockdown stress and loneliness during the COVID-19 pandemic on mental health among university students in Germany	Werner et al.	2021	Ineligible study design	10.1038/s41598-021-02024-5
Parent and child mental health trajectories April 2020 to May 2021: strict lockdown versus no lockdown in Australia	Westrupp et al.	2022	Ineligible intervention	10.1177/00048674211065365
Effect of COVID-19 on BPSD severity and caregiver distress: trend data from national dementia-specific behavior support programs in Australia	Whiting et al.	2021	Ineligible outcomes	10.1002/alz.058454
Tracking the mental health of home-carers during the first COVID-19 national lockdown: evidence from a nationally representative UK survey	Whitley et al.	2023	Ineligible outcomes	10.1017/S0033291721002555
Mental health status of people with multiple sclerosis during the COVID-19 pandemic	Wilski et al.	2022	Ineligible outcomes	10.3390/jcm11030576
Increase in prevalence of current mental disorders in the context of COVID-19: analysis of repeated nationwide cross-sectional surveys	Winkler et al.	2020	Ineligible study design	10.1017/S2045796020000888
Examining family pre-pandemic influences on adolescent psychosocial wellbeing during the COVID-19 pandemic	Wong et al.	2022	Ineligible study design	10.1007/s12144-022-02736-5
Is a pandemic as good as a rest? Comparing athlete burnout and stress before and after the suspension of organised team sport due to Covid-19 restrictions, and investigating the impact of athletes' responses to this period	Woods et al.	2022	Ineligible outcomes	10.1016/J.PSYCHSPORT.2022.102168
Interplay between long‐term vulnerability and new risk: young adolescent and maternal mental health immediately before and during the COVID‐19 pandemic	Wright et al.	2021	Ineligible study design	10.1111/jcv2.12008
Alcohol abuse/dependence symptoms among hospital employees exposed to a SARS outbreak	Wu et al.	2008	Ineligible intervention	10.1093/alcalc/agn073
Increases in anxiety and depression during COVID-19: a large longitudinal study from China	Wu et al.	2021	Ineligible study design	10.3389/fpsyg.2021.706601
Changes of psychotic-like experiences and their association with anxiety/depression among young adolescents before COVID-19 and after the lockdown in China	Wu et al.	2021	Ineligible study design	10.1016/J.SCHRES.2021.08.020
Association of COVID-19 lockdown during the perinatal period with postpartum depression: evidence from rural areas of Western China	Wu et al.	2022	No appropriate data	10.1080/10410236.2022.2036425
Mental well-being, health, and locus of control in Danish adults before and during COVID-19	Würtzen et al.	2021	Ineligible outcomes	10.1017/neu.2021.37
Clinical and functional effects of the COVID-19 pandemic and social distancing on vulnerable veterans with psychosis or recent homelessness	Wynn et al.	2021	Ineligible study design	10.1016/j.jpsychires.2021.03.051
New parents experienced lower parenting self-efficacy during the COVID-19 pandemic lockdown	Xue et al.	2021	Ineligible control	10.3390/children8020079
Gender differences in unpaid care work and psychological distress in the UK COVID-19 lockdown	Xue et al.	2021	Ineligible outcomes	10.1371/journal.pone.0247959
The psychological impact of ‘mild lockdown’ in Japan during the COVID-19 pandemic: a nationwide survey under a declared state of emergency	Yamamoto et al.	2020	Ineligible outcomes	10.3390/ijerph17249382
Impact of the COVID-19 pandemic on mental health among 157,213 Americans	Yarrington et al.	2021	Ineligible study design	10.1016/j.jad.2021.02.056
COVID-19 pandemic and lockdown impacts: a description in a longitudinal study of bipolar disorder	Yocum et al.	2021	Ineligible control	10.1016/j.jad.2021.01.028
Depression, anxiety and PTSD symptoms before and during the COVID-19 pandemic in the UK	Young et al.	2022	Ineligible study design	10.1017/S0033291722002501
COVID-19 lockdown has altered the dynamics between affective symptoms and social isolation among older adults: results from a longitudinal network analysis	Yu et al.	2021	Ineligible study design	10.1038/s41598-021-94301-6
Physical activity, resilience, emotions, moods, and weight control, during the COVID-19 global crisis	Zach et al.	2021	Ineligible outcomes	10.1186/s13584-021-00473-x
Self-assessment of anxiety level and oral hygiene practice in dental students of Cairo University during the COVID-19 pandemic lockdown	Zakaria et al.	2022	Ineligible study design	10.21608/ADJC.2022.99145.1114
Acute impact of a national lockdown during the COVID-19 pandemic on wellbeing outcomes among individuals with chronic pain	Zambelli et al.	2022	Ineligible study design	10.1177/1359105321995962
Psychological impact of COVID-19 quarantine measures in northeastern Italy on mothers in the immediate postpartum period	Zanardo et al.	2020	Ineligible study design	10.1002/ijgo.13249
Immediate and longer-term changes in the mental health and well-being of older adults in England during the COVID-19 pandemic	Zaninotto et al.	2022	Ineligible study design	10.1001/jamapsychiatry.2021.3749
COVID-19 lockdown impact on familial relationships and mental health in a large representative sample of Italian adults	Zeduri et al.	2022	Ineligible study design	10.1007/s00127-022-02273-3
Mental health crisis under COVID-19 pandemic in Hong Kong, China	Zhao et al.	2020	Ineligible study design	10.1016/j.ijid.2020.09.030
The longitudinal association between internet addiction and depressive and anxiety symptoms among Chinese adolescents before and during the COVID-19 pandemic	Zhao et al.	2023	Ineligible study design	10.3389/fpubh.2022.1096660
Pandemic with COVID-19 and families with children with chronic respiratory diseases	Zorcec et al.	2020	Ineligible outcomes	10.2478/prilozi-2020-0038
Psychological health conditions and COVID-19-related stressors among university students: a repeated cross-sectional survey	Zurlo et al.	2021	Ineligible study design	10.3389/fpsyg.2021.741332

Supplementary material 4 

**Table 7:** Characteristics of ongoing studies

Study name	Contact information	Year	Trial registration number
Influence of physical activity during outbreak on psychological states in adults in the COVID-19 pandemic: a study protocol	Marta Camacho-Cardenosa - marta.camacho@imibic.org	2020	NCT04352517
Psychological outcome of COVID-19 lockdown on psychiatric hospital staff and close relatives	Clemence ISAAC - urcve1@gmail.com	2020	NCT04357418
Anxiety and work resilience among tertiary university hospital workers during the COVID-19 outbreak: an online survey	Jean-Yves Lefrant - jean.yves.lefrant@chu-nimes.fr	2020	NCT04358640
The psychological impact of COVID-19 outbreak on COVID-19 survivors and their families	Agnes Yuen-Kwan Lai - agneslai@hku.hk	2020	NCT04365348
The psychological impact of the COVID-19 on students	Agnes Yuen-Kwan Lai - agneslai@hku.hk	2020	NCT04365361
Attention bias modification for reducing health anxiety during the coronavirus pandemic	Yair Bar-Haim - yair1@post.tau.ac.il	2020	NCT04365972
Descriptive study of the psychological impact of confinement measures in the general population	Emilie Olie - e-olie@chu-montpellier.fr	2020	NCT04374643
Death number perception in depression, anxiety, and schizotypal personality in general population	Stéphane Raffard - s-raffard@chu-montpellier.fr	2020	NCT04384419
Consequences of the quarantine relating to the COVID-19 epidemic on the mental health of the patients followed in psychiatry	Arnaud Leroy - arnaud.leroy@chru-lille.fr	2020	NCT04405362
Psychological impact of the health measures generated by the COVID-19 in adolescents	Camille Jung - camille.jung@chicreteil.fr	2020	NCT04406558
Difficulties in emotion-regulation and interpersonal problems during and after the COVID-19 pandemic	Sverre Urnes Johnson - s.u.johnson@psykologi.uio.no	2020	NCT04442282
Stress induced by the COVID-19 pandemic and nonconfinement: study of anxiety factors and potential effects on immunity	Claude Lambert - claude.lambert@chu-st-etienne.fr	2020	NCT04491071
Mental health impact of the COVID-19 pandemic among migrants in Chile	Antonia Errazuriz - anerrazuriz@uc.cl	2020	NCT04497636
CoCo20 protocol: a pilot longitudinal follow-up study about the psychiatric outcomes in a paediatric population and their families during and after the stay- at-home related to coronavirus pandemic	Arnaud Fernandez - fernandez.a@pediatrie- chulenval-nice.fr	2020	NCT04498416
Depression, anxiety and SARS-CoV-2 (COVID-19) phobia in post-stroke patients	Arzu Atici - drsusin@mynet.com	2020	NCT04560413
Physical activity levels of hypertensive and healthy individuals under social isolation during the COVID-19 pandemic	Ebru Calik Kutukcu - ebrucalk85@hotmail.com	2020	NCT04583345
Finding wellness in the pandemic [improving health and wellness during COVID-19]	Not applicable	2020	NCT04615741
Anxiety, depression and eating attitudes of diabetes mellitus patients during COVID-19 lockdown in Greece	Emmanouil S Benioudakis - manolis2668@hotmail.gr	2020	NCT04700254
Emotional, social, cognitive and behavioral sequalae of the COVID-19 pandemic	Morgan Andrews - deborah.roberts@nih.gov	2021	NCT04823988
Estimating the prevalence of postpartum anxiety and depression in the context of the coronavirus disease (COVID-19) pandemic	Elie AZRIA - eazria@ghpsj.fr	2021	NCT04852757
#Stayhealthy - monitoring and maintenance of mental health under conditions of social isolation during the corona crisis (stayhealthy)	Ann-Christine Ehlis - ann-christine.ehlis@med.uni-tuebingen.de	2020	NCT04871386
Depression and anxiety in long term coronavirus disease COVID-19 (DALT-COV)	Bumi Herman - bumiherman@med.unhas.ac.id	2021	NCT04893668
Psycho-traumatic consequences of the COVID-19 health crisis among professionals in emergency services (COVER PRO LT)	Marion Douplat - marion.douplat@chu-lyon.fr	2021	NCT05033223
Stress among final year BAMS students in relation with covid lockdown through CSSQ-a cross sectional survey	Preethi Mohan - drpreeti94@gmail.com	2021	NCT05241080

Supplementary material 5 

**Table 8:** Detailed characteristics of included studies

Acharya et al. (2022) [41]	
Study characteristics	
Methods	A retrospective study analyzing the suicide trends in Nepal. Key study design feature: time differences. Study date: the control group was June 2019, and the intervention group was June 2021. Country income classification: middle- or low-income countries in 2020. Setting: whole Nepal region. All pre-specified confounders were not adjusted for. An adjustment model accounting for cluster factors was not used, as individuals were not a clustering factor.
Participants	General population in Nepal. Age and gender: not available.
Interventions	Type of intervention: lockdown. Periods of intervention: from March 24, 2020 (the end of lockdown was unclear). Pre-specified co-interventions: not available
Outcomes	(1) Suicide. Every case of unnatural death in Nepal is investigated by the police department as required by domestic law. The police department determines the nature of death as a suicide based on the medical and autopsy reports. Follow-up period: 1 year and 3 months. The number and the proportion of suicides in the intervention period (June 2019) were 732 and 2.43 (per 100,000), and the number and the proportion in the control period were 604 and 2.06 (per 100,000). The incidence rate ratio between the two periods was 1.33 (95% CI 1.2-1.48).
Notes	Country: Nepal. Funding source: no specific funding for this work. Contact author: Binod Acharya - ba525@drexel.edu.
Albrecht et al. (2022) [42]	
Study characteristics	
Methods	Study design: three cross-sectional online surveys. Key study design feature: time differences. Study date: the intervention group was spring 2021, and the control group was spring 2017. Country income classification: high-income countries in 2020. Setting: 21 high schools in the Canton of Zurich, Switzerland. All pre-specified confounders were not adjusted for, and an adjustment model accounting for cluster factors (school) was used.
Participants	Type of participants: 21 high school students (adolescents). Total number of participants: 12,238. Age (mean ± SD): intervention group, 16 ± 2.22 years; control group, 16 ± 1.48 years. Gender: intervention group, 67.5% females/32.5% males; control group, 65.1% females/34.5% males.
Interventions	Type of intervention: lockdown. Period of interventions: not available. Pre-specified co-interventions: not available.
Outcomes	(1) Substance use: tobacco. This was measured by an online survey, and the follow-up period was 12 months. In the intervention group, the number of follow-up participants was 108, and the mean (SD) number of cigarettes per day was 4.41 (4.4). In the control group, the number of follow-up participants was 205, and the mean (SD) number of cigarettes per day was 5.84 (5). The coefficient estimated by mixed models between the two groups was -1.28 (SE 0.57; p = 0.29). (2) Substance use: alcohol. This was measured by an online survey, and the follow-up period was 12 months. In the intervention group, the number of follow-up participants was 1436, and the mean (SD) total score about alcohol consumption was 2.35 (2.29). In the control group, the number of follow-up participants was 2774, and the mean (SD) number of total score about alcohol consumption was 2.38 (2.08). The coefficient estimated by mixed models between two groups was -0.13 (SE, 0.07; p = 0.9).
Notes	Country: Switzerland. Funding source: no external funding. Contact author: Reto Huber - reto.huber@kispi.uzh.ch.
Arad et al. (2021) [43]	
Study characteristics	
Methods	Key study design feature: time differences. Study date: the control group was spring semester in 2016-2019, and the intervention group was spring semester in 2019-2020. Country income classification: high-income countries in 2020. Setting: Tel Aviv University. All pre-specified confounders were not adjusted for. An adjustment model accounting for cluster factors was not used, as individuals were not a clustering factor.
Participants	Type of participants: undergraduate freshmen at Tel Aviv University (adults). Total number: 99 (55 intervention group and 44 control group). Age (mean ± SD): intervention group, 22.6 ± 2.36 years; control group, 21.57 ± 1.9 years. Gender: intervention group, 49 females/6 males; control group, 35 females/9 males.
Interventions	Type of intervention: COVID-19 lockdown. Period of interventions: more than 1 month. Pre-specified co-interventions: not available.
Outcomes	(1) Anxiety symptom severity. This was measured using the Liebowitz Social Anxiety Scale, 1 month after the lockdown. The mean (SD) score in the intervention group was 70.62 (18.65), and the score in the control group was 53.51 (15.83). The effect of the intervention calculated by analysis of covariance (ANCOVA) was estimated to be F(1, 86) 15.71 and η^2^ = 0.15 (p < 0.001).
Notes	Country: Israel. Funding source: Israel Science Foundation (Grant# 1811/17). Contact author: Dana Shamai-Leshem - dash1903@gmail.com.
Barbosa et al. (2021) [44]	
Study characteristics	
Methods	Study design: a cross-sectional study. Key study design feature: time differences. Study date: the control group was February 2020, and the intervention group was April 2020. Country income classification: high-income countries in 2020. Setting: online survey of alcohol drinking patterns was conducted using the Ipsos KnowledgePanel. One pre-specified confounder (economic status) was not adjusted for, and an adjustment model accounting for cluster factors (individuals) was used.
Participants	Type of participants: US general population (adults). Total number of participants: 556. Age: intervention group, 21-34 (24.7%), 35-49 (25.3%), 50-64 (29.7%), and 65 and older (20.4%); control group, not available. Gender: intervention group, 52.3% females/47.7% males; control group, 52.3% females/47.7% males.
Interventions	Type of intervention: stay-at-home orders. Period of interventions: not available. Pre-specified co-interventions: not available.
Outcomes	(1) Substance use: alcohol. Follow-up period was 1 month after the intervention. In the intervention group, the number of follow-up participants was 556, and the mean (SD) number of drinks per drinking day was 2.72 (2.1). In the control group, the number of follow-up participants was 556, and the mean (SD) number of drinks per drinking day was 2.47 (1.82). The coefficient estimated by a linear regression model between two groups was 0.08 (SE, 0.33).
Notes	Country: United States. Funding source: supported by the authors’ employing organization: RTI International. Contact author: Carolina Barbosa - cbarbosa@rti.org.
Bartlett et al. (2021) [45]	
Study characteristics	
Methods	A longitudinal study before and during the first COVID-19 lockdown period. Key study design feature: time differences. Study date: the control group was October 2019, and the intervention group was from April 23 to May 5, 2020. Country income classification: high-income countries in 2020. Setting: Tasmania. One pre-specified confounder (relationship status) was adjusted for, and an adjustment model accounting for cluster factors (individuals) was used.
Participants	Type of participants: anyone living in Tasmania who was 50+ years old. Total number of participants: 1671. Age (mean ± SD): control group, 63.4 ± 7.17 years. Gender: 1218 females/452 males.
Interventions	Type of intervention: lockdown restrictions. Period of interventions: March 30 to May 11, 2020. Pre-specified co-interventions: not available.
Outcomes	(1) Depressive symptom severity. This was measured using the Hospital Anxiety and Depression Scale (HADS) - Depression, and the follow-up period was 1-2 months. In the intervention group, the number of follow-up participants was 1671, and the mean (SD) score was 2.05 (2.19). In the control group, the number of follow-up participants was 1671, and the mean (SD) score was 2.07 (2.09). The standardized mean difference between two groups was -0.01 (p = 0.593). (2) Anxiety symptom severity. This was measured using HADS - Anxiety, and the follow-up period was 1-2 months. In the intervention group, the number of follow-up participants was 1671, and the mean (SD) score was 4.88 (3.34). In the control group, the number of follow-up participants was 1671, and the mean (SD) score was 5.56 (3.55). The standardized mean difference between the two groups was -0.2 (p < 0.001). (3) Substance use: alcohol. Number of standard drinks per drinking occasion x drinking frequency per week was measured, and the follow-up period was 1-2 months. In the intervention group, the number of follow-up participants was 1671, and the mean (SD) score was 3.34 (4.65). In the control group, the number of follow-up participants was 1671, and the mean (SD) score was 3.02 (4.11). The standardized mean difference between the two groups was -0.07 (p < 0.001).
Notes	Country: Australia. Funding source: Medical Research Futures Fund; University of Tasmania; St Lukes Health; Masonic Centenary Medical Research Foundation. Contact author: Duncan Sinclair - duncan.sinclair@utas.edu.au.
Bennett et al. (2022) [46]	
Study characteristics	
Methods	Key study design feature: time differences. Study date: the control lockdown group was May 6-27, 2019, and the intervention group was June 22 to July 12, 2020. Country income classification: high-income countries in 2020. Setting: a large UK university. All pre-specified confounders were not adjusted for. An adjustment model accounting for cluster factors was not used, as individuals were not a clustering factor.
Participants	Type of participants: all registered students (undergraduate, postgraduate-taught, and postgraduate research) within a large UK university (adults). Total number: 6330 (3693 intervention group and 2637 control group). Age (mean ± SD): intervention group, <25 (2900), ≥25 (787), and missing (6); control group, 22.6 ± 6.44 years. Gender: intervention group, 2411 females/1209 males; control group, 1829 females/720 males.
Interventions	Type of intervention: national lockdown. Period of interventions: March 23, 2020, to summer 2020. Pre-specified co-interventions: not available.
Outcomes	(1) Depressive symptom severity. This was measured using the Patient Health Questionnaire (PHQ-9), 3-4 months after the lockdown. The mean (SD) score in the intervention group was 8.6 (5.9), and the score in the control group was 9.88 (6.7). The effect of the intervention calculated by a linear regression model was estimated to be -1.28 (95% CI -1.59 to -0.97, p < 0.001). (2) Anxiety symptom severity. This was measured using the Generalized Anxiety Disorder Scale (GAD-7), 3-4 months after the lockdown. The mean (SD) score in the intervention group was 7.09 (5.48), and the score in the control group was 8.04 (5.85). The effect of the intervention calculated by a linear regression model was estimated to be -0.95 (95%CI -1.23 to -0.67, p < 0.001).
Notes	Country: United Kingdom. Funding source: the Elizabeth Blackwell Institute, University of Bristol. Contact author: Myles-Jay Linton - mj.linton@bristol.ac.uk.
Berthelot et al. (2020) [47]	
Study characteristics	
Methods	A longitudinal study in prenatal clinics before the COVID-19 pandemic. Key study design feature: time differences. Study date: the no lockdown group was April 2018 to March 1, 2020, and the lockdown group was April 2-13, 2020. Country income classification: high-income countries in 2020. Setting: the Province of Quebec, Canada. One pre-specified confounder (economic status) was adjusted for, and an adjustment model accounting for cluster factors was not used.
Participants	Type of participants: pregnant women (adults). Total number: 2078 (1754 intervention group and 324 control group). Age (mean ± SD): intervention group, 29.4 ± 4.04 years; control group, 29.1 ± 4.65 years.
Interventions	Type of intervention: public health emergency. Period of interventions: March 24 to May 4, 2020. Pre-specified co-interventions: not available.
Outcomes	(1) Post-traumatic stress disorder symptom severity. This was measured using the PTSD checklist for DSM-5 (PCL-5), 2 weeks to 1 month after the lockdown. The mean (SE) score in the intervention group was 0.06 (0.03), and the score (SE) in the control group was -0.12 (0.04). The effect size of the intervention calculated by a multivariate analysis of covariance was estimated to be 0.19 (p = 0.001).
Notes	Country: Canada. Funding source: not available. Contact author: Nicolas Berthelot - nicolas.berthelot@uqtr.ca.
Boekhorst et al. (2021) [48]	
Study characteristics	
Methods	A longitudinal prospective cohort study. Key study design feature: time differences. Study date: the control group was January 7, 2019, to March 1, 2020, and the intervention group was March 1, 2020, to May 14, 2020. Country income classification: high-income countries in 2020. Settings: community midwife practices and hospitals in Brabant, Netherlands. Two pre-specified confounders (occupation and relationship status) were not adjusted for, and an adjustment model accounting for cluster factors (individuals) was used.
Participants	Type of participants: Dutch pregnant women (18+ years) who had their first antenatal visit before 14 weeks of gestation (adults). Total number: 669 (268 intervention group and 401 control group). Age (mean ± SD): intervention group, 30.75 ± 3.64 years; control group, 30.88 ± 3.67 years.
Interventions	Type of intervention: strict nationwide lockdown. Period of interventions: March 1 to May 14, 2020. Pre-specified co-interventions: not available.
Outcomes	(1) Depressive symptom severity. This was measured using the 10-item Edinburgh (Postnatal) Depression Scale (E(P) DS), 0-1 month after the lockdown. In the first trimester, the mean (SD) score in the intervention group was 4 (3.7), and the score in the control group was 4 (4.4). In the second trimester, the mean (SD) score in the intervention group was 4 (4.4), and the score in the control group was 4 (3.7). In the third trimester, the mean (SD) score in the intervention group was 5.5 (4.4), and the score in the control group was 4 (5.2). The effect of the intervention calculated by a mixed model was estimated to be -0.03 (SE 0.31, p = 0.925).
Notes	Country: Netherlands. Funding source: Tilburg University. Contact author: Myrthe G. B. M. Boekhorst - m.g.b.m.boekhorst@uvt.nl.
Bouter et al. (2023) [49]	
Study characteristics	
Methods	A longitudinal study within the iBerry (Investigating Behavioral and Emotional Risk in Rotterdam Youth) Study. Key study design feature: time differences. Study date: the control group was 2014–2015 and 2015–2016 academic year, and the intervention group was April 24 to June 4, 2020. Country income classification: high-income countries in 2020. Settings: the greater Rotterdam area of the Netherlands. All pre-specified confounders were not adjusted for, and an adjustment model accounting for cluster factors (individuals) was used.
Participants	Type of participants: adolescents designed to investigate the transition from subclinical symptoms to a psychiatric disorder. Total number of participants: 445. Age (mean ± SD): intervention group, 17.7 ± 0.62 years; control group, not available. Gender: both groups comprised 226 females/219 males.
Interventions	Type of intervention: lockdown. Period of interventions: six weeks. Pre-specified co-interventions: not available.
Outcomes	(1) Depressive symptom severity. This was measured by subscales of the Youth Self-Report (YSR) from the Achenbach System of Empirically Based Assessment (ASEBA) – Depression, 1-2 months after the intervention. In the intervention group, the mean (SD) score was 6.34 (4.88). In the control group, the mean (SD) score was 5.06 (3.99). The coefficient estimated by multilevel random intercept regression models between two groups was 0.93 (95% CI 0.43, 1.42). (2) Anxiety symptom severity. This was measured by subscales of the YSR from the ASEBA – Anxiety, 1-2 months after the intervention. In the intervention group, the mean (SD) score was 3.92 (3.61). In the control group, the mean (SD) score was 4.05 (3.13). The coefficient estimated by multilevel random intercept regression models between two groups was -0.58 (95% CI -0.94, -0.21).
Notes	Country: Netherlands. Funding source: The iBerry Study is funded by the Erasmus University Medical Center and the following institutes of mental health care (GGz): Parnassia Psychiatric Institute Antes, GGz Breburg, GGz Delfland, GGz Westelijk Noord-Brabant, and Yulius. All funding organizations participate in the Epidemiological and Social Psychiatric Research Institute (ESPRi), a consortium of academic and non-academic research groups. Contact author: N. H. Grootendorst-van Mil - n.grootendorst@erasmusmc.nl.
Burdzovic Andreas and Brunborg (2022) [50]	
Study characteristics	
Methods	A longitudinal study. Key study design feature: time differences. Study date: the control group was fall 2018 and 2019, and the intervention group was fall 2020. Country income classification: high-income countries in 2020. Setting: 33 middle schools throughout Norway. Two pre-specified confounders (economic status and relationship status) were adjusted for, and an adjustment model accounting for cluster factors (schools) was used.
Participants	Type of participants: students in grades 8-10 (adolescents). Total number: 2572 (951 intervention group and 1621 control group). Age: both groups were 16-17 years (grade 11). Gender: intervention group, 553 females/362 males; control group, 952 females/669 males.
Interventions	Type of intervention: strict nationwide lockdown. Period of interventions: March 12 to May-June 2020. Pre-specified co-interventions: not available.
Outcomes	(1) Substance use: alcohol. This was measured by an online survey, six months after the lockdown. In the intervention group, the mean (SD) number of alcoholic drinks consumed on a drinking day was 2.76 (6.05). In the control group, the mean (SD) number was 2.42 (4.48). The incidence risk ratios estimated by a linear regression model between two groups was 1.13 (95% CI 1.02, 1.25; p = 0.02).
Notes	Country: Norway. Funding source: no external funding. Contact author: Jasmina Burdzovic Andreas - jabu@fhi.no.
Cellini et al. (2021) [51]	
Study characteristics	
Methods	Study design: a cross-sectional online survey. Key study design feature: time differences. Study date: the control group was until March 10, 2020, for Italy and March 19, 2020, for Belgium, and the intervention group was April 1 to May 19, 2020, in both countries. Country income classification: high-income countries in 2020. Setting: the whole nation via social media and university websites. One pre-specified confounder (occupation) was adjusted for, and an adjustment model accounting for cluster factors (individuals) was used.
Participants	Type of participants: general population in Italy and Belgium (adult). Total number of participants: 1622 in Italy and 650 in Belgium. Age (mean ± SD): intervention group, 34.1 ± 13.6 years (Italy) and 43 ± 16.8 years (sample from Belgium). Gender: 1171 females/451 males (Italy) and 509 females/141 males (Belgium).
Interventions	Type of intervention: lockdown. Period of interventions: In Italy, March 10 to May 3, 2020, and in Belgium, March 18 to May 4, 2020. Pre-specified co-interventions: not available.
Outcomes	(1) Insomnia symptom severity. This was measured using the Pittsburgh Sleep Quality Index, 1-2 months after the lockdown. In Italy sample, the mean (SD) score in the intervention group was 6.56 (3.63), the score in the control group was 5.19 (2.69), and the effect size of the intervention calculated by a linear mixed model was estimated to be F(1, 1612) = 101.51 (p < 0.001). In the sample from Belgium, the mean (SD) score in the intervention group was 6.48 (3.61), the score in the control group was 5.9 (3.06), and the effect size of the intervention calculated by a linear mixed model was estimated to be F(1642) = 7.1 (p = 0.008).
Notes	Country: Italy and Belgium. Funding source: a European Research Council starting grant (CS; ERC-StG 757763). Contact author: Nicola Cellini - nicola.cellini@unipd.it.
Cellini et al. (2021) [52]	
Study characteristics	
Methods	A cross-sectional online survey. Key study design feature: time differences. Study date: the control group was February 24-29, 2020, and the intervention group was April 1-9, 2020. Country income classification: high-income countries in 2020. Settings: Italian national territory. One pre-specified confounder (occupation) was adjusted for, and an adjustment model accounting for cluster factors (individuals) was used.
Participants	Type of participants: mothers who were at least 18 years old, were living in the Italian national territory, and had at least one child between 6 and 10 years old. Total number: 299. Age (mean ± SD): intervention group, 40.2 ± 4.79 years; control group, not available. Gender: 299 females/0 males.
Interventions	Type of intervention: a national lockdown. Period of interventions: not available. Pre-specified co-interventions: not available.
Outcomes	(1) Insomnia symptom severity. This was measured using the Pittsburgh Sleep Quality Index, 3-4 weeks after the lockdown. The mean (SD) score in the intervention group was 5.66 (2.77), and the score in the control group was 3.73 (2.77). The effect size of the intervention calculated by a linear mixed model was estimated to be F(1, 291) = 4.93 (p = 0.027).
Notes	Country: Italy. Funding source: no external funding. Contact author: Nicola Cellini - nicola.cellini@unipd.it.
Cody et al. (2021) [53]	
Study characteristics	
Methods	A cross-sectional analysis within a randomized controlled trial. Key study design feature: time differences. Study date: the control group was January 2019 to February 2020, and the intervention group was May to December 2020. Country income classification: high-income countries in 2020. Settings: four centers in three Swiss cantons (Basel, Solothurn, and Bern). All pre-specified confounders were not adjusted for. An adjustment model accounting for cluster factors was not used, as individuals were not a clustering factor.
Participants	Type of participants: women and men between 18 and 65 years, ICD-10-diagnosed depressive episode (single episode or recurrent), Beck's Depression Inventory score (BDI) of at least 17 representing clinical depression, physical inactivity as defined by < 150 min of moderate-to-vigorous physical activity per week prior to in-patient treatment, and adequate German language skills. Total number: 165 (46 intervention group and 119 control group). Age (mean ± SD): intervention group, 41.74 ± 13.09 years; control group, 41.94 ± 12.29 years. Gender: intervention group, 21 females/25 males; control group, 63 females/56 males.
Interventions	Type of intervention: COVID-19 lockdown. Period of interventions: from March until the end of April 2020. Pre-specified co-interventions: not available.
Outcomes	(1) Insomnia symptom severity. This was measured using the Insomnia Severity Index (ISI), 2-9 months after the lockdown. The mean (SD) score in the intervention group was 11.82 (6.31), and the score in the control group was 12.23 (5.17). The effect size of the intervention calculated by analysis of covariance (ANCOVA) was estimated to be F = 0.26 (p = 0.61).
Notes	Country: Switzerland. Funding source: the Swiss National Science Foundation (Grant No. 321003B-179353). Contact author: Robyn Cody - robyn.cody@unibas.ch.
Cohen et al. (2021) [54]	
Study characteristics	
Methods	A cohort study using a sample of patients with hand and wrist conditions. Key study design feature: time differences. Study date: the control group was 2018-2019, and the intervention group was March 23 to May 4, 2020. Country income classification: high-income countries in 2020. Setting: Xpert Clinic and Handtherapie Nederland, comprising 28 clinics for hand surgery and therapy in the Netherlands. One pre-specified confounder (occupation) was adjusted for, and an adjustment model accounting for cluster factors (individuals) was used.
Participants	Type of participants: patients with hand and wrist conditions (adults). Total number: 535 (313 intervention group and 222 control group). Age (mean ± SD): intervention group, 57 ± 13 years; control group, 59 ± 12 years. Gender: intervention group, 209 females/104 males; control group, 137 females/85 males.
Interventions	Type of intervention: intelligent lockdown. Period of interventions: not available. Pre-specified co-interventions: not available.
Outcomes	(1) Depressive symptom severity. This was measured using the Patient Health Questionnaire (PHQ-4) – Depression, 2 weeks to 1 month after the lockdown. The mean (SD) score in the intervention group was 0 (0), and the score in the control group was 0 (0). The standardized mean difference calculated by a multivariable linear mixed model was 0.12 (p = 0.11). (2) Anxiety symptom severity. This was measured using the Patient Health Questionnaire (PHQ-4) – Anxiety, 2 weeks to 1 month after the lockdown. The mean (SD) score in the intervention group was 0 (0), and the score in the control group was 0 (0.74). The standardized mean difference calculated by a multivariable linear mixed model was 0.09 (p = 0.28).
Notes	Country: Netherlands. Funding source: ZonMW (The Hague, Netherlands) and CZ (Tilburg, Netherlands). Contact author: Abigael Cohen - a.cohen.1@erasmusmc.nl.
Cousijn et al. (2021) [55]	
Study characteristics	
Methods	A cross-sectional online survey. Key study design feature: time differences. Study date: the control group was January 2019 to February 2020, and the intervention group was April to May 2020. Country income classification: high-income countries in 2020. Setting: Netherlands. All pre-specified confounders were not adjusted for, and an adjustment model accounting for cluster factors (individuals) was used.
Participants	Type of participants: daily or near-daily cannabis users who do not regularly use other illicit substances (adult). Total number of participants: 120. Age (range): intervention group, 18-46 years; control group, 18-31 years. Gender: not available.
Interventions	Type of intervention: Dutch lockdown. Periods of intervention: not available. Pre-specified co-interventions: not available.
Outcomes	(1) Substance use: cannabis. This was measured by an online survey, and the follow-up period was mean 59 (SD, 8.6) days after the lockdown. In the intervention group, the number of follow-up participants was 109, and the mean (SD) days per month was 22 (10.5). In the control group, the number of follow-up participants was 109, and the mean (SD) days per month was 20.8 (10.7). The coefficient estimated by a linear mixed model between two groups was 1.96 (95% CI, 1.26-3.66; p = 0.024).
Notes	Country: Netherlands. Funding source: grant 1R01 DA042490-01A1 from the National Institute on Drug Abuse. Contact author: Janna Cousijn - j.cousijn@gmail.com.
Dunn et al. (2021) [56]	
Study characteristics	
Methods	Key study design feature: time differences. Study date: the control group was between the pre- and during-COVID conditions, ranging between 2 and 20 months (mean = 10.2 months; SD = 4.18), and the intervention group was April 23 to May 8, 2020. Country income classification: high-income countries in 2020. Setting: in the Midwestern United States. All pre-specified confounders were not adjusted for, and an adjustment model accounting for cluster factors was (individuals) used.
Participants	Type of participants: adult participants performing cochlear implant. Total number: 48. Age (mean ± SD): intervention group, 60 ± 12.7 years; and control group, not available. Gender: 29 females/19 males.
Interventions	Type of intervention: a State of Public Health Disaster Emergency. Period of interventions: March 17 to May 1, 2020. Pre-specified co-interventions: not available.
Outcomes	(1) Depressive symptom severity. This was measured using the Beck’s Depression Inventory-II, 1-2 months after the lockdown. The mean (SD) score in the intervention group was 6.76 (6.98), and the score in the control group was 6.08 (6.98). The effect size of the intervention calculated by a mixed effect model was estimated to be -1.13 (95% CI -3.64, 1.38; p = 0.376). (2) Anxiety symptom severity. This was measured using the Beck’s Anxiety Inventory, 1-2 months after the lockdown. The mean (SD) score in the intervention group was 6.76 (9.46), and the score in the control group was 5.4 (5.24). The effect size of the intervention calculated by a mixed effect model was estimated to be -1.21 (95% CI -3.88, 1.48; p = 0.379).
Notes	Country: United States. Funding source: research grant 2P50DC000242 from the National Institutes on Deafness and Other Communication Disorders, National Institutes of Health; the Lions Clubs International Foundation; the Iowa Lions Foundation. Contact author: Camille Dunn - Camille-dunn@uiowa.edu.
Gonzalez-Martinez et al. (2021) [57]	
Study characteristics	
Methods	A longitudinal study. Key study design feature: time differences. Study date: the control group was December 2019 to March 3, 2020, and the intervention group was September 9, 2020, to January 2021. Country income classification: high-income countries in 2020. Setting: the Refractory Epilepsy Unit from a tertiary hospital in Spain. All pre-specified confounders were not adjusted for, and an adjustment model accounting for cluster factors (individuals) was used.
Participants	Type of participants: patients with epilepsy (adult). Total number of participants: 158 (73 intervention group and 85 control group). Age (mean ± SD): intervention group, 42.1 ± 15.6 years; control group, 44.3 ± 17.4 years. Gender: intervention group, 34 females/39 males; control group, 41 females/44 males.
Interventions	Type of intervention: lockdown. Period of interventions: not available. Pre-specified co-interventions: not available.
Outcomes	(1) Anxiety symptom severity. This was measured using the Spanish version of the 7-item Generalized Anxiety Disorder, 6-10 months after the lockdown. The mean (SD) score in the intervention group was 7.9 (5.7), and the score in the control group was 7.6 (5.4). The coefficient of the intervention calculated by the generalized linear mixed model was estimated to be -1.65 (95% CI -3.02, -0.43; p = 0.023). (2) Insomnia symptom severity. This was measured using the Epworth Sleepiness Scale, 6-10 months after the lockdown. The mean (SD) score in the intervention group was 7.7 (4.6), and the score in the control group was 5.5 (3.9). The coefficient of the intervention calculated by the generalized linear mixed model was estimated to be 2.39 (95% CI 1.05, 3.74; p = 0.001).
Notes	Country: Spain. Funding source: no specific funding to report. Contact author: Alicia Gonzalez-Martinez - alicia.gonzalez.martinez@live.com.
Hausman et al. (2022) [58]	
Study characteristics	
Methods	Key study design feature: time differences. Study date: the control group was August 2017 to March 2020, and the intervention group was July 2020 to March 2021. Country income classification: high-income countries in 2020. Setting: a multisite clinical trial: the Augmenting Cognitive Training in Older Adults study (ACT, R01AG054077). All pre-specified confounders were not adjusted for, and an adjustment model accounting for cluster factors (individuals) was used.
Participants	Type of participants: participants aged 65-89 who were randomized to receive an intervention that included a combination of transcranial direct current stimulation (active vs. sham) and cognitive training or educational training (adult). Total number: 189. Age (mean ± SD): 71.4 ± 4.8 years. Gender: 123 females/66 males.
Interventions	Type of intervention: stay-at-home orders. Period of interventions: not available. Pre-specified co-interventions: not available.
Outcomes	(1) Depressive symptom severity. This was measured using the Beck’s Depression Inventory-II (BDI-II), 4-12 months after the lockdown. The mean (SD) score in the intervention group was 5.7 (5.9), and the score in the control group was 3.4 (3.9). The effect size of the intervention calculated by a mixed-effects model was estimated to be 2.32 (p < 0.001). (2) Anxiety symptom severity. This was measured using the State Trait Anxiety Inventory (STAI) - State and Trait, 4-12 months after the lockdown. The mean (SD) score of STAI - State in the intervention group was 28 (9.2), and the score in the control group was 28.4 (8.3). The effect size of the intervention calculated by a mixed-effects model was estimated to be -0.25 (p = 0.76). The mean (SD) score of STAI - Trait in the intervention group was 27.9 (9), and the score in the control group was 28.6 (7.4). The effect size of the intervention calculated by a mixed-effects model was estimated to be -0.59 (p = 0.32). (3) Insomnia symptom severity. This was measured using the Pittsburgh Sleep Quality Index (PSQI), 4-12 months after the lockdown. The mean (SD) score in the intervention group was 6 (3.5), and the score in the control group was 5.5 (3.2). The effect size of the intervention calculated by a mixed-effects model was estimated to be 0.45 (p = 0.01).
Notes	Country: United States. Funding source: the National Institute on Aging (NIA R01AG054077, NIA P30AG019610, T32AG020499), the State of Arizona and Arizona Department of Health Services (ADHS), the University of Florida Center for Cognitive Aging and Memory Clinical Translational Research, and the McKnight Brain Research Foundation. Contact author: Adam J. Woods - ajwoods@phhp.ufl.edu.
Kekäläinen et al. (2021) [59]	
Study characteristics	
Methods	A longitudinal study using data from Estrogen, microRNAs, and the risk of metabolic dysfunction (EsmiRs) study. Key study design feature: time differences. Study date: the control group was November 12, 2018 to March 16, 2020, and the intervention group was May 15 to June 16, 2020. Country income classification: high-income countries in 2020. Setting: the city of Jyväskylä, Finland, and neighboring municipalities. Two pre-specified confounders (occupation and relationship) were not adjusted for, and an adjustment model accounting for cluster factors (individuals) was used.
Participants	Type of participants: 47- to 55-year-old Finnish women. Total number: 358. Age (mean ± SD): 54.3 ± 2 years.
Interventions	Type of intervention: lockdown. Period of interventions: March 17 to June 16, 2020. Pre-specified co-interventions: not available.
Outcomes	(1) Depressive symptom severity. This was measured using the Centre for Epidemiological Studies - Depression Scale (CES-D), 2-3 months after the lockdown. The mean (SD) score in the intervention group was 0.53 (0.38), and the score in the control group was 0.45 (0.36). The effect size (Wald test) of the intervention calculated by a general equation estimation model was estimated to be 9.26 (p < 0.001). (2) Substance use: alcohol. The follow-up period was 2-3 months. In the intervention group, the mean (SD) weekly amount of alcohol consumption was 2.98 (3.6). In the control group, the mean (SD) weekly amount of alcohol consumption was 3.22 (3.51). The effect size (Wald test) of the intervention calculated by a general equation estimation model was estimated to be 1.87 (p = 0.171).
Notes	Country: Finland. Funding source: the Ministry of Education and Culture of Finland (OKM/49/626/2017, OKM/72/626/2018, OKM/92/626/2019), the Academy of Finland (No. 275323 and EKL: 309504), and the Ministry of Education and Culture of Finland (OKM/49/626/2017, OKM/72/626/2018). Contact author: Tiia Kekäläinen - tiia.m.kekalainen@jyu.fi.
Koenders et al. (2021) [60]	
Study characteristics	
Methods	An ecological add-on study to the Bipolar Netherlands Cohort (BINCO). Key study design feature: time differences. Study date: the control group was 2018-2019, and the intervention group was September to October 2020. Country income classification: high-income countries in 2020. Setting: mental health outpatient clinics in the Netherlands. All pre-specified confounders were not adjusted for, and an adjustment model accounting for cluster (individuals) factors was used.
Participants	Type of participants: recently diagnosed (<1 year) bipolar I and II patients. Total number of participants: 36. Age (mean ± SD): 36.7 ± 12.6 years. Gender: 20 females/16 males.
Interventions	Type of intervention: lockdown. Period of interventions: March 24 to May 11, 2020. Pre-specified co-interventions: not available.
Outcomes	(1) Depressive symptom severity. This was measured using the 16-item Quick Inventory of Depressive Symptomatology (QIDS- SR), 6-7 months after the lockdown. The mean (SD) score in the intervention group was 8.46 (5.29), and the score in the control group was 11.2 (6.4). The effect size of the intervention calculated by a multilevel linear mixed model was estimated to be χ^2^ = 7.45 (p = 0.28).
Notes	Country: Netherlands. Funding source: Cella Durksz fund, Grant/Award Number: CWB 5267; LUF/Gratama fund, and Grant/Award Number: 2016-10 CWB 6515. Contact author: Manja Koenders - m.a.koenders@fsw.leidenuniv.nl.
Koenig et al. (2023) [61]	
Study characteristics	
Methods	A longitudinal study using data from the ongoing ProHEAD project. Key study design feature: time differences. Study date: the control group was November 2018 to March 15, 2020, and the intervention group was March 16 to August 2020. Country income classification: high-income countries in 2020. Setting: a multi-center consortium situated at five study sites across Germany and led by the managing site at the University Hospital of Heidelberg. One pre-specified confounder (occupation) was adjusted for. An adjustment model accounting for cluster factors was not used, as individuals were not a clustering factor.
Participants	Type of participants: mental health problems in a sample of children and adolescents aged ≥ 12 years (adolescents). Total number of participants: 324. Age (mean ± SD): 14.93 ± 1.88 years. Gender: intervention group, 225 females/99 males; control group, 224 females/100 males.
Interventions	Type of intervention: lockdown. Period of interventions: not available. Pre-specified co-interventions: not available.
Outcomes	(1) Depressive symptom severity. This was measured using the 9-item version of the Patient Health Questionnaire (PHQ-9) modified for Adolescents, immediately and two months after the lockdown. The number of participants in the intervention group was 219, and the mean (SD) score was 7.39 (4.94). The number of participants in the control group was 243, and the mean (SD) score was 7.95 (5.55). The coefficient of the intervention calculated by a linear regression was estimated to be -0.13 (p = 0.062).
Notes	Country: Germany. Funding source: the German Federal Ministry of Education and Research (BMBF) Grant (01GL1744B). Contact author: Michael Kaess - kaess@med.uni-heidelberg.de.
Leatherdale et al. (2023) [62]	
Study characteristics	
Methods	A longitudinal study using data from COMPASS study, Key study design feature: time differences. Study date: the control group was September 2018 to May 2019, and the intervention group was May 1 to July 6, 2020. Country income classification: high-income countries in 2020. Settings: 43 schools in Ontario (n = 20) and Quebec (n = 23). All pre-specified confounders were not adjusted for, and an adjustment model accounting for cluster factors (schools) was used.
Participants	Type of participants: grade 9-12 students (adolescents). Total number of participants: 7653. Age (mean ± SD): not available. Gender: not available.
Interventions	Type of intervention: lockdown. Period of interventions: March to July 2020. Pre-specified co-interventions: not available.
Outcomes	(1) Substance use: vape. This was measured by an online survey, 2-3 months after the lockdown. In the intervention group, the number of follow-up participants was 1949, and the adjusted estimates of monthly use were mean 22.5 (95% CI 12.2, 32.9). In the control group, the number of follow-up participants was 7585, and the adjusted estimates of monthly use were mean 31.7 (95% CI 30, 33.9). The average discrete change between two groups was mean -9.2 (95% CI -19.3, 0.9).
Notes	Country: Canada. Funding source: a research funding agreement from Health Canada (#4500421359; contract awarded to STL). Contact author: Scott T. Leatherdale - sleather@uwaterloo.ca.
Lee et al. (2020) [63]	
Study characteristics	
Methods	A longitudinal study on social role transitions and alcohol use. Key study design feature: time differences. Study date: the control group was January 2020, and the intervention group was April to May 20220. Country income classification: high-income countries in 2020. Setting: Seattle. All pre-specified confounders were not adjusted for, and an adjustment model accounting for cluster factors (individuals) was used.
Participants	Type of participants: a community sample of young adults. Total number of participants: 546. Age (mean ± SD): 25.1 ± 1.9 years. Gender: 342 females/222 males.
Interventions	Type of intervention: mitigation policies (e.g., shelter-in-place). Period of interventions: not available. Pre-specified co-interventions: not available.
Outcomes	(1) Depressive symptom severity. This was measured using the Patient Health Questionnaire-4 – Depression, 1-2 months after the lockdown. The mean (SD) score in the intervention group was 1.86 (1.59), and the score in the control group was 1.63 (1.67). The effect size of the intervention calculated by analysis of a mixed-effects model was estimated to be 1.13 (95% CI 1.03, 1.24; p = 0.013). (2) Anxiety symptom severity. This was measured using the Patient Health Questionnaire-4 – Anxiety, 1-2 months after the lockdown. The mean (SD) score in the intervention group was 2.05 (1.76), and the score in the control group was 2.12 (1.77). The effect size of the intervention calculated by analysis of a mixed-effects model was estimated to be 0.96 (95% CI 0.88, 1.05; p = 0.386).
Notes	Country: United States. Funding source: the National Institute on Alcohol Abuse and Alcoholism (R01AA022087, R01AA027496, and R34AA028074) and the University of Washington Department of Psychiatry and Behavioral Sciences and the Arthur Elzey Research. Contact author: Christine M. Lee - leecm@uw.edu.
Leightley et al. (2021) [64]	
Study characteristics	
Methods	A longitudinal study using data from the Remote Assessment of Disease and Relapse in individuals with Major Depressive Disorder (RADAR-MDD) project. Key study design feature: time differences. Study date: the control group was December 2019 to March 2020, and the intervention group was March to May 2020. Country income classification: high-income countries in 2020. Setting: a multi-center cohort, examining the use of remote measurement technology in monitoring major depressive disorder in the United Kingdom, Spain, and the Netherlands. All pre-specified confounders were not adjusted for, and an adjustment model accounting for cluster factors (individuals) was used.
Participants	Type of participants: participants aged 18 years or older with DSM-5 diagnostic criteria for a diagnosis of nonpsychotic MDD in the past two years and recurrent MDD (adults). Total number: 252. Age (mean ± SD): not available. Gender: 188 females/64 males.
Interventions	Type of intervention: lockdown. Period of interventions: United Kingdom, March 23 to May 11, 2020; Spain, March 14 to May 2, 2020; Netherlands, March 17 to May 11, 2020. Pre-specified co-interventions: not available.
Outcomes	(1) Depressive symptom severity. This was measured using the Patient Health Questionnaire (PHQ-8), immediately and two months after the lockdown. The mean (SD) score in the intervention group was 9.69 (1.66), and the score in the control group was 10.5 (1.45). The estimated mean difference between the two groups calculated by a linear mixed model was -0.18 (95% CI -0.16, 0.24; p = 0.339).
Notes	Country: United Kingdom, Spain, and Netherlands. Funding source: Health Research (NIHR) Biomedical Research Centre and South London and Maudsley NHS Foundation Trust and King’s College London. Contact author: Daniel Leightley - daniel.leightley@kcl.ac.uk.
Liu et al. (2022) [65]	
Study characteristics	
Methods	A longitudinal study. Key study design feature: time differences. Study date: the control group was 2015 to March 2020, and the intervention group was May 2020. Country income classification: high-income countries in 2020. Setting: Southern California. All pre-specified confounders were not adjusted for, and an adjustment model accounting for cluster factors was not used.
Participants	Type of participants: adolescents whose mothers were originally recruited during pregnancy into a longitudinal study of child development. Total number: 175. Age (mean ± SD): 16.01 ± 2.56 years. Gender: 86 females/89 males.
Interventions	Type of intervention: stay-at-home-orders. Period of interventions: March 19 to May 2020. Pre-specified co-interventions: not available.
Outcomes	(1) Depressive symptom severity. This was measured using the Children’s Depression Inventory, two months after the lockdown. Among 89 boys, the mean (SD) score in the intervention group was 3.94 (3.4), and the score in the control group was 3.91 (3.49). The effect size of the intervention calculated by a linear regression model was estimated to be 0.03 (95% CI -0.54, 0.6). Among 86 girls, the mean (SD) score in the intervention group was 5.66 (3.39), and the score in the control group was 4.35 (3.34). The effect size of the intervention calculated by a linear regression model was estimated to be 1.37 (95% CI 0.78, 1.84)
Notes	Country: United States. Funding source: the National Institutes of Health (P50 MH096889). Contact author: Sabrina R. Liu - sabliu@chapman.edu.
Macfarlane et al. (2021) [66]	
Study characteristics	
Methods	A re-surveyed three cohorts of patients with musculoskeletal disease or symptoms. Key study design feature: time differences. Study date: the control group was not available, and the intervention group was July to September 30, 2020. Country income classification: high-income countries in 2020. Setting: United Kingdom. All pre-specified confounders were not adjusted for, and an adjustment model accounting for cluster factors (individuals) was used.
Participants	Type of participants: people with axial spondyloarthritis or psoriatic arthritis and participants in a trial in the United Kingdom who had regional pain and were identified at high risk of developing chronic widespread pain. Total number: 1054. Age (mean ± SD): 59 ± 16.3 years. Gender: 476 females/578 males.
Interventions	Type of intervention: national lockdown. Period of interventions: more than 1 month from March 23, 2020. Pre-specified co-interventions: not available.
Outcomes	(1) Depressive symptom severity. This was measured using the PROMIS depression, 4-7 months after the lockdown. In the intervention group, the number of the follow-up participants were 143, and the mean (SD) score was 51.5 (9.3). In the control group, the number of the follow-up participants were 143, and the mean (SD) score was 51 (9.5). The age-adjusted mean difference between the two groups calculated by a mixed-effects linear regression model was estimated to be 0.6 (95% CI -0.7, 1.8). (2) Anxiety symptom severity. This was measured using the PROMIS anxiety, 4-7 months after the lockdown. In the intervention group, the number of the follow-up participants were 142, and the mean (SD) score was 53 (9.6). In the control group, the number of the follow-up participants were 142, and the mean (SD) score was 51.4 (10.1). The age-adjusted mean difference between the two groups calculated by a mixed-effects linear regression model was estimated to be 1.7 (95% CI 0.5, 2.9). (3) Insomnia symptom severity. This was measured using the Jenkins Sleep Problem Scale score, 4-7 months after the lockdown. In the intervention group, the number of the follow-up participants were 927, and the mean (SD) score was 8.39 (9.3). In the control group, the number of the follow-up participants were 927, and the mean (SD) score was 9.01 (5.52). The age-adjusted mean difference between the two groups calculated by a mixed-effects linear regression model was estimated to be -0.52 (95% CI -0.82, -0.22).
Notes	Country: United Kingdom. Funding source: Versus Arthritis (Grant No.: 20748), the British Society for Rheumatology, Versus Arthritis (MAmMOTH), the British Society for Rheumatology (BSRBR-AS and BSR-PsA), and a Versus Arthritis Foundation Fellowship (Grant No. 21742). Contact author: Gary J. Macfarlane - g.j.macfarlane@abdn.ac.uk.
Mauz et al. (2023) [67]	
Study characteristics	
Methods	A longitudinal study using data from the European Health Interview Survey as part of the study "German Health Update" (GEDA 2019/2020-EHIS) for Germany. Key study design feature: time differences. Study date: the control group was mid-September 2019 to end of December 2019, and the intervention group was mid-September 2020 to the end December 2020. Country income classification: high-income countries in 2020. Setting: Germany. All pre-specified confounders were not adjusted for. An adjustment model accounting for cluster factors was not used, as individuals were not a clustering factor.
Participants	Type of participants: individuals 18 years and older living in Germany. Total number of participants: 26,152. Age: 18-29 years (2425), 30-44 years (4326), 45-64 years (10,305), and 65+ years (9096). Gender: 13,788 females/12,364 males
Interventions	Type of intervention: lockdown. Period of interventions: not available. Pre-specified co-interventions: not available.
Outcomes	(1) Depressive symptom severity. This was measured using the Patient Health Questionnaire-2 (PHQ-2), 6-9 months after the lockdown. The mean (95% CI) score in the intervention group was 1.02 (0.93, 11), and the score in the control group was 0.95 (0.88, 1.01). The effect size of the intervention calculated by analysis of a linear regression model was estimated to be 0.186 (p = 0.254).
Notes	Country: Germany. Funding source: the Federal Ministry of Health (Grant Number: ZMI5-2519FSB402) and the German Research Foundation (Project Number: 458531028). Contact author: Elvira Mauz - MauzE@rki.de.
Meda et al. (2021) [68]	
Study characteristics	
Methods	A longitudinal study using prospective data on students’ mental health in two instances. Key study design feature: time differences. Study date: the control group was October 3-30, 2019, and the intervention group was May 11 to June 21, 2020. Country income classification: high-income countries in 2020. Setting: Padova, Veneto region (Northern Italy), with a population of 200,000, hosting one of the largest universities in the country, with a student population of more than 50,000. All pre-specified confounders were not adjusted for, and an adjustment model accounting for cluster factors (individuals) was used.
Participants	Type of participants: approximately 1000 students from the University of Padova. Total number: 358. Age (mean ± SD): 21.3 ± 2.1 years. Gender: 286 females/72 males.
Interventions	Type of intervention: COVID-19 lockdown. Period of interventions: March 9 to May 4, 2020. Pre-specified co-interventions: not available.
Outcomes	(1) Depressive symptom severity. This was measured using the Beck’s Depression Inventory-2, 2-3 months after the lockdown. In the intervention group, the number of participants was 197, and the mean (SD) score was 13.8 (10.8). In the control group, the number of participants was 197, and the mean (SD) score was 13.5 (9.73). The effect size of the intervention calculated by a generalized linear mixed model was estimated to be 0.01 (95% CI -0.03, 0.03). (2) Anxiety symptom severity. This was measured using the Beck’s Anxiety Inventory, 2-3 months after the lockdown. In the intervention group, the number of participants was 197, and the mean (SD) score was 15 (10.7). In the control group, the number of participants was 197, and the mean (SD) score was 16.1 (11.8). The effect size of the intervention calculated by a generalized linear mixed model was estimated to be -0.088 (95% CI -0.02, 0.02).
Notes	Country: Italy. Funding source: not available. Contact author: Francesco Visioli - francesco.visioli@unipd.it.
Minhas et al. (2021) [69]	
Study characteristics	
Methods	A longitudinal study on alcohol misuse in emerging adults. Key study design feature: time differences. Study date: the control group was October to November 2019, and the intervention group was June 17 to July 1, 2020. Country income classification: high-income countries in 2020. Setting: Hamilton and Ontario. One pre-specified confounder (economic status) was not adjusted for, and an adjustment model accounting for cluster factors (individuals) was used.
Participants	Type of participants: a voluntary community sample of emerging adults (adults). Total number of participants: 473. Age (mean ± SD): intervention group, 23.84 ± 1.29 years; control group, 23.42 ± 1.22 years. Gender: 276 females/197 males.
Interventions	Type of intervention: lockdown. Period of interventions: not available. Pre-specified co-interventions: not available.
Outcomes	(1) Depressive symptom severity. This was measured using the Patient Health Questionnaire-9, three months after the lockdown. The mean (SD) score in the intervention group was 7.54 (6.09), and the score in the control group was 6.76 (5.65). The effect size of the intervention calculated by a linear mixed-effects model was estimated to be 12.78 (p = 0.0004). (2) Anxiety symptom severity. This was measured using the Generalized Anxiety Disorder-7, three months after the lockdown. The mean (SD) score in the intervention group was 6.34 (5.43), and the score in the control group was 5.77 (5.65). The effect size of the intervention calculated by a linear mixed-effects model was estimated to be 10.29 (p = 0.001). (3) Substance use: alcohol. In the intervention group, the number of follow-up participants was 473, and the mean (SD) heavy drinking days per week was 0.49 (1.09). In the control group, the number of follow-up participants was 473, and the mean (SD) days per week was 0.63 (1.09). The coefficient estimated by a linear mixed-effects model between two groups was 6.48 (p = 0.01).
Notes	Country: Canada. Funding source: Canadian Institutes of Health Research. Contact author: James MacKillop - jmackill@mcmaster.ca
Moya et al. (2021) [70]	
Study characteristics	
Methods	A longitudinal study using data from a psychosocial support program. Key study design feature: time differences. Study date: the control group was July 2019, and the intervention group was April 8-29, 2020. Country income classification: middle- or low-income countries in 2020. Setting: Tumaco, a municipality in the Pacific coast of Colombia. One pre-specified confounder (economic status) was adjusted for, and an adjustment model accounting for cluster factors was used.
Participants	Type of participants: primary caregivers who took part in a cluster-randomized trial of Semillas de Apego, a psychosocial group program based on the Child-Parent Psychotherapy (adults). Total number of participants: 1376 (803 intervention group and 573 control group). Age (mean ± SD): intervention group, 29.05 ± 9.24 years; control group, 29.07 ± 9.3 years. Gender: intervention group, 781 females/22 males; control group, 538 females/35 males.
Interventions	Type of intervention: national lockdown. Period of interventions: not available. Pre-specified co-interventions: not available.
Outcomes	(1) Depression symptom severity. This was measured using the T score of Symptoms Checklist-90 – Revised, 2-5 weeks after the lockdown. The mean (SD) score in the intervention group was 59.73 (6.93), and the score in the control group was 59.91 (7.07). The coefficient of the intervention calculated by a regression model was estimated to be 0.05 (95% CI 0.005, 0.091; p = 0.03). (2) Anxiety symptom severity. This was measured using the T score of Symptoms Checklist-90 – Revised, 2-5 weeks after the lockdown. The mean (SD) score in the intervention group was 57.01 (6.96), and the score in the control group was 57.21 (6.74). The coefficient of the intervention calculated by a regression model was estimated to be 0.14 (95% CI 0.1, 0.174; p < 0.0001).
Notes	Country: Colombia. Funding source: Saving Brains–Grand Challenges Canada, Fundación Éxito, Fundación FEMSA, United Way Colombia, Universidad de los Andes. Contact author: Andrés Moya - a.moya@uniandes.edu.co.
Murphy et al. (2023) [71]	
Study characteristics	
Methods	A longitudinal family study of three generations at high and low risk for depression. Key study design feature: time differences. Study date: the control group was September 2017 to March 2020, and the intervention group was September 2020 to February 2021. Country income classification: high-income countries in 2020. Setting: a US cohort, followed for up to 38 years with direct clinical interviews on themselves and their relatives. One pre-specified confounder (relationship status) was not adjusted for, and an adjustment model accounting for cluster factor (individuals) was used.
Participants	Type of participants: the first was recruited from an outpatient clinic and included probands with moderate-to-severely impairing major depressive disorder (MDD) but no schizophrenia, antisocial personality disorder, bipolar disorder, or primary substance use disorder. The second was selected from an epidemiologic sample in the same community and had no lifetime history of psychiatric illness, as confirmed through several interviews. Second (G2)- and third (G3)-generation offspring of probands with and without MDD. Total number of participants: 190 (45 no psychiatric history, 66 only-past psychiatric history, and 79 recent psychiatric history). Age (mean ± SD): intervention group, 39.3 ± 15.7 years (no psychiatric history), 49 ± 13.9 years (only-past psychiatric history), and 39.8 ± 14.6 years (recent psychiatric history). Gender: 25 females/20 males (no psychiatric history), 34 females/32 males (only-past psychiatric history), and 49 females/30 males (recent psychiatric history).
Interventions	Type of intervention: lockdown and social distancing. Period of interventions: not available. Pre-specified co-interventions: not available.
Outcomes	(1) Depressive symptom severity. This was measured using the IDAS II Symptom Measures – Depression, 6-11 months after the lockdown. Among the no psychiatric history participants, the mean (SD) score in the intervention group was 32.8 (1.79), and the score in the control group was 31.9 (1.79). Among the no psychiatric history participants, the mean (SD) score in the intervention group was 36.4 (1.53), and the score in the control group was 36.7 (1.47). Among the no psychiatric history participants, the mean (SD) score in the intervention group was 41.7 (1.43), and the score in the control group was 47.6 (1.43). The effect of the intervention calculated by a generalized linear mixed model was estimated to be F(2, 372) 8.0 (p < 0.001). (2) Anxiety symptom severity. This was measured using the IDAS II Symptom Measures – Anxiety, 6-11 months after the lockdown. Among the no psychiatric history participants, the mean (SD) score in the intervention group was 7.2 (0.46), and the score in the control group was 7.4 (0.46). Among the no psychiatric history participants, the mean (SD) score in the intervention group was 8 (0.36), and the score in the control group was 8.4 (0.41). Among the no psychiatric history participants, the mean (SD) score in the intervention group was 9.4 (0.36), and the score in the control group was 10.8 (0.36). The effect of the intervention calculated by the analysis of covariance (ANCOVA) was estimated to be F(2, 371) 2.9 (p < 0.1).
Notes	Country: United States. Funding source: the National Institute of Mental Health (R01 MH-036197, MMW, JP), the John J. Templeton Foundation (MMW), and a Columbia University Depression Center award (AT). Contact author: Ardesheer Talati - adi.talati@nyspi.columbia.edu.
Overbeck et al. (2021) [72]	
Study characteristics	
Methods	A cross-sectional study using data from two cohorts of pregnant women. Key study design feature: time differences. Study date: the control group was April 2015 to August 2016, and the intervention group was April 8 to May 6, 2020. Country income classification: high-income countries in 2020. Setting: 70 general practitioner clinics from two of five Danish regions recruited pregnant women from urban, sub-urban, and rural areas. Two pre-specified confounders (occupation and relationship status) were adjusted for, and an adjustment model accounting for cluster factors (individuals) was used.
Participants	Type of participants: pregnant women (adults). Total number: 1758 (330 intervention group and 1428 control group). Age (mean ± SD): intervention group, ≤25 years (28), 26-30 years (122), 31-35 years (120), and >35 years (60); and control group, ≤25 years (180), 26-30 years (491), 31-35 years (480), and >35 years (277). Gender: intervention group, 330 females; control group, 1428 females.
Interventions	Type of intervention: COVID-19 lockdown. Period of interventions: March 12 to mid-April 2020. Pre-specified co-interventions: not available.
Outcomes	(1) Depressive symptom severity. This was measured using the Major Depression Inventory (MDI), 1-2 months after the lockdown. The mean (SD) score in the intervention group was 9.5 (7.5), and the score in the control group was 10.7 (7.6). The adjusted mean difference between the two groups calculated by a multivariable linear regression model was estimated to be -0.57 (95% CI -1.62, 0.48; p = 0.287). (2) Anxiety symptom severity. This was measured using the Anxiety Symptom Scale (ASS), 1-2 months after the lockdown. In the first trimester, in the intervention group, the number of participants was 33, and the mean (SD) score was 7.0 (6.1). In the control group, the number of participants was 1428, and the mean score was 3.4 (4.6). The adjusted mean difference between the two groups calculated by a multivariable linear regression model was estimated to be 4.0 (95% CI 2.37, 5.64; p < 0.0001). In the second trimester, in the intervention group, the number of participants was 219, and the mean (SD) score was 4.0 (4.3). In the control group, the number of participants was 1343, and the mean score was 3.1 (4.3). The adjusted mean difference between the two groups calculated by a multivariable linear regression model was estimated to be 0.6 (95% CI -0.15, 1.36; p = 0.1165). In the third trimester, in the intervention group, the number of participants was 78, and the mean (SD) score was 4.9 (5.0). In the control group, the number of participants was 1326, and the mean score was 2.9 (3.9). The adjusted mean difference between the two groups calculated by a multivariable linear regression model was estimated to be 2.05 (95% CI 0.97, 3.13; p = 0.0002).
Notes	Country: Denmark. Funding source: TRYGfonden (grant number 125227) and the quality and continuing education committee for general practice in the Capital Region (grant number 19035774). Contact author: Gritt Overbeck - not available.
Pelham et al. (2022) [73]	
Study characteristics	
Methods	A longitudinal study using data from a prospective cohort (the National Consortium on Alcohol & Neurodevelopment in Adolescence (NCANDA) study). Key study design feature: time differences. Study date: the control group was 2016 to March 19, 2020, and the intervention group was December 7-24, 2020. Country income classification: high-income countries in 2020. Setting: five study sites across the United States: Duke University, University of Pittsburgh Medical Center, Oregon Health & Science University, University of California San Diego, and SRI International. All pre-specified confounders were not adjusted for, and an adjustment model accounting for cluster factors (individuals) was used.
Participants	Type of participants: ages 12-21 years old in the NCANDA study. Total number: 494 (213 intervention group and 281 control group). Age (range): intervention group, 18.8-22.4 years; control group, 18.8-22.4 years Gender: intervention group, 48% females/52% males; control group, 53% females/47% males.
Interventions	Type of intervention: stay-at-home order. Period of interventions: not available. Pre-specified co-interventions: not available.
Outcomes	(1) Substance use: alcohol. This was measured by an online survey, nine months after the intervention. Among those with past-month drinking, in the intervention group, the mean (SD) number of drinks on a typical drinking day was 3.24 (3.2). In the control group, the mean (SD) number was 3.32 (2.34). The coefficient of the intervention estimated by a models using generalized estimating equations was -0.08 (SE 0.25; p = 0.73). (2) Substance use: tobacco. This was measured by an online survey, nine months after the intervention. Among those with past-month nicotine product use, in the intervention group, the mean (SD) number of days used was 12.3 (21.3). In the control group, the mean (SD) number was 13.3 (23). The coefficient of the intervention estimated by a models using generalized estimating equations was -1 (SE 1.78; p = 0.58).
Notes	Country: United States. Funding source: NIH funding (AA021681, AA021690, AA021691, AA021692, AA021695, AA021696, AA021696-07S1, AA021697, AA028840, AA030197, and DA055935). Contact author: William E. Pelham III - wpelham@ucsd.edu.
Rimfeld et al. (2022) [74]	
Study characteristics	
Methods	A longitudinal study using data from the Twins Early Development Study. Key study design feature: time differences. Study date: the control group was 2018, and the intervention group was March 2021. Country income classification: high-income countries in 2020. Setting: England and Wales. All pre-specified confounders were not adjusted for, and an adjustment model accounting for cluster factors was not used.
Participants	Type of participants: unrelated individuals, complete monozygotic twin pairs, and complete dizygotic twin pairs. Total number of participants: 4773. Age (mean ± SD): intervention group, not available; control group, 22.27 ± 0.9 years. Gender: not available.
Interventions	Type of intervention: lockdown. Period of interventions: not available. Pre-specified co-interventions: not available.
Outcomes	(1) Depressive symptom severity. This was measured using the Short Mood and Feelings Questionnaire, 11 months after the lockdown. In the intervention group, the number of participants was 1940, and the mean (SD) score was 4.46 (4.09). In the control group, the number of participants was 4769, and the mean (SD) score was 4.47 (4.13). The effect of the intervention calculated by a multivariate analysis of variance (MANOVA) was estimated to be F = 0.2. (2) Anxiety symptom severity. This was measured using the Severity Measure for Generalized Anxiety Disorder, 11 months after the lockdown. In the intervention group, the number of participants was 1940, and the mean (SD) score was 8.98 (7.78). In the control group, the number of participants was 4250, and the mean (SD) score was 7.46 (7.5). The effect of the intervention calculated by a multivariate analysis of variance (MANOVA) was estimated to be F = 10.32 (p < 0.001).
Notes	Country: England and Wales. Funding source: the UK Medical Research Council (MR/ M021475/1 and previously G0901245) with additional support from the US National Institutes of Health (AG046938), Sir Henry Wellcome Postdoctoral Fellowship (213514/ Z/18/Z), Jacobs Foundation fellowship, the NIHR Biomedical Research Centre at South London and Maudsley and Guys and St Thomas NHS Foundation Trusts, capital equipment grants from the Maudsley Charity (grant ref. 980), Guys & St Thomas Charity (TR130505), the NIHR Maudsley Biomedical Research Centre at South London and Maudsley NHS Foundation Trust and King’s College London, and the Wellcome Trust (213514/Z/18/Z). Contact author: Kaili Rimfeld - kaili.rimfeld@kcl.ac.uk
Romdhani et al. (2022) [75]	
Study characteristics	
Methods	A cross-sectional, global, and web-based questionnaire. Key study design feature: time differences. Study date: control group, not available; the intervention group was July 8 to September 30, 2020. Country income classification: not available. Setting: a multilingual cross-survey among athletes from 49 countries. All pre-specified confounders were not adjusted for, and an adjustment model accounting for cluster factors was not used.
Participants	Type of participants: (i) ≥18 years of age, (ii) classified as an athlete (competing at any given level: individual or team sport), and (iii) had experienced a period of lockdown for at least two weeks. Total number of participants: 3911. Age (mean ± SD): intervention group, 25.06 ± 8.9 years. Gender: 1764 females/2106 males
Interventions	Type of intervention: lockdown. Period of interventions: not available. Pre-specified co-interventions: not available.
Outcomes	(1) Insomnia symptom severity. This was measured using the Pittsburgh Sleep Quality index, 3-5 months after the lockdown. The mean (SD) score in the intervention group was 5.85 (3.16), and the score in the control group was 4.33 (2.49). The mean difference calculated by a multiple linear regression model was estimated to be 1.51 (95% CI 1.42, 1.61; p < 0.001).
Notes	Country: 49 countries. Funding source: not available. Contact author: Mohamed Romdhani - romdhaniroma@gmail.com.
Sacre et al. (2021) [76]	
Study characteristics	
Methods	A cross-sectional study using data from the Progression of Diabetic Complications (PREDICT) cohort. Key study design feature: time differences. Study date: the control group was 2018-2020, and the intervention group was April 30 to June 30, 2020. Country income classification: high-income countries in 2020. Settings: a 10-km radius of the Baker Heart and Diabetes Institute (Melbourne, Australia). All pre-specified confounders were not adjusted for, and an adjustment model accounting for cluster factors (individuals) was used.
Participants	Type of participants: adults (aged 18-80 years) with type 2 diabetes. Total number: 470. Age (mean ± SD): 66 ± 9 years. Gender: 146 females/324 males
Interventions	Type of intervention: lockdown. Period of interventions: May to June 2020 (more than one month). Pre-specified co-interventions: not available.
Outcomes	(1) Depressive symptom severity. This was measured using the 8-Item Patient Health Questionnaire, immediately and two months after the lockdown. The mean (SD) score in the intervention group was 2.7 (3.3), and the score in the control group was 2.7 (3.3). The effect of the intervention calculated by a multilevel regression model was p = 0.98. (2) Anxiety symptom severity. This was measured using the 7-Item Generalized Anxiety Disorder, immediately and two months after the lockdown. The mean (SD) score in the intervention group was 2 (3.2), and the score in the control group was 2.2 (3.2). The effect of the intervention calculated by a multilevel regression model was p = 0.46.
Notes	Country: Australia. Funding source: La Trobe University, the Ernest Heine Family Foundation–Sydney, Boehringer Ingelheim, the National Health and Medical Research Council of Australia (APP1107361 to DJM and APP1173952 to JES), and the State Government of Victoria Operational Infrastructure Support Program. Contact author: Julian W. Sacre - julian.sacre@baker.edu.au.
Shoshani et al. (2021) [77]	
Study characteristics	
Methods	A cross-sectional study. Key study design feature: time differences. Study date: the control group was September 2019, and the intervention group was May 2020. Country income classification: high-income countries in 2020. Settings: 38 schools in three representative geographical areas. All pre-specified confounders were not adjusted for, and an adjustment model accounting for cluster factors was not used.
Participants	Type of participants: Grade 5-11 students aged 11.1-17 at the beginning of the study from six schools. Total number of participants: 1537. Age (mean ± SD): 13.97 ± 1.21 years. Gender: 799 females/738 males.
Interventions	Type of intervention: lockdown. Period of interventions: not available Pre-specified co-interventions: not available.
Outcomes	(1) Depressive symptom severity. This was measured using the Brief Symptom Inventory 18 – Depression, two months after the lockdown. The mean (SD) score in the intervention group was 7.59 (5.25), and the score in the control group was 6.14 (4.73). The effect of the intervention calculated by a repeated-measures multivariate analysis of variance was estimated to be F = 141.22 (p < 0.001). (2) Anxiety symptom severity. This was measured using the Brief Symptom Inventory 18 – Anxiety, two months after the lockdown. The mean (SD) score in the intervention group was 5.24 (3.14), and the score in the control group was 3.93 (2.68). The effect of the intervention calculated by a repeated-measures multivariate analysis of variance was estimated to be F = 230.93 (p < 0.001).
Notes	Country: Israel. Funding source: not available. Contact author: Anat Shoshani - ashoshani@ idc.ac.il.
Tanaka et al. (2021) [78]	
Study characteristics	
Methods	Key study design feature: time differences. Study date: the control group was November 2016 to January 2020, and the intervention group was July to October 2020. Country income classification: high-income countries in 2020. Setting: whole Japan region. All pre-specified confounders were not adjusted for. An adjustment model accounting for cluster factors was not used, as individuals were not a clustering factor.
Participants	General population in Japan. Age and gender: not available.
Interventions	Type of intervention: state of emergency. Periods of intervention: not available. Pre-specified co-interventions: public subsidies to wages.
Outcomes	(1) Suicide. This was measured using a city-by-month-level dataset covering the entire Japanese population of more than 120 million people. Follow-up period: 3-6 months. The proportion of suicide in the intervention period was 14.6 per million, and the proportion in the control period was 12.8 per million. The adjusted incidence rate ratio between two period was 1.16 (95% CI 1.11-1.21).
Notes	Country: Japan. Funding source: a postdoctoral fellowship of the Japan Society for the Promotion of Science (no. 20J00394) and the Murata Science Foundation. Contact author: Shohei Okamoto - sokamoto@tmig.or.jp.
van der Velden et al. (2022) [79]	
Study characteristics	
Methods	A population-based study. Key study design feature: time differences. Study date: the control group was March 2019, and the intervention group was March 2021. Country income classification: high-income countries in 2020. Settings: the Longitudinal Internet Studies for the Social Sciences (LISS) panel, based on a traditional probability sample drawn from the Dutch population register of 16 years and older by Statistics Netherlands. All pre-specified confounders were not adjusted for, and an adjustment model accounting for cluster factors (individuals) was used.
Participants	Type of participants: victims of violence, accidents, and serious threats in the Netherlands (adults). Total number of participants: 740 (319 intervention group and 421 control group). Age (mean ± SD): intervention group, 18-34 years (102), 35-49 years (8)7, 50-64 years (80), and 65 and older (50); control group, 18-34 years (116), 35-49 years (121), 50-64 years (107), and 65 and older (77). Gender: intervention group, 141 females/178 males; control group, 215 females/206 males
Interventions	Type of intervention: lockdown. Period of interventions: not available. Pre-specified co-interventions: not available.
Outcomes	(1) Depressive symptom severity. This was measured using the 5-Item Mental Health Inventory, 12 months after the lockdown. The mean (SD) score in the intervention group was 64.1 (19.8), and the score in the control group was 69.6 (19.4). The effect of the intervention calculated by a mixed-effects model was estimated to be F(1, 739) 11.228 (p = 0.001). (2) Post-traumatic stress disorder symptom severity. This was measured using the 8-Item Version of the PTSD Checklist for DSM-5 (PCL-5), 12 months after the lockdown. The mean (SD) score in the intervention group was 7.4 (7.9), and the score in the control group was 5.6 (6.8). The effect of the intervention calculated by a mixed-effects model was estimated to be F(1, 739) 7.636 (p = 0.006).
Notes	Country: Netherlands. Funding source: Fonds Slachtofferhulp, Netherlands (50006/VICTIMS). Contact author: Peter G. van der Velden - pg.vandervelden@tilburguniversity.edu.
van den Besselaar et al. (2021) [80]	
Study characteristics	
Methods	A longitudinal study using data from the Longitudinal Aging Study Amsterdam (LASA), which was an ongoing prospective cohort study. Key study design feature: time differences. Study date: the control group was 2018-2019, and the intervention group was June 2020. Country income classification: high-income countries in 2020. Settings: a representative sample in the Netherlands All pre-specified confounders were not adjusted for, and an adjustment model accounting for cluster factors (individuals) was used.
Participants	Type of participants: older adults (aged 62-102 years) who participated in the LASA study in the Netherlands. Total number: 2052 (1068 intervention group and 984 control group). Age (mean ± SD): intervention group, 73.8 ± 7.5 years; control group, not available. Gender: intervention group; 52.8% females/47.2% males; control group; not available.
Interventions	Type of intervention: social distancing measures. Period of interventions: March to mid-May 2020. Pre-specified co-interventions: not available.
Outcomes	(1) Depressive symptom severity. This was measured using the Center for Epidemiologic Studies Depression Scale (CES-D), Short Version (10-item scale), three months after the lockdown. The mean (SD) score in the intervention group was 5.92 (4.11), and the score in the control group was 4.49 (4.05). The effect size of the intervention calculated by a linear mixed model was estimated to be 1.37 (95% 1.12, 1.62; p < 0.05). (2) Anxiety symptom severity. This was measured using the Hospital Anxiety Depression Scale - Anxiety subscale (HADS-A), three months after the lockdown. The mean (SD) score in the intervention group was 3.35 (2.99), and the score in the control group was 2.58 (2.7). The effect size of the intervention calculated by a linear mixed model was estimated to be 0.74 (95% 0.56, 0.94; p < 0.05).
Notes	Country: Netherlands. Funding source: the Dutch Ministry of Health, Welfare and Sport. Contact author: Emiel O. Hoogendijk - e.hoogendijk@amsterdamumc.nl.
Yang et al. (2021) [81]	
Study characteristics	
Methods	A longitudinal study. Key study design feature: time differences. Study date: the control group was December 2019, and the intervention group was June 2020. Country income classification: middle- or low-income countries in 2020. Setting: Wenzhou Medical University in Wenzhou City, Zhejiang Province, China. All pre-specified confounders were not adjusted for, and an adjustment model accounting for cluster factors was not used.
Participants	Type of participants: first-year college students. Total number: 195. Age (mean ± SD): not available. Gender: 114 females/81 males
Interventions	Type of intervention: lockdown. Period of interventions: not available. Pre-specified co-interventions: not available.
Outcomes	(1) Depressive symptom severity. This was measured using the Chinese version of the 20-Item Center for Epidemiologic Studies Depression Scale, six months after the lockdown. The mean (SD) score in the intervention group was 19.08 (6.63), and the score in the control group was 15.93 (9.97). The effect size of the intervention calculated by a linear regression model was estimated to be -0.05.
Notes	Country: China. Funding source: Youth Project of National Social Science Fund of China (No. CBA170257). Contact author: Guohua Zhang - zghcnu@wmu.edu.cn.
Zijlmans et al. (2023) [82]	
Study characteristics	
Methods	Key study design feature: time differences. Study date: the control group was 2017-2018, and the intervention group was March to April 2021. Country income classification: high-income countries in 2020. Setting: samples representative of the Dutch population using an online panel agency. All pre-specified confounders were not adjusted for, and an adjustment model accounting for cluster factors was not used.
Participants	Type of participants: children aged 8-18. Total number: 2401. Age (mean ± SD): intervention group, 13.6 ± 3.3 years; control group, 13.1 ± 3.1 years. Gender: intervention group, 47.9% females/52.1% males; control group, 49.7% females/50.3% males.
Interventions	Type of intervention: lockdown. Period of interventions: March 15 to May 11, 2020. Pre-specified co-interventions: not available.
Outcomes	(1) Depressive symptom severity. This was measured using the Patient‐Reported Outcomes Measurement Information System (PROMIS) - Depressive Symptoms v2.0, 12 months after the lockdown. In the intervention group, the number of participants was 409, and the mean (SD) score was 0.5 (1.01). In the control group, the number of participants was 1319, and the mean (SD) score was 0 (1.09). The effect of the intervention calculated by analysis of covariance (ANCOVA) was estimated to be F = 74.05 (p < 0.001). (2) Anxiety symptom severity. This was measured using the Patient‐Reported Outcomes Measurement Information System (PROMIS) - Anxiety v2.0, 12 months after the lockdown. In the intervention group, the number of participants was 410, and the mean (SD) score was 0.65 (1.01). In the control group, the number of participants was 1319, and the mean (SD) score was 0 (1.09). The effect of the intervention calculated by analysis of covariance (ANCOVA) was estimated to be F = 129.47 (p < 0.001). (3) Insomnia symptom severity. This was measured using the Patient‐Reported Outcomes Measurement Information System (PROMIS) - Sleep-Related Impairment v1.0, 12 months after the lockdown. In the intervention group, the number of participants was 408, and the mean (SD) score was 0.31 (1.21). In the control group, the number of participants was 527, and the mean (SD) score was -0.05 (1.15). The effect of the intervention calculated by analysis of covariance (ANCOVA) was estimated to be F = 27.04 (p < 0.001).
Notes	Country: Netherlands. Funding source: Netherlands Organization for Health Research and Development; Nederlandse Organisatie voor Wetenschappelijk Onderzoek, Grant/ Award Number: 480‐15‐001/674; European Research Council consolidator grant, Grant/Award Number: 771057; ZonMw, Grant/Award Number: 50‐56300‐98‐973; Stichting Steun Emma Kinderziekenhuis; Zorginstituut Nederland. Contact author: Josjan Zijlmans - j.zijlmans@amsterdamumc.nl.

Supplementary material 6 

**Table 9:** Detailed assessment of the risk of bias for depressive symptom severity

Study	Bias due to confounding	Bias in the selection of participants for the study	Bias in the classification of intervention	Bias due to deviations from intended intervention(s)	Bias due to missing data	Bias in the measurement of outcomes	Bias in the selection of the reported results	Overall risk of bias
Bartlett et al. (2021) [45]	Serious risk	Serious risk	Low risk	Low risk	Serious risk	Moderate risk	Low risk	Serious risk of bias
	Two important confounding domains were controlled.	The start of follow-up and the start of the intervention did not coincide, and adjustment techniques were not used.	The intervention in this study was implemented at the population level.	Deviations from the intended intervention did not occur.	Among the total participants (4282), analyses were conducted on 1671 participants in both groups due to missing data, and the robustness of the analyses was not assessed.	Due to population-level interventions, outcome assessors were not blinded.	The outcomes of interest and the effect estimates were not reported across multiple measurements or analyses.	
Bennett et al. (2022) [46]	Critical risk	Serious risk	Low risk	Low risk	Low risk	Moderate risk	Low risk	Critical risk of bias
	All important confounding domains were not controlled.	The start of follow-up and the start of the intervention did not coincide, and adjustment techniques were not used.	The intervention in this study was implemented at the population level.	Deviations from the intended intervention did not occur.	There were no missing data.	Due to population-level interventions, outcome assessors were not blinded.	The outcomes of interest and the effect estimates were not reported across multiple measurements or analyses.	
Boekhorst et al. (2021) [48]	Serious risk	Serious risk	Low risk	Low risk	Moderate risk	Moderate risk	Moderate risk	Serious risk of bias
	Only one important confounding domain was controlled.	The start of follow-up and the start of the intervention did not coincide, and adjustment techniques were not used.	The intervention in this study was implemented at the population level.	Deviations from the intended intervention did not occur.	In both groups, a small proportion of participants were excluded from the analyses due to missing data, and the reasons for exclusion were probably similar.	Due to population-level interventions, outcome assessors were not blinded.	In this study, there was no pre-registered protocol, and multiple effect estimates were reported.	
Bouter et al. (2023) [49]	Critical risk	Serious risk	Low risk	Low risk	Low risk	Moderate risk	Moderate risk	Critical risk of bias
	All important confounding domains were not controlled.	The start of follow-up and the start of the intervention did not coincide, and adjustment techniques were not used.	The intervention in this study was implemented at the population level.	Deviations from the intended intervention did not occur.	There were no missing data.	Due to population-level interventions, outcome assessors were not blinded.	In this study, there was no pre-registered protocol, and multiple effect estimates were reported.	
Cohen et al. (2021) [54]	Serious risk	Serious risk	Low risk	Low risk	Low risk	Moderate risk	Low risk	Serious risk of bias
	Only one important confounding domain was controlled.	The start of follow-up and the start of the intervention did not coincide, and adjustment techniques were not used.	The intervention in this study was implemented at the population level.	Deviations from the intended intervention did not occur.	There were no missing data.	Due to population-level interventions, outcome assessors were not blinded.	The outcomes of interest and the effect estimates were not reported across multiple measurements or analyses.	
Dunn et al. (2021) [56]	Critical risk	Serious risk	Low risk	Low risk	Serious risk	Moderate risk	Low risk	Critical risk of bias
	All important confounding domains were not controlled.	The start of follow-up and the start of the intervention did not coincide, and adjustment techniques were not used.	The intervention in this study was implemented at the population level.	Deviations from the intended intervention did not occur.	The analysis included 45 participants from the intervention group out of the total 48 participants, with the exclusion due to missing data. Robustness of the results was not confirmed in the analyses.	Due to population-level interventions, outcome assessors were not blinded.	The outcomes of interest and the effect estimates were not reported across multiple measurements or analyses.	
Hausman et al. (2022) [58]	Critical risk	Serious risk	Low risk	Low risk	Serious risk	Moderate risk	Low risk	Critical risk of bias
	All important confounding domains were not controlled.	The start of follow-up and the start of the intervention did not coincide, and adjustment techniques were not used.	The intervention in this study was implemented at the population level.	Deviations from the intended intervention did not occur.	The analysis included 170 participants from the intervention group out of the total 189 participants, with the exclusion due to missing data. Robustness of the results was not confirmed in the analyses conducted.	Due to population-level interventions, outcome assessors were not blinded.	The outcomes of interest and the effect estimates were not reported across multiple measurements or analyses.	
Kekäläinen et al. (2021) [59]	Serious risk	Serious risk	Low risk	Low risk	Serious risk	Moderate risk	Moderate risk	Serious risk of bias
	This study was needed to assess baseline confounding factors, yet it controlled for only two of the three important domains of confounding.	The start of follow-up and the start of the intervention did not coincide, and adjustment techniques were not used.	The intervention in this study was implemented at the population level.	Deviations from the intended intervention did not occur.	The analysis included 264 participants from the intervention group out of the total 358 participants, with the exclusion due to missing data. Robustness of the results was not confirmed in the analyses conducted.	Due to population-level interventions, outcome assessors were not blinded.	In this study, there was no pre-registered protocol, and multiple effect estimates were reported.	
Koenders et al. (2021) [60]	Critical risk	Serious risk	Low risk	Low risk	Serious risk	Moderate risk	Low risk	Critical risk of bias
	All important confounding domains were not controlled, and cluster-level confounding was not accounted for.	The start of follow-up and the start of the intervention did not coincide, and adjustment techniques were not used.	The intervention in this study was implemented at the population level.	Deviations from the intended intervention did not occur.	In the intervention group, more than 20 percent of the participants (8 out of 36) were excluded due to missing data, and the robustness of the analyses was not confirmed.	Due to population-level interventions, outcome assessors were not blinded.	The outcomes of interest and the effect estimates were not reported across multiple measurements or analyses.	
Koenig et al. (2023) [61]	Serious risk	Serious risk	Low risk	Low risk	Low risk	Moderate risk	Low risk	Serious risk of bias
	Only one important confounding domain was controlled.	The start of follow-up and the start of the intervention did not coincide, and adjustment techniques were not used.	The intervention in this study was implemented at the population level.	Deviations from the intended intervention did not occur.	There were no missing data.	Due to population-level interventions, outcome assessors were not blinded.	The outcomes of interest and the effect estimates were not reported across multiple measurements or analyses.	
Lee et al. (2020) [63]	Critical risk	Serious risk	Low risk	Low risk	Low risk	Moderate risk	Low risk	Critical risk of bias
	All important confounding domains were not controlled.	The start of follow-up and the start of the intervention did not coincide, and adjustment techniques were not used.	The intervention in this study was implemented at the population level.	Deviations from the intended intervention did not occur.	There were no missing data.	Due to population-level interventions, outcome assessors were not blinded.	The outcomes of interest and the effect estimates were not reported across multiple measurements or analyses.	
Leightley et al. (2021) [64]	Critical risk	Low risk	Low risk	Low risk	Moderate risk	Moderate risk	Low risk	Critical risk of bias
	All important confounding domains were not controlled.	This study is a secondary analysis of an ongoing cohort study, and study participants were not selected based on characteristics observed after the start of the intervention. In addition, the start of follow-up and the start of intervention coincided.	The intervention in this study was implemented at the population level.	Deviations from the intended intervention did not occur.	In both groups, several participants were excluded due to incomplete data; however, the robustness of the analyses was not assessed.	Due to population-level interventions, outcome assessors were not blinded.	The outcomes of interest and the effect estimates were not reported across multiple measurements or analyses.	
Liu et al. (2022) [65]	Critical risk	Serious risk	Low risk	Low risk	Low risk	Moderate risk	Low risk	Critical risk of bias
	All important confounding domains were not controlled, and cluster-level confounding was not accounted for.	The start of follow-up and the start of the intervention did not coincide, and adjustment techniques were not used.	The intervention in this study was implemented at the population level.	Deviations from the intended intervention did not occur.	There were no missing data.	Due to population-level interventions, outcome assessors were not blinded.	The outcomes of interest and the effect estimates were not reported across multiple measurements or analyses.	
Macfarlane et al. (2021) [66]	Critical risk	Serious risk	Low risk	No information	No information	Moderate risk	Low risk	Critical risk of bias
	All important confounding domains were not controlled.	The start of follow-up and the start of the intervention did not coincide, and adjustment techniques were not used.	The intervention in this study was implemented at the population level.	There was no information on deviations from the intended intervention.	There was no information on missing data.	Due to population-level interventions, outcome assessors were not blinded.	The outcomes of interest and the effect estimates were not reported across multiple measurements or analyses.	
Mauz et al. (2023) [67]	Critical risk	Serious risk	Low risk	Low risk	No information	Moderate risk	Low risk	Critical risk of bias
	All important confounding domains were not controlled.	The start of follow-up and the start of the intervention did not coincide, and adjustment techniques were not used.	The intervention in this study was implemented at the population level.	Deviations from the intended intervention did not occur.	There was no information on missing data.	Due to population-level interventions, outcome assessors were not blinded.	The outcomes of interest and the effect estimates were not reported across multiple measurements or analyses.	
Meda et al. (2021) [68]	Critical risk	Serious risk	Low risk	Low risk	Moderate risk	Moderate risk	Low risk	Critical risk of bias
	All important confounding domains were not controlled.	The start of follow-up and the start of the intervention did not coincide, and adjustment techniques were not used.	The intervention in this study was implemented at the population level.	Deviations from the intended intervention did not occur.	In both groups, 161 out of 358 participants were excluded due to incomplete data, and the robustness of the analyses was not assessed.	Due to population-level interventions, outcome assessors were not blinded.	The outcomes of interest and the effect estimates were not reported across multiple measurements or analyses.	
Minhas et al. (2021) [69]	Serious risk	Serious risk	Low risk	Low risk	Low risk	Moderate risk	Low risk	Serious risk of bias
	Only one important confounding domain was controlled.	The start of follow-up and the start of the intervention did not coincide, and adjustment techniques were not used.	The intervention in this study was implemented at the population level.	Deviations from the intended intervention did not occur.	There were no missing data.	Due to population-level interventions, outcome assessors were not blinded.	The outcomes of interest and the effect estimates were not reported across multiple measurements or analyses.	
Moya et al. (2021) [70]	Serious risk	Serious risk	Low risk	Low risk	Low risk	Moderate risk	Low risk	Serious risk of bias
	Only one important confounding domain was controlled.	The start of follow-up and the start of the intervention did not coincide, and adjustment techniques were not used.	The intervention in this study was implemented at the population level.	Deviations from the intended intervention did not occur.	There were no missing data.	Due to population-level interventions, outcome assessors were not blinded.	The outcomes of interest and the effect estimates were not reported across multiple measurements or analyses.	
Murphy et al. (2023) [71]	Serious risk	Serious risk	Low risk	Low risk	Low risk	Moderate risk	Low risk	Serious risk of bias
	Only one important confounding domain was controlled.	The start of follow-up and the start of the intervention did not coincide, and adjustment techniques were not used.	The intervention in this study was implemented at the population level.	Deviations from the intended intervention did not occur.	There were no missing data.	Due to population-level interventions, outcome assessors were not blinded.	The outcomes of interest and the effect estimates were not reported across multiple measurements or analyses.	
Overbeck 2021 [72]	Serious risk	Serious risk	Low risk	Low risk	Serious risk	Moderate risk	Low risk	Serious risk of bias
	This study was needed to assess baseline confounding factors, yet it controlled for only two of the three important domains of confounding.	The start of follow-up and the start of the intervention did not coincide, and adjustment techniques were not used.	The intervention in this study was implemented at the population level.	Deviations from the intended intervention did not occur.	In the intervention group, 77 out of 407 participants were excluded due to missing data. In contrast, the control group had no missing data.	Due to population-level interventions, outcome assessors were not blinded.	The outcomes of interest and the effect estimates were not reported across multiple measurements or analyses.	
Rimfeld et al. (2022) [74]	Critical risk	Serious risk	Low risk	Low risk	Low risk	Moderate risk	Low risk	Critical risk of bias
	All important confounding domains were not controlled, and cluster-level confounding was not accounted for.	The start of follow-up and the start of the intervention did not coincide, and adjustment techniques were not used.	The intervention in this study was implemented at the population level.	Deviations from the intended intervention did not occur.	There were no missing data.	Due to population-level interventions, outcome assessors were not blinded.	The outcomes of interest and the effect estimates were not reported across multiple measurements or analyses.	
Sacre et al. (2021) [76]	Critical risk	Serious risk	Low risk	Low risk	Low risk	Moderate risk	Low risk	Critical risk of bias
	All important confounding domains were not controlled.	The start of follow-up and the start of the intervention did not coincide, and adjustment techniques were not used.	The intervention in this study was implemented at the population level.	Deviations from the intended intervention did not occur.	There were no missing data.	Due to population-level interventions, outcome assessors were not blinded.	The outcomes of interest and the effect estimates were not reported across multiple measurements or analyses.	
Shoshani et al. (2021) [77]	Critical risk	Serious risk	Low risk	Moderate risk	Moderate risk	Moderate risk	Low risk	Critical risk of bias
	All important confounding domains were not controlled, and cluster-level confounding was not accounted for.	The start of follow-up and the start of the intervention did not coincide, and adjustment techniques were not used.	The intervention in this study was implemented at the population level.	Before and after the intervention, data were collected from the same individuals. However, a total of 115 students did not complete the post-intervention assessment. These deviations from intended interventions were not considered to reflect usual practice.	In this study, less than 3% of data were missing per item for both measurement points, and missing values were imputed using expectation maximization procedures.	Due to population-level interventions, outcome assessors were not blinded.	The outcomes of interest and the effect estimates were not reported across multiple measurements or analyses.	
van der Velden et al. (2022) [79]	Critical risk	Serious risk	Low risk	Low risk	Moderate risk	Moderate risk	Low risk	Critical risk of bias
	All important confounding domains were not controlled.	The start of follow-up and the start of the intervention did not coincide, and adjustment techniques were not used.	The intervention in this study was implemented at the population level.	Deviations from the intended intervention did not occur.	60 out of 1128 participants in the intervention group and 114 out of 1128 participants in the control group were excluded due to incomplete data, and the robustness of the analyses was not assessed.	Due to population-level interventions, outcome assessors were not blinded.	The outcomes of interest and the effect estimates were not reported across multiple measurements or analyses.	
van den Besselaar et al. (2021) [80]	Critical risk	Serious risk	Low risk	Low risk	Low risk	Moderate risk	Low risk	Critical risk of bias
	All important confounding domains were not controlled.	The start of follow-up and the start of the intervention did not coincide, and adjustment techniques were not used.	The intervention in this study was implemented at the population level.	Deviations from the intended intervention did not occur.	There were no missing data.	Due to population-level interventions, outcome assessors were not blinded.	The outcomes of interest and the effect estimates were not reported across multiple measurements or analyses.	
Yang et al. (2021) [81]	Critical risk	Serious risk	Low risk	Low risk	Low risk	Moderate risk	Moderate risk	Critical risk of bias
	All important confounding domains were not controlled, and cluster-level confounding was not accounted for.	The start of follow-up and the start of the intervention did not coincide, and adjustment techniques were not used.	The intervention in this study was implemented at the population level.	Deviations from the intended intervention did not occur.	There were no missing data.	Due to population-level interventions, outcome assessors were not blinded.	It was unclear whether the a priori analysis was specified, and some multivariate analyses were shown.	
Zijlmans et al. (2023) [82]	Critical risk	Serious risk	Low risk	Serious risk	No information	Moderate risk	Low risk	Critical risk of bias
	All important confounding domains were not controlled, and cluster-level confounding was not accounted for.	The start of follow-up and the start of the intervention did not coincide, and adjustment techniques were not used.	The intervention in this study was implemented at the population level.	Although this was a longitudinal study of the target cohort, the post-intervention participants were selected by random sampling.	There was no information on missing data.	Due to population-level interventions, outcome assessors were not blinded.	The outcomes of interest and the effect estimates were not reported across multiple measurements or analyses.	

Supplementary material 7 

**Table 10:** Detailed assessment of the risk of bias for suicide

Study	Bias due to confounding	Bias in the selection of participants for the study	Bias in the classification of intervention	Bias due to deviations from intended intervention(s)	Bias due to missing data	Bias in the measurement of outcomes	Bias in the selection of the reported results	Overall risk of bias
Acharya et al. (2022) [41]	Critical risk	Serious risk	Low risk	Low risk	Low risk	Low risk	Low risk	Critical risk of bias
	All important confounding domains were not controlled.	In this study, the start of follow-up and the start of the intervention coincided. However, the incidence of suicide was assessed before and one year and three months after the intervention, and adjustment techniques for selection bias were not used.	The intervention in this study was implemented at the population level.	Deviations from the intended intervention did not occur.	Data in this study were obtained from the Nepal Police Headquarters, Kathmandu, Nepal, and the possibility of missing data was considered low.	Since the population-level intervention was implemented, the outcome assessors were not blinded, but this does not affect the outcome results.	The outcomes of interest and the effect estimates were not reported across multiple measurements or analyses.	
Tanaka et al. (2021) [78]	Critical risk	Serious risk	Low risk	Low risk	Low risk	Low risk	Low risk	Critical risk of bias
	All important confounding domains were not controlled.	In this study, the start of follow-up and the start of the intervention coincided. However, the incidence of suicide was assessed before and three to six months after the intervention, and adjustment techniques for selection bias were not used.	The intervention in this study was implemented at the population level.	Deviations from the intended intervention did not occur.	Data in this study were obtained from a city-by-month-level dataset in Japan, and the possibility of missing data was considered low.	Since the population-level intervention was implemented, the outcome assessors were not blinded, but this does not affect the outcome results.	The outcomes of interest and the effect estimates were not reported across multiple measurements or analyses.	

Supplementary material 8 

Supplementary material 9 

**Table 11:** Detailed assessment of the risk of bias for anxiety symptom severity

Study	Bias due to confounding	Bias in the selection of participants for the study	Bias in the classification of interventions	Bias due to deviations from intended interventions	Bias due to missing data	Bias in the measurement of outcomes	Bias in the selection of the reported results	Overall risk of bias
Arad et al. (2021) [43]	Critical risk	Serious risk	Low risk	Low risk	Low risk	Moderate risk	Moderate risk	Critical risk of bias
	All important confounding domains were not controlled.	The start of the follow-up and the start of the intervention did not coincide, and adjustment techniques were not used.	The intervention in this study was implemented at the population level.	Deviations from the intended intervention did not occur.	There were no missing data.	Due to population-level interventions, outcome assessors were not blinded.	In this study, there was no pre-registered protocol, and multiple effect estimates were reported.	
Bartlett et al. (2021) [45]	Serious risk	Serious risk	Low risk	Low risk	Serious risk	Moderate risk	Low risk	Serious risk of bias
	This study was needed to assess baseline confounding factors, but it only controlled for two of the three important areas of confounding.	The start of the follow-up and the start of the intervention did not coincide, and adjustment techniques were not used.	The intervention in this study was implemented at the population level.	Deviations from the intended intervention did not occur.	In both groups, 2611 out of 4282 participants were excluded due to missing data, and the robustness of the analyses was not assessed.	Due to population-level interventions, outcome assessors were not blinded.	The outcomes of interest and the effect estimates were not reported across multiple measurements or analyses.	
Bennett et al. (2022) [46]	Critical risk	Serious risk	Low risk	Low risk	Low risk	Moderate risk	Low risk	Critical risk of bias
	All important confounding domains were not controlled.	The start of the follow-up and the start of the intervention did not coincide, and adjustment techniques were not used.	The intervention in this study was implemented at the population level.	Deviations from the intended intervention did not occur.	There were no missing data.	Due to population-level interventions, outcome assessors were not blinded.	The outcomes of interest and the effect estimates were not reported across multiple measurements or analyses.	
Bouter et al. (2023) [49]	Critical risk	Serious risk	Low risk	Low risk	Low risk	Moderate risk	Moderate risk	Critical risk of bias
	All important confounding domains were not controlled.	The start of the follow-up and the start of the intervention did not coincide, and adjustment techniques were not used.	The intervention in this study was implemented at the population level.	Deviations from the intended intervention did not occur.	There were no missing data.	Due to population-level interventions, outcome assessors were not blinded.	In this study, there was no pre-registered protocol, and multiple effect estimates were reported.	
Cohen et al. (2021) [54]	Serious risk	Serious risk	Low risk	Low risk	Low risk	Moderate risk	Low risk	Serious risk of bias
	Only one important confounding domain was controlled.	The start of the follow-up and the start of the intervention did not coincide, and adjustment techniques were not used.	The intervention in this study was implemented at the population level.	Deviations from the intended intervention did not occur.	There were no missing data.	Due to population-level interventions, outcome assessors were not blinded.	The outcomes of interest and the effect estimates were not reported across multiple measurements or analyses.	
Dunn et al. (2021) [56]	Critical risk	Serious risk	Low risk	Low risk	Serious risk	Moderate risk	Low risk	Critical risk of bias
	All important confounding domains were not controlled.	The start of the follow-up and the start of the intervention did not coincide, and adjustment techniques were not used.	The intervention in this study was implemented at the population level.	Deviations from the intended intervention did not occur.	The analysis included 45 participants from the intervention group out of the total 48 participants, with the exclusion due to missing data. Robustness of the results was not confirmed in the analyses.	Due to population-level interventions, outcome assessors were not blinded.	The outcomes of interest and the effect estimates were not reported across multiple measurements or analyses.	
Gonzalez-Martinez et al. (2021) [57]	Critical risk	Serious risk	Low risk	Low risk	Low risk	Moderate risk	Low risk	Critical risk of bias
	All important confounding domains were not controlled.	The start of the follow-up and the start of the intervention did not coincide, and adjustment techniques were not used.	The intervention in this study was implemented at the population level.	Deviations from the intended intervention did not occur.	There were no missing data.	Due to population-level interventions, outcome assessors were not blinded.	The outcomes of interest and the effect estimates were not reported across multiple measurements or analyses.	
Hausman et al. (2022) [58]	Critical risk	Serious risk	Low risk	Low risk	Serious risk	Moderate risk	Low risk	Critical risk of bias
	All important confounding domains were not controlled.	The start of the follow-up and the start of the intervention did not coincide, and adjustment techniques were not used.	The intervention in this study was implemented at the population level.	Deviations from the intended intervention did not occur.	The analysis included 170 participants from the intervention group out of the total 189 participants, with the exclusion due to missing data. Robustness of the results was not confirmed in the analyses conducted.	Due to population-level interventions, outcome assessors were not blinded.	The outcomes of interest and the effect estimates were not reported across multiple measurements or analyses.	
Lee et al. (2020) [63]	Critical risk	Serious risk	Low risk	Low risk	Low risk	Moderate risk	Low risk	Critical risk of bias
	All important confounding domains were not controlled.	The start of the follow-up and the start of the intervention did not coincide, and adjustment techniques were not used.	The intervention in this study was implemented at the population level.	Deviations from the intended intervention did not occur.	There were no missing data.	Due to population-level interventions, outcome assessors were not blinded.	The outcomes of interest and the effect estimates were not reported across multiple measurements or analyses.	
Macfarlane et al. (2021) [66]	Critical risk	Serious risk	Low risk	No information	No information	Moderate risk	Low risk	Critical risk of bias
	All important confounding domains were not controlled.	The start of the follow-up and the start of the intervention did not coincide, and adjustment techniques were not used.	The intervention in this study was implemented at the population level.	There was no information on deviations from the intended intervention.	There was no information on missing data.	Due to population-level interventions, outcome assessors were not blinded.	The outcomes of interest and the effect estimates were not reported across multiple measurements or analyses.	
Meda et al. (2021) [68]	Critical risk	Serious risk	Low risk	Low risk	Moderate risk	Moderate risk	Low risk	Critical risk of bias
	All important confounding domains were not controlled.	The start of the follow-up and the start of the intervention did not coincide, and adjustment techniques were not used.	The intervention in this study was implemented at the population level.	Deviations from the intended intervention did not happen.	In both groups, 161 out of 358 participants were excluded due to incomplete data, and the robustness of the performed analyses has not been examined.	Due to population-level interventions, outcome assessors were not blinded.	The outcomes of interest and the effect estimates were not reported across multiple measurements or analyses.	
Minhas et al. (2021) [69]	Serious risk	Serious risk	Low risk	Low risk	Low risk	Moderate risk	Low risk	Serious risk of bias
	Only one important confounding domain was controlled.	The start of the follow-up and the start of the intervention did not coincide, and adjustment techniques were not used.	The intervention in this study was implemented at the population level.	Deviations from the intended intervention did not occur.	There were no missing data.	Due to population-level interventions, outcome assessors were not blinded.	The outcomes of interest and the effect estimates were not reported across multiple measurements or analyses.	
Moya et al. (2021) [70]	Serious risk	Serious risk	Low risk	Low risk	Low risk	Moderate risk	Low risk	Serious risk of bias
	Only one important confounding domain was controlled.	The start of the follow-up and the start of the intervention did not coincide, and adjustment techniques were not used.	The intervention in this study was implemented at the population level.	Deviations from the intended intervention did not occur.	There were no missing data.	Due to population-level interventions, outcome assessors were not blinded.	The outcomes of interest and the effect estimates were not reported across multiple measurements or analyses.	
Murphy et al. (2023) [71]	Serious risk	Serious risk	Low risk	Low risk	Low risk	Moderate risk	Low risk	Serious risk of bias
	Only one important confounding domain was controlled.	The start of the follow-up and the start of the intervention did not coincide, and adjustment techniques were not used.	The intervention in this study was implemented at the population level.	Deviations from the intended intervention did not occur.	There were no missing data.	Due to population-level interventions, outcome assessors were not blinded.	The outcomes of interest and the effect estimates were not reported across multiple measurements or analyses.	
Overbeck et al. (2021) [72]	Serious risk	Serious risk	Low risk	Low risk	Serious risk	Moderate risk	Low risk	Serious risk of bias
	This study was needed to assess baseline confounding factors, yet it controlled for only two of the three important domains of confounding.	The start of the follow-up and the start of the intervention did not coincide, and adjustment techniques were not used.	The intervention in this study was implemented at the population level.	Deviations from the intended intervention did not occur.	In the control group, 102 out of 1428 participants were excluded due to missing data. In the intervention group, there were no missing data.	Due to population-level interventions, outcome assessors were not blinded.	The outcomes of interest and the effect estimates were not reported across multiple measurements or analyses.	
Rimfeld et al. (2022) [74]	Critical risk	Serious risk	Low risk	Low risk	Low risk	Moderate risk	Low risk	Critical risk of bias
	All important confounding domains were not controlled, and cluster-level confounding was not accounted for.	The start of the follow-up and the start of the intervention did not coincide, and adjustment techniques were not used.	The intervention in this study was implemented at the population level.	Deviations from the intended intervention did not occur.	There were no missing data.	Due to population-level interventions, outcome assessors were not blinded.	The outcomes of interest and the effect estimates were not reported across multiple measurements or analyses.	
Sacre et al. (2021) [76]	Critical risk	Serious risk	Low risk	Low risk	Low risk	Moderate risk	Low risk	Critical risk of bias
	All important confounding domains were not controlled.	The start of the follow-up and the start of the intervention did not coincide, and adjustment techniques were not used.	The intervention in this study was implemented at the population level.	Deviations from the intended intervention did not occur.	There were no missing data.	Due to population-level interventions, outcome assessors were not blinded.	The outcomes of interest and the effect estimates were not reported across multiple measurements or analyses.	
Shoshani et al. (2021) [77]	Critical risk	Serious risk	Low risk	Moderate risk	Moderate risk	Moderate risk	Low risk	Critical risk of bias
	All important confounding domains were not controlled, and cluster-level confounding was not accounted for.	The start of the follow-up and the start of the intervention did not coincide, and adjustment techniques were not used.	The intervention in this study was implemented at the population level.	Before and after the intervention, data were collected from the same individuals. However, a total of 115 students did not complete the post-intervention assessment. These deviations from intended interventions were not considered to reflect usual practice.	In this study, less than 3% of data were missing per item for both measurement points, and missing values were imputed using expectation maximization procedures.	Due to population-level interventions, outcome assessors were not blinded.	The outcomes of interest and the effect estimates were not reported across multiple measurements or analyses.	
van der Velden et al. (2022) [79]	Critical risk	Serious risk	Low risk	Low risk	Moderate risk	Moderate risk	Low risk	Critical risk of bias
	All important confounding domains were not controlled.	The start of the follow-up and the start of the intervention did not coincide, and adjustment techniques were not used.	The intervention in this study was implemented at the population level.	Deviations from the intended intervention did not occur.	60 out of 1128 participants in the intervention group and 114 out of 1128 participants in the control group were excluded due to incomplete data, and the robustness of the performed analyses has not been examined.	Due to population-level interventions, outcome assessors were not blinded.	The outcomes of interest and the effect estimates were not reported across multiple measurements or analyses.	
Zijlmans et al. (2023) [82]	Critical risk	Serious risk	Low risk	Serious risk	No information	Moderate risk	Low risk	Critical risk of bias
	All important confounding domains were not controlled, and cluster-level confounding was not accounted for.	The start of the follow-up and the start of the intervention did not coincide, and adjustment techniques were not used.	The intervention in this study was implemented at the population level.	Although this was a longitudinal study of the target cohort, the post-intervention participants were selected by random sampling.	There was no information on missing data.	Due to population-level interventions, outcome assessors were not blinded.	The outcomes of interest and the effect estimates were not reported across multiple measurements or analyses.	

Supplementary material 10 

Supplementary material 11

**Table 12:** Detailed assessment of post-traumatic stress disorder symptom severity

Study	Bias due to confounding	Bias in the selection of participants for the study	Bias in the classification of interventions	Bias due to deviations from intended interventions	Bias due to missing data	Bias in the measurement of outcomes	Bias in the selection of the reported results	Overall risk of bias
Berthelot et al. (2020) [47]	Serious risk	Serious risk	Low risk	Low risk	Low risk	Moderate risk	Low risk	Serious risk of bias
	Only one important confounding domain was controlled.	The start of the follow-up and the start of the intervention did not coincide, and adjustment techniques were not used.	The intervention in this study was implemented at the population level.	Deviations from the intended intervention did not occur.	There were no missing data.	Due to population-level interventions, outcome assessors were not blinded.	The outcomes of interest and the effect estimates were not reported across multiple measurements or analyses.	
van der Velden et al. (2022) [79]	Critical risk	Serious risk	Low risk	Low risk	Low risk	Moderate risk	Low risk	Critical risk of bias
	All important confounding domains were not controlled.	The start of the follow-up and the start of the intervention did not coincide, and adjustment techniques were not used.	The intervention in this study was implemented at the population level.	Deviations from the intended intervention did not occur.	There were no missing data.	Due to population-level interventions, outcome assessors were not blinded.	The outcomes of interest and the effect estimates were not reported across multiple measurements or analyses.	

Supplementary material 12 

Supplementary material 13

**Table 13:** Detailed assessment of the risk of bias for insomnia symptom severity

Study ID	Bias due to confounding	Bias in the selection of participants for the study	Bias in the classification of interventions	Bias due to deviations from intended interventions	Bias due to missing data	Bias in the measurement of outcomes	Bias in the selection of the reported results	Overall risk of bias
Cellini et al. (2021) [51]	Serious risk	Serious risk	Low risk	Low risk	Low risk	Moderate risk	Low risk	Serious risk of bias
	Only one important confounding domain was controlled.	The start of the follow-up and the start of the intervention did not coincide, and adjustment techniques were not used.	The intervention in this study was implemented at the population level.	Deviations from the intended intervention did not occur.	There were no missing data.	Due to population-level interventions, outcome assessors were not blinded.	The outcomes of interest and the effect estimates were not reported across multiple measurements or analyses.	
Cellini et al. (2021) [52]	Serious risk	Serious risk	Low risk	Low risk	Low risk	Moderate risk	Low risk	Serious risk of bias
	Only one important confounding domain was controlled.	The start of the follow-up and the start of the intervention did not coincide, and adjustment techniques were not used.	The intervention in this study was implemented at the population level.	Deviations from the intended intervention did not occur.	There were no missing data.	Due to population-level interventions, outcome assessors were not blinded.	The outcomes of interest and the effect estimates were not reported across multiple measurements or analyses.	
Cody et al. (2021) [53]	Critical risk	Serious risk	Low risk	Low risk	Low risk	Moderate risk	Low risk	Critical risk of bias
	All important confounding domains were not controlled.	The start of the follow-up and the start of the intervention did not coincide, and adjustment techniques were not used.	The intervention in this study was implemented at the population level.	Deviations from the intended intervention did not occur.	There were no missing data.	Due to population-level interventions, outcome assessors were not blinded.	The outcomes of interest and the effect estimates were not reported across multiple measurements or analyses.	
Gonzalez-Martinez et al. (2021) [57]	Critical risk	Serious risk	Low risk	Low risk	low risk	Moderate risk	Low risk	Critical risk of bias
	All important confounding domains were not controlled.	The start of the follow-up and the start of the intervention did not coincide, and adjustment techniques were not used.	The intervention in this study was implemented at the population level.	Deviations from the intended intervention did not occur.	Only one participant in the control group was excluded due to missing data, and any bias due to this was negligible.	Due to population-level interventions, outcome assessors were not blinded.	The outcomes of interest and the effect estimates were not reported across multiple measurements or analyses.	
Hausman et al. (2022) [58]	Critical risk	Serious risk	Low risk	Low risk	Low risk	Moderate risk	Low risk	Critical risk of bias
	All important confounding domains were not controlled.	The start of the follow-up and the start of the intervention did not coincide, and adjustment techniques were not used.	The intervention in this study was implemented at the population level.	Deviations from the intended intervention did not occur.	There were no missing data.	Due to population-level interventions, outcome assessors were not blinded.	The outcomes of interest and the effect estimates were not reported across multiple measurements or analyses.	
Macfarlane et al. (2021) [66]	Critical risk	Serious risk	Low risk	No information	No information	Moderate risk	Low risk	Critical risk of bias
	All important confounding domains were not controlled.	The start of the follow-up and the start of the intervention did not coincide, and adjustment techniques were not used.	The intervention in this study was implemented at the population level.	There was no information on deviations from the intended intervention.	There was no information on missing data.	Due to population-level interventions, outcome assessors were not blinded.	The outcomes of interest and the effect estimates were not reported across multiple measurements or analyses.	
Romdhani et al. (2022) [75]	Critical risk	Serious risk	low risk	Low risk	Low risk	Moderate risk	Low risk	Critical risk of bias
	All important confounding domains were not controlled, and cluster-level confounding was not accounted for.	The start of the follow-up and the start of the intervention did not coincide, and adjustment techniques were not used.	The intervention in this study was implemented at the population level.	Deviations from the intended intervention did not occur.	There were no missing data.	Due to population-level interventions, outcome assessors were not blinded.	The outcomes of interest and the effect estimates were not reported across multiple measurements or analyses.	
Zijlmans et al. (2023) [82]	Critical risk	Serious risk	Low risk	Serious risk	No information	Moderate risk	Low risk	Critical risk of bias
	All important confounding domains were not controlled, and cluster-level confounding was not accounted for.	The start of the follow-up and the start of the intervention did not coincide, and adjustment techniques were not used.	The intervention in this study was implemented at the population level.	Although this was a longitudinal study of the target cohort, the post-intervention participants were selected by random sampling.	There was no information on missing data.	Due to population-level interventions, outcome assessors were not blinded.	The outcomes of interest and the effect estimates were not reported across multiple measurements or analyses.	

Supplementary material 14

Supplementary material 15 

**Table 14:** Detailed assessment of the risk of bias for substance use

Study	Bias due to confounding	Bias in the selection of participants for the study	Bias in the classification of interventions	Bias due to deviations from intended interventions	Bias due to missing data	Bias in the measurement of outcomes	Bias in the selection of the reported results	Overall risk of bias
Albrecht et al. (2022) [42]	Critical risk	Serious risk	Low risk	Low risk	Serious risk	Moderate risk	Low risk	Critical risk of bias
	All important confounding domains were not controlled.	The start of the follow-up and the start of the intervention did not coincide, and adjustment techniques were not used.	The intervention in this study was implemented at the population level.	Deviations from the intended intervention did not occur.	296 out of 404 participants in the intervention group and 197 out of 402 in the control group were excluded due to missing data, and robustness in the analyses was not confirmed.	Due to population-level interventions, outcome assessors were not blinded.	The outcomes of interest and the effect estimates were not reported across multiple measurements or analyses.	
Barbosa et al. (2021) [44]	Serious risk	Serious risk	Low risk	Low risk	Low risk	Moderate risk	Low risk	Serious risk of bias
	Only one important confounding domain was controlled.	The start of the follow-up and the start of the intervention did not coincide, and adjustment techniques were not used.	The intervention in this study was implemented at the population level.	Deviations from the intended intervention did not occur.	There were no missing data.	Due to population-level interventions, outcome assessors were not blinded.	The outcomes of interest and the effect estimates were not reported across multiple measurements or analyses.	
Bartlett et al. (2021) [45]	Serious risk	Serious risk	Low risk	Low risk	Serious risk	Moderate risk	Low risk	Serious risk of bias
	Two important confounding domains were controlled.	The start of the follow-up and the start of the intervention did not coincide, and adjustment techniques were not used.	The intervention in this study was implemented at the population level.	Deviations from the intended intervention did not occur.	Among the total participants (4282), analyses were conducted on 1671 participants in both groups due to missing data, and robustness was not confirmed in the analyses performed.	Due to population-level interventions, outcome assessors were not blinded.	The outcomes of interest and the effect estimates were not reported across multiple measurements or analyses.	
Burdzovic Andreas and Brunborg (2022) [50]	Serious risk	Serious risk	Low risk	Low risk	Low risk	Moderate risk	Low risk	Serious risk of bias
	Two important confounding domains were controlled.	The start of the follow-up and the start of the intervention did not coincide, and adjustment techniques were not used.	The intervention in this study was implemented at the population level.	Deviations from the intended intervention did not occur.	There were no missing data.	Due to population-level interventions, outcome assessors were not blinded.	The outcomes of interest and the effect estimates were not reported across multiple measurements or analyses.	
Cousijn et al. (2021) [55]	Critical risk	Serious risk	Low risk	Moderate risk	Moderate risk	Moderate risk	Low risk	Critical risk of bias
	All important confounding domains were not controlled.	The start of the follow-up and the start of the intervention did not coincide, and adjustment techniques were not used.	The intervention in this study was implemented at the population level.	Before and after the intervention, data were collected from the same individuals. However, 844 out of 1030 participants did not complete the post-intervention assessment. These deviations from intended interventions were not considered to reflect usual practice.	In both groups, 11 out of 120 participants were excluded due to missing data, and the robustness of the performed analyses has not been assessed.	Due to population-level interventions, outcome assessors were not blinded.	The outcomes of interest and the effect estimates were not reported across multiple measurements or analyses.	
Kekäläinen et al. (2021) [59]	Serious risk	Serious risk	Low risk	Low risk	Low risk	Moderate risk	Moderate risk	Serious risk of bias
	This study was needed to assess baseline confounding factors, yet it controlled for only two of the three important domains of confounding.	The start of the follow-up and the start of the intervention did not coincide, and adjustment techniques were not used.	The intervention in this study was implemented at the population level.	Deviations from the intended intervention did not occur.	There were no missing data.	Due to population-level interventions, outcome assessors were not blinded.	In this study, there was no pre-registered protocol, and multiple effect estimated were reported.	
Leatherdale et al. (2023) [62]	Critical risk	Serious risk	Low risk	Moderate risk	Moderate risk	Moderate risk	Low risk	Critical risk of bias
	All important confounding domains were not controlled.	The start of the follow-up and the start of the intervention did not coincide, and adjustment techniques were not used.	The intervention in this study was implemented at the population level.	After the intervention, 5554 out of 7653 participants were deviated from the intended intervention, and the deviations were unbalanced and likely to affect outcomes.	150 out of 2099 participants in the intervention group and 68 out of 7585 in the control group were excluded due to missing data, and robustness was not confirmed in the analyses performed.	Due to population-level interventions, outcome assessors were not blinded.	The outcomes of interest and the effect estimates were not reported across multiple measurements or analyses.	
Minhas et al. (2021) [69]	Serious risk	Serious risk	Low risk	Low risk	Low risk	Moderate risk	Low risk	Serious risk of bias
	Only one important confounding domain was controlled.	The start of the follow-up and the start of the intervention did not coincide, and adjustment techniques were not used.	The intervention in this study was implemented at the population level.	Deviations from the intended intervention did not occur.	There were no missing data.	Due to population-level interventions, outcome assessors were not blinded.	The outcomes of interest and the effect estimates were not reported across multiple measurements or analyses.	
Pelham et al. (2022) [73]	Critical risk	Serious risk	Low risk	Low risk	Serious risk	Moderate risk	Low risk	Critical risk of bias
	All important confounding domains were not controlled.	The start of the follow-up and the start of the intervention did not coincide, and adjustment techniques were not used.	The intervention in this study was implemented at the population level.	Deviations from the intended intervention did not occur.	In the intervention group, 135 out of 348 participants were excluded due to missing data, while, in the control group, 67 participants were excluded. Robustness in the analyses was not confirmed.	Due to population-level interventions, outcome assessors were not blinded.	The outcomes of interest and the effect estimates were not reported across multiple measurements or analyses.	

Supplementary material 16 
